# Study on microstructure and properties of titanium alloy wall fabricated by laser-TIG arc

**DOI:** 10.1371/journal.pone.0355487

**Published:** 2026-09-15

**Authors:** Mengyao Li, Yongchun Guo, Su Wang

**Affiliations:** 1 School of Aviation Maintenance Engineering, Xi’an Aeronautical Polytechnic Institute, Xi’an, China; 2 School of Aeronautical Manufacture Engineering, Xi’an Aeronautical Polytechnic Institute, Xi’an, China; 3 Northeastern University, Shenyang, China; UNICAMP, University of Campinas, BRAZIL

## Abstract

Using TC4 titanium alloy welding wire as raw material, through laser-TIG arc hybrid heat source wire additive manufacturing technology, combined with reciprocating scanning method for deposition, the effects of different process parameters on the tensile properties, impact properties and hardness of additive specimens were studied. The results show that: (1) With the increase of laser power, the average layer width of the formed part increases from 7.1 mm to 12.1 mm, an increase of 59%; the average layer height decreased from 1.95 mm to 1.56 mm, a decrease of 20%. (2) When the laser power is 1100 W, the transverse tensile strength is 896 MPa, the longitudinal tensile strength is 883 MPa, and the difference between transverse and longitudinal tensile strength is 13 MPa. The elongation after transverse fracture is 9.31%, the elongation after longitudinal fracture is 10.23%, and the difference of elongation after transverse and longitudinal fracture is 0.92%. (3) The impact energy of the additive samples at room temperature (25°C) in two directions is 48.5 J·cm^−^² (X direction) and 52.2K J·cm^−^² (Y direction), respectively, and the microhardness of the top of the wall is higher than that of the middle end of the wall. This study provides an important experimental basis for the engineering application of laser-TIG arc additive manufacturing technology of TC4 titanium alloy.

## 1. Introduction

Titanium and its alloys have a series of excellent properties, such as low density, high specific strength and specific stiffness, corrosion resistance and fatigue resistance, high temperature performance and good welding performance, and are praised as the third metal. With the rapid development of aviation and other industries, the requirements for titanium alloy performance indicators are becoming more and more stringent. In order to meet the use standards of key structural parts such as advanced aero-engines, the development of efficient preparation processes for titanium alloys has become one of the core research hotspots in the field of materials for a long time now and in the future [1–8].

Many scholars at home and abroad have done a lot of research on additive manufacturing technology. The study of Akerfeldt *et al*.[9] shows that the coarse columnar β grains grown along the additive direction are one of the main factors affecting the anisotropy of the additive components. Edwards *et al*.[10] analyzed the effect of pores on the overall mechanical properties of the samples fabricated by laser deposition. Razavi *et al*.[11] used TSLM technology to prepare Ti-6Al-4V titanium alloy, and found that the surface quality of the workpiece produced by this method is high, and the defects are all on the surface of the workpiece. Brand *et al*.[12] carried out heat treatment on the subsequent laser additive manufacturing TC4 titanium alloy samples, and found that the mechanical properties of the samples were improved and the strength was improved. Baufeld *et al*.[13] found that the upper limit of fatigue strength of titanium alloys fabricated by additive manufacturing was increased by heat treatment. On this basis, Vrancken *et al*.[14] increased the temperature of heat treatment. When the transition temperature of a certain phase is reached, the mechanical properties of titanium alloys can be significantly improved. Zhang *et al*.[15] take a special temperature of titanium alloy annealing treatment, the sample, the material properties of uniform distribution, anisotropy has been significantly improved.

Pardal *et al*.[16] verified that the laser can stabilize the arc in the heat conduction mode, and the effective width utilization rate of the titanium alloy component is increased by 70.8%, which significantly improves the forming quality of the component. Rezaei A *et al*.[17,18] found that the grain size of LPBF aluminum alloy in keyhole mode was smaller and the proportion of low-angle grain boundaries was higher, which promoted the PLC effect and achieved three times of plasticity improvement. Cai *et al*.[19] prepared AZ31 magnesium alloy by laser-arc coaxial composite additive manufacturing technology. The average tensile strength in the horizontal and vertical directions is 224 MPa and 222 MPa, respectively, which is isotropic. Compared with the traditional single heat source additive manufacturing technology, the laser-TIG arc hybrid additive manufacturing technology can significantly improve the manufacturing efficiency, improve the microstructure morphology, refine the grains, and improve the comprehensive performance of the sample. Obviously, the laser-arc hybrid process has a significant positive impact on the forming quality, microstructure and properties of titanium alloy, stainless steel, aluminum alloy and other materials. However, there are few reports on the research of laser-arc hybrid additive manufacturing of titanium alloy.

In this thesis, TC4 titanium alloy, which is widely used in the field of aerospace engineering, is used as the main test material. Through the laser-TIG arc hybrid heat source wire additive manufacturing technology, combined with the reciprocating scanning method for single-layer deposition, the mechanism and influence of different process parameters on the tensile properties, impact properties and hardness of the additive test piece are studied. In the cause of solve the problems of difficult forming of titanium alloy and difficult processing of precision structural parts, the regulation of process parameters on the forming morphology and deposition efficiency of thin-walled dimensions is realized.

## 2. Materials and methods

**Fig 1:**
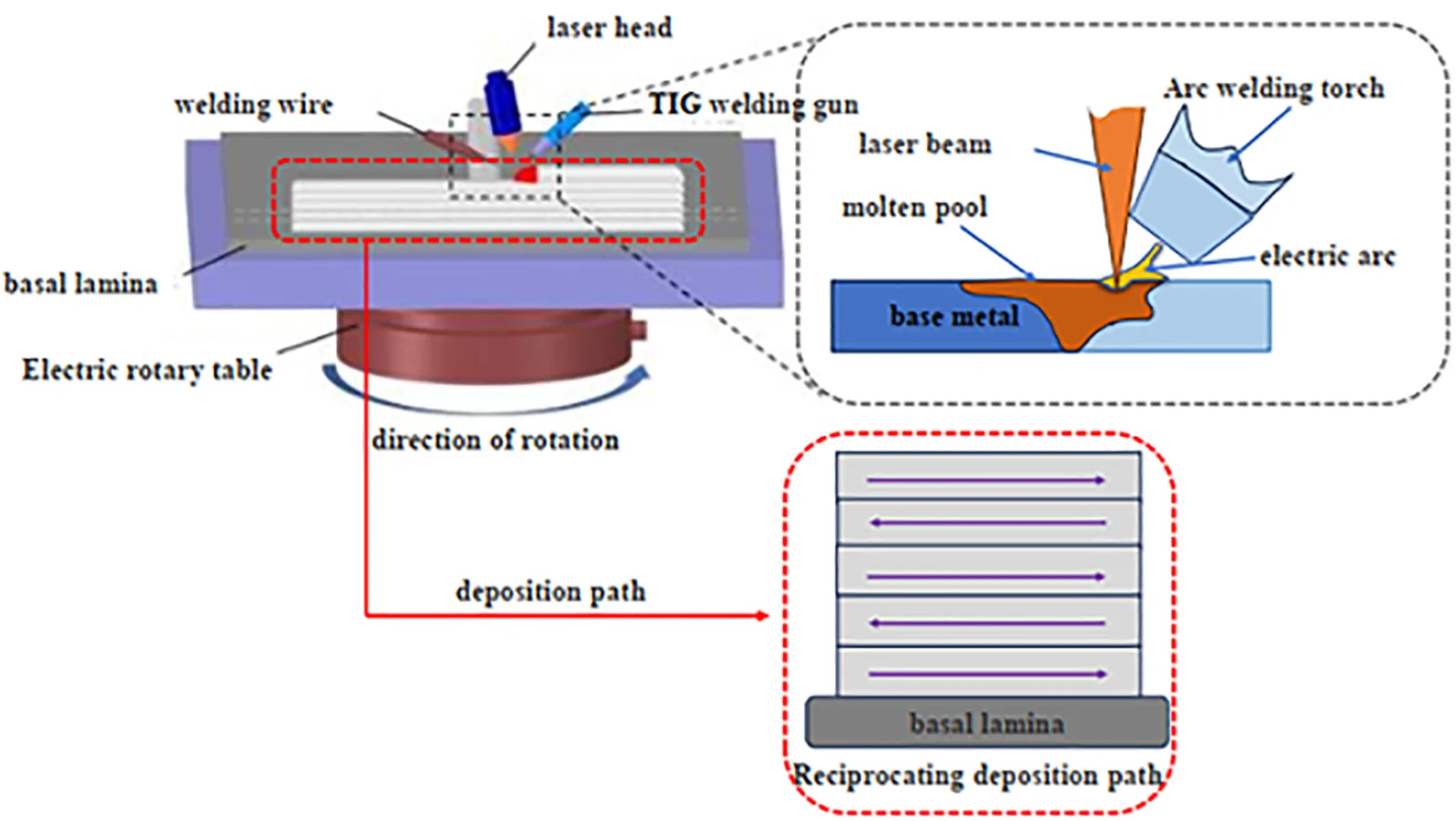
Schematic diagram of laser⁃arc hybrid additive manufacturing platform.

The TC4 wire with a diameter of 1.2 mm was used as the raw material, and the TC4 titanium alloy substrate was used as the substrate. The specific size was 150 mm × 200 mm × 20 mm. The chemical composition of the material is shown in Table 1. In this experiment, a laser-TIG arc hybrid additive manufacturing platform was built, and the schematic diagram is shown in Fig 1. The additive manufacturing platform mainly includes fiber laser, KUKA robot arm, wire feeder, TIG welding gun and vacuum protection box. The fiber laser uses a kilowatt YLS-10000-SS4 fiber laser produced by IPG company in the United States, with a focal length of 470 mm and a wavelength of 1070 ± 5 nm. The KUKA six-axis linkage robot arm is used to control the additive manufacturing process, which can accurately realize the planning of complex process paths. In the additive test, the deposition was carried out by reciprocating scanning, and 1 ~ 8 layers of walls were manufactured respectively. After the deposition of each layer, the next layer was deposited by air cooling for 40 s.

**Table 1:** Chemical composition of TC4 titanium alloy wire and substrate(mass fraction).

Ingredient	Al	V	N	O	Fe	C	Ti
**Wire**	6.02	3.91	0.01	0.09	0.17	0.08	Bal.
**Substrate**	6.03	4.02	0.02	0.12	0.15	0.02	Bal.

Before the test, the titanium wire wheel was used to clean the oxide film on the surface of the substrate, and acetone was used to remove oil and dust. The optimum laser power of laser-TIG arc hybrid wire additive manufacturing TC4 titanium alloy wall is 1100P/W, and other process window parameters are shown in Table 2. The core of substrate preheating in laser-arc additive manufacturing of titanium alloy is to reduce temperature gradient, suppress cracks, reduce residual stress, and improve microstructure and properties, which is the key process to ensure the forming quality. Therefore, the substrate is preheated at 150 °C for 8 min.

**Table 2:** Laser⁃arc hybrid additive manufacturing TC4 titanium alloy optimal process.

parameter	laser power *P/*W	welding current *I/*A	arc voltage *U/V*	wire feed rate *v*_*f*_*/*(m.min^-1^)	deposit velocity *v*_*w*_*/*(m.min^-1^)	laser-arc distance *D/*mm
numerical value	1100	156	20	6.0	0.3	1.5

The hardness test uses the German KB automatic Vickers hardness tester to test the microhardness of the section of the sample after grinding and polishing. According to GB/T4340.1-2009, the loading force used in the test is 200 g. The standard tensile specimens (parallel section 5 mm) were prepared according to GB/T 228.1–2021 in both vertical and horizontal directions, and the tensile test was carried out by INSTRON5985–250 kN material testing machine. V-shaped impact specimens (10 mm × 10 mm × 55 mm) were prepared according to GB/T 229–2020, and the impact test was carried out at room temperature (25 °C) by ZBC2602-B pendulum impact tester. For the microhardness test, three test points were taken at the same height of the sample to measure the hardness, and the average of the three test points was finally taken as the final test result. For tensile test and impact test, three samples are also tested respectively, and the average value of the three samples is the final test result.

## 3. Results and discussion

In this paper, single layer deposition is carried out by laser-TIG arc composite heat source wire additive manufacturing technology combined with reciprocating scanning mode. The data values of the average layer width and average layer height of the formed parts are shown in Table 3. The performance measurements of the samples in different directions are shown in Table 4. The measured hardness distribution of the deposited layer is shown in Table 5.

**Table 3:** The average layer width and average layer height data values of the formed parts.

Laser power/W	0	200	400	600	800	1000	1200	1400	1600
Average layer width/mm	7.10	7.90	8.50	9.00	9.50	10.0	10.7	11.0	12.1
Average layer height/mm	1.95	1.90	1.82	1.70	1.63	1.60	1.59	1.57	1.56

**Table 4:** The measured values of the sample in different directions.

Directions	X			Y		
	the first sample	the second sample	the third sample	the first sample	the second sample	the third sample
Tensile strength	896/MPa	898/MPa	900/MPa	883/MPa	885/MPa	888/MPa
Post-fracture elongation	9.13/-	9.19/-	9.2/-	10.23/-	10.32/-	10.3/-
Impact energy	48.5/J·cm^-^²	49.61/J·cm-²	49.64/J·cm-²	52.2/J·cm^-^²	53.35/J·cm-²	53.37/J·cm-²
Yield strength	734.51/ MPa	726.31/ MPa	740.21/ MPa	712.34/ MPa	705.71/ MPa	716.65/ MPa

**Table 5:** The hardness distribution value of the deposited layer.

Distance/mm	Middle/HV			Top/HV		
the first sample	the second sample	the third sample	the first sample	the second sample	the third sample
−5.0	380	383	384	420	422	423
−4.5	360	365	363	430	435	433
−4.0	355	359	360	425	430	426
−3.5	365	367	367	425	426	428
−3.0	380	382	383	430	432	434
−2.5	360	363	365	420	424	424
−2.0	385	389	388	405	410	409
−1.5	360	365	361	410	414	415
−1.0	380	382	384	415	420	418
−0.5	365	369	370	405	408	410
0.0	385	389	390	420	422	423
0.5	370	373	374	415	419	417
1.0	385	386	389	435	439	439
1.5	360	365	363	430	435	435
2.0	360	362	365	435	438	439
2.5	385	390	387	430	435	433
3.0	360	364	365	420	423	423
3.5	380	383	384	430	434	434
4.0	355	360	358	425	428	429
4.5	370	373	372	420	424	423
5.0	365	367	371	425	428	427
5.5	375	379	377	430	432	433

### 3.1. Macroscopic morphology analysis of laser-TIG arc hybrid wire additive manufacturing wall

**Fig 2:**
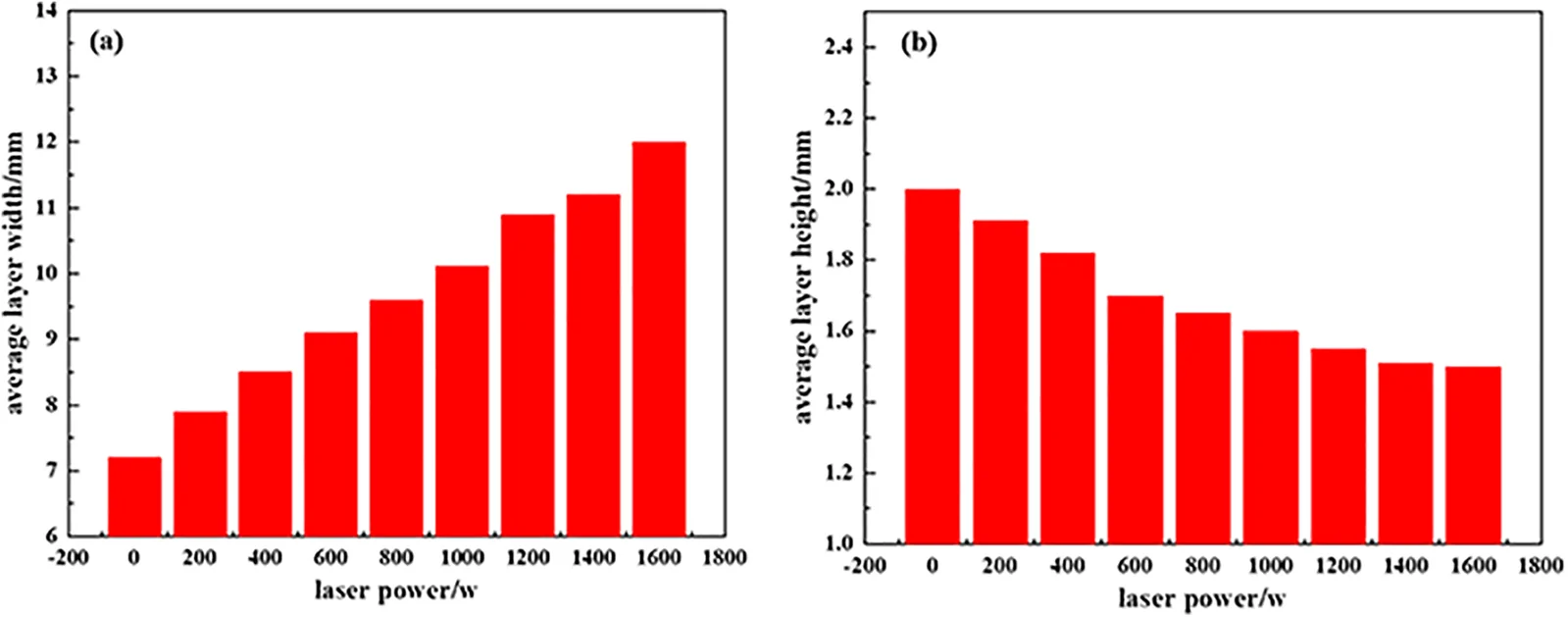
The effect of laser power: (a) the average layer width; (b) the average layer height.

When the laser power is 0W, 400W, 800W and 1600W, the influence of laser power on the average layer width and average layer height of the formed parts is discussed. The results are shown in Fig 2. It can be seen from the figure that when the laser power is 0W, 400W, 800W and 1600W, as the laser power increases, the average layer width of the formed part increases from 7.1 mm to 12.1 mm, an increase of 59%. The average layer height decreased from 1.95 mm to 1.56 mm, a decrease of 20%. When the laser power is 0W, 400W, 800W and 1600W, as the laser power increases, the heat input gradually increases, and the heat accumulation inside the formed part becomes larger, and the laser shock pool changes the fluidity of the molten pool, resulting in the overflow of the molten pool and the collapse of the molten pool, which in turn affects the average layer width and layer height of the wall of the formed part. Therefore, when the laser power is 0W, 400W, 800W and 1600W, with the increase of laser power, the average layer width of the formed wall increases gradually, and the average layer height decreases gradually.

### 3.2. Analysis of tensile properties of laser-TIG arc hybrid wire additive manufacturing wall

**Fig 3:**
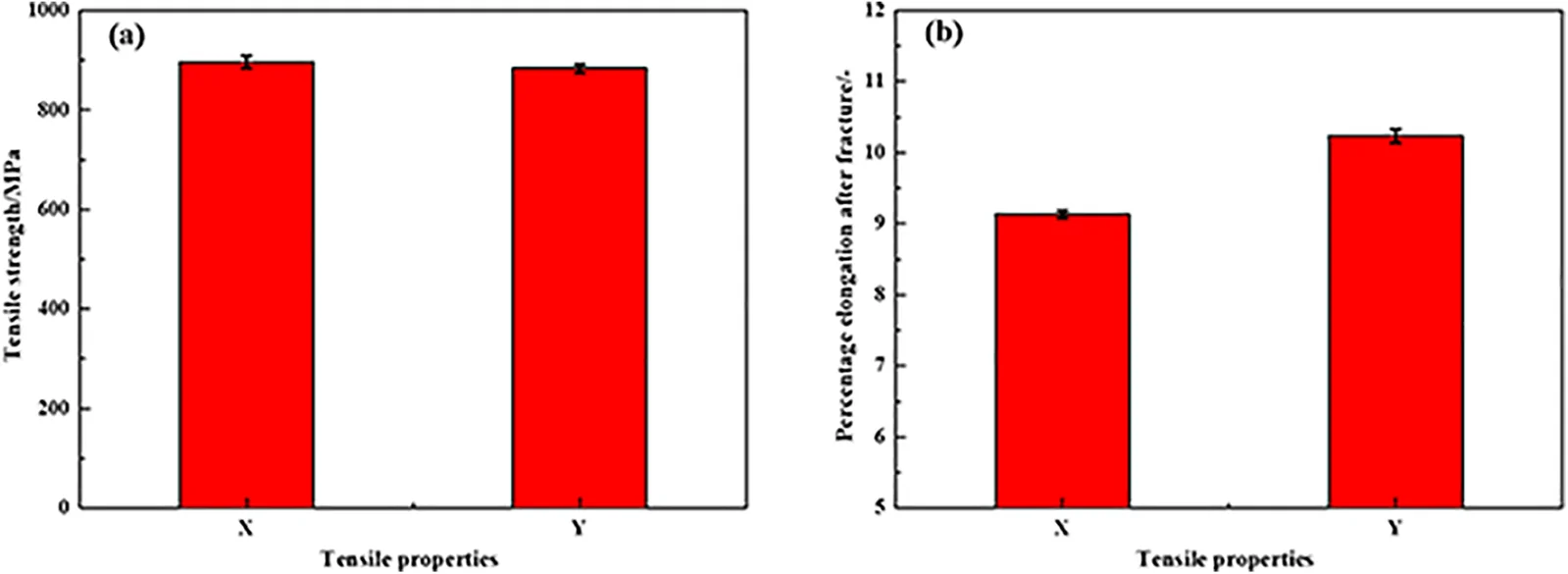
Analysis of tensile propertie: (a) tensile strength; (b) post-fracture elongation.

**Fig 4:**
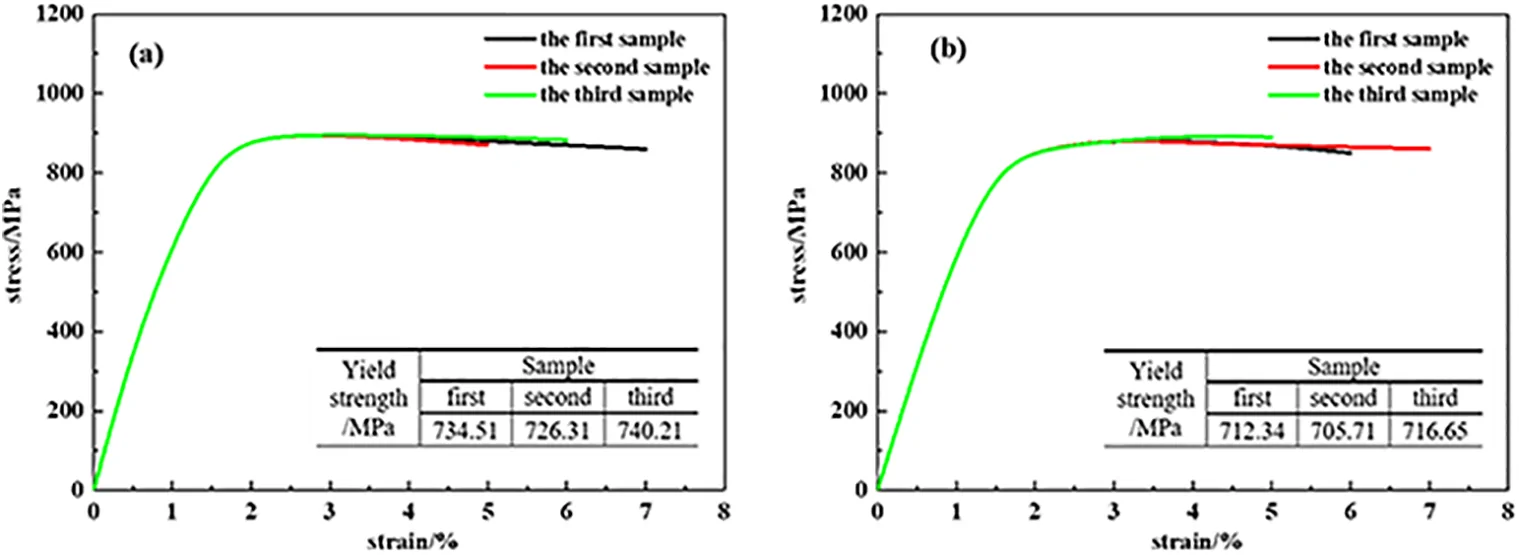
Stress-strain curves: (a) X-direction; (b) Y-direction.

When the laser power is 1100W, the tensile properties of TC4 titanium alloy in both transverse (X) and longitudinal (Y) directions are discussed. The results are shown in Fig 3. From Fig 3, it can be seen that when the laser power is 1100W, the transverse tensile strength is 896 MPa, the longitudinal tensile strength is 883 MPa, and the difference between the transverse and longitudinal tensile strength is 13 MPa; the transverse elongation after fracture is 9.31%, the longitudinal elongation after fracture is 10.23%, and the difference between transverse and longitudinal elongation after fracture is 0.92%, indicating that the anisotropy of tensile properties is small. This low anisotropy is mainly due to the reciprocating deposition method: the interlayer deposition directions are perpendicular to each other, so that the grain structures in different directions are similar, and the grain orientations are basically the same, thus reducing the direction dependence of mechanical properties. It can be seen from Fig 3 that the error range of the three parallel experiments is small, the experimental accuracy is high, and the experimental accuracy is high. Fig 4 is the stress-strain curve of the sample in the X direction and the Y direction. It can be seen from the diagram that the numerical error of the yield strength measured by the three parallel tests is small, showing significant strain hardening characteristics.

### 3.3. Impact performance analysis of laser-TIG arc hybrid wire additive manufacturing wall

**Fig 5:**
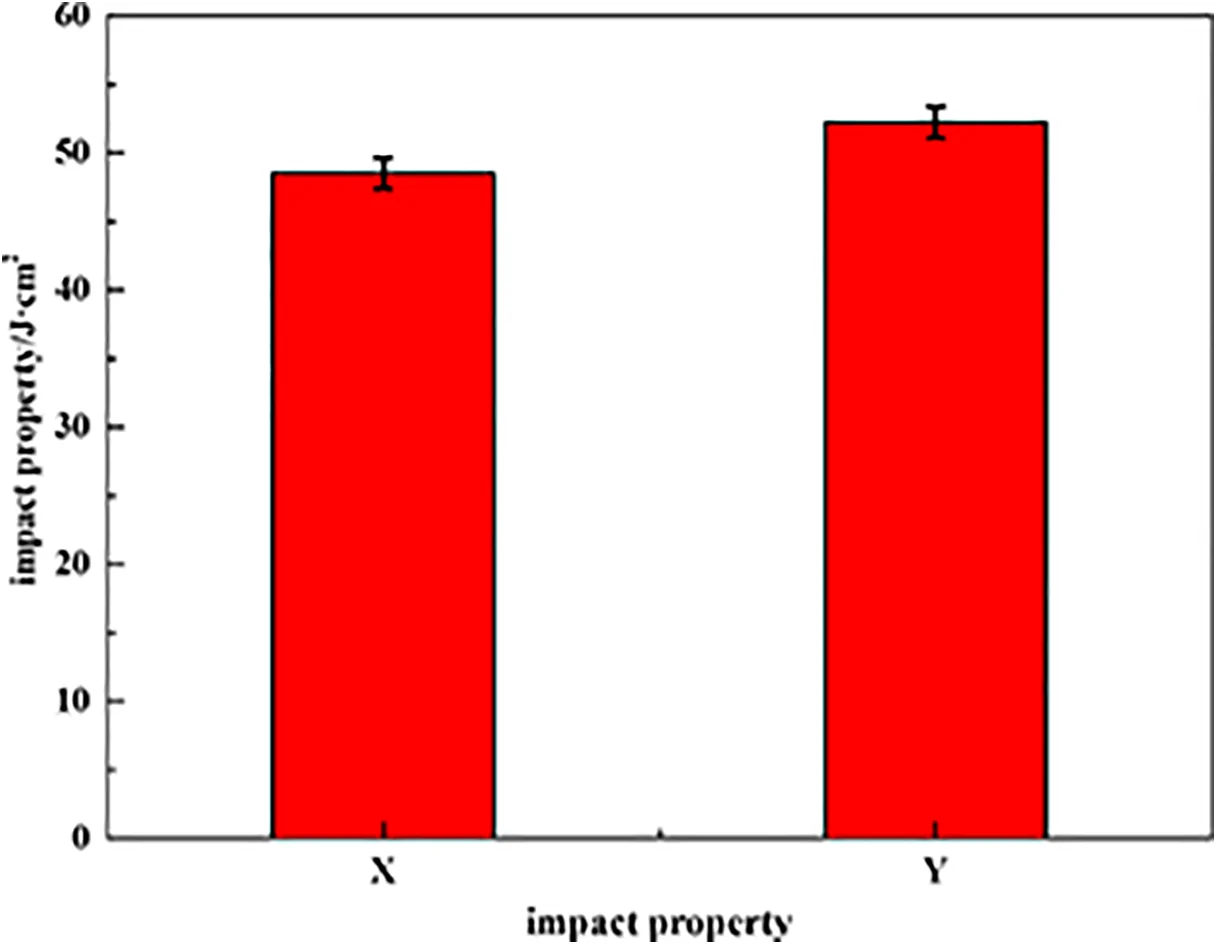
Impact properties of additive samples.

When the laser power is 1100W, the impact energy of the additive sample is measured at room temperature (25^o^C) in both X and Y directions, and the results are shown in Fig 5. It can be seen from Fig 5 that the impact energy of the additive sample at room temperature (25^o^C) in two directions is 48.5 J·cm^−^² (X direction) and 52.2 J·cm^−^² (Y direction), respectively, which meet the requirements of CCS material welding and specification 2023. According to Fig 5, the relative error of the three parallel experiments is small, and the experimental results have high experimental accuracy.The impact toughness of the additive specimen is low. The main reason is that TC4 titanium alloy is a dual-phase titanium alloy. In the process of additive solidification, the grain boundary *β* phase precipitated at the boundary of the acicular *α* phase becomes a weak phase during the impact process, which makes the intergranular fracture occur during the impact process, and the energy needed to be absorbed during the test is small. The results are consistent with those of C. Fu [20].

### 3.4. Microhardness analysis of laser-TIG arc hybrid wire additive manufacturing wall

**Fig 6:**
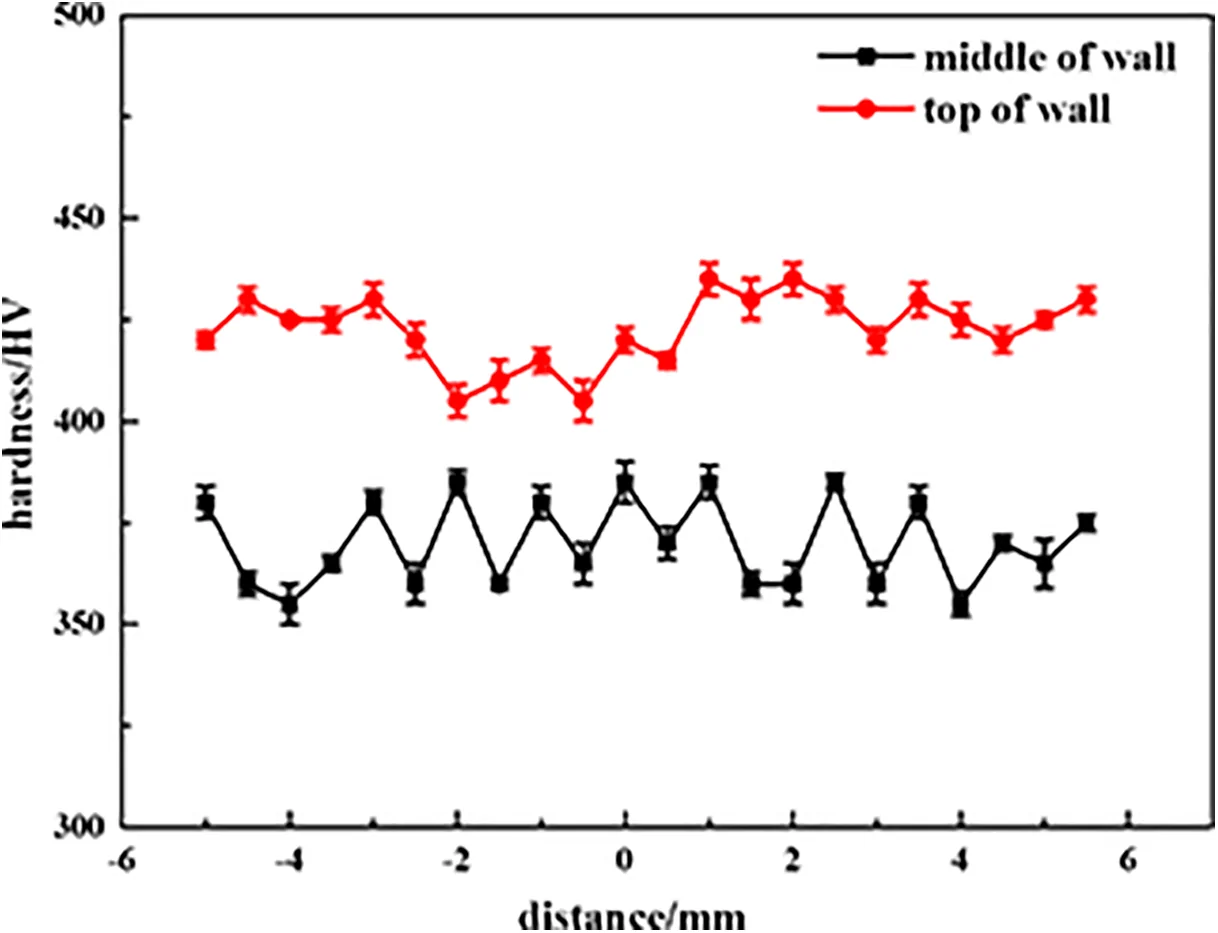
The hardness distribution diagram of the center distance of the wall accumulation layer.

When the laser power is 1100W, the microhardness of the deposit layer at the top and middle of the wall is detected respectively. The hardness distribution of the deposit layer of the single-channel multi-layer forming wall is shown in Fig 6. From the diagram, it can be seen that the microhardness at the top of the wall is higher than that at the middle end of the wall. Due to the fast heat dissipation and cooling rate at the top of the wall, the primary *α* phase and martensite *α* phase are refined and the orientation is dispersed, and the hardness is higher than that of the middle accumulation layer. The hardness fluctuation is due to the difference in microstructure caused by the different heat input between the accumulation layer and the remelting zone. The accumulation layer is mainly composed of *α* and *α* + *β*, while the interlayer remelting zone is mainly composed of lamellar *α* phase, and the lamellar is thicker and the size is larger. Due to the fast heat dissipation and cooling rate at the top of the wall, the primary *α* phase and martensite α phase are refined and the orientation is dispersed, and the hardness is higher than that of the middle accumulation layer. The results are consistent with those of C. Wang [21]. According to Fig 6, the relative error of the three parallel experimental tests is small, and the experimental results have a small error with the average value, so the experimental results have high experimental accuracy.

## 4. Conclusion

In this thesis, the high-quality forming of TC4 titanium alloy welding wire was successfully realized by laser-TIG arc additive manufacturing technology. The main conclusions are as follows: (1) With the increase of laser power, the average layer width of the formed part increases from 7.1 mm to 12.1 mm, an increase of 59%; the average layer height decreased from 1.95 mm to 1.56 mm, a decrease of 20%. (2) When the laser power is 1100 W, the transverse tensile strength is 896 MPa, the longitudinal tensile strength is 883 MPa, and the difference between transverse and longitudinal tensile strength is 13 MPa. The elongation after transverse fracture is 9.31%, the elongation after longitudinal fracture is 10.23%, and the difference of elongation after transverse and longitudinal fracture is 0.92%. (3) The impact energy of the additive samples at room temperature (25°C) in two directions is 48.5 J·cm^−^² (X direction) and 52.2K J·cm^−^² (Y direction), respectively, and the microhardness of the top of the wall is higher than that of the middle end of the wall. This study provides an important experimental basis for the engineering application of laser-TIG arc additive manufacturing technology of TC4 titanium alloy.

## Supporting information

S1 File The dataset underlying the findings presented in this manuscript is provided as a separate file. The data include all relevant experimental measurements, numerical values, and metadata necessary to reproduce the analyses, fiqures, and conclusions reported in the study. Detailed variable definitions and data structure are described within the file. The manuscript files contain everything necessary to replicate the results of our study. All data are in the manuscript and/or supporting information files.
(XLSX)
