# Supplementary material for: Study on microstructure and properties of titanium alloy wall fabricated by laser-TIG arc
Source: PLoS One. 2026 Sep 15;21(9):e0355487. doi: 10.1371/journal.pone.0355487 (PMC13577510; doi:10.1371/journal.pone.0355487)
Supplement: Supplementary file 1 [file pone.0355487.s002.pdf]

PLOS One

Study on microstructure and properties of titanium alloy wall fabricated by laser-TIG arc

--Manuscript Draft--

**Manuscript Number:**	PONE-D-26-14242
**Article Type:**	Research Article
**Full Title:**	Study on microstructure and properties of titanium alloy wall fabricated by laser-TIG arc
**Short Title:**	Study on microstructure and properties of titanium alloy wall fabricated by laser-TIG arc
**Corresponding Author:**	yongchun guo<br>Xi'an Aerospace Propulsion Institute<br>xian, shanxi CHINA
**Keywords:**	laser-arc composite additive; mechanical propertie; TC4 titanium alloy; walls
**Abstract:**	In order to study the influence of heat input on the macroscopic morphology, microstructure and mechanical properties of the wall, TC4 titanium alloy was fabricated by laser-TIG arc hybrid heat source. The results show that: (1) When the laser power is 0W, 400W, 800W and 1600W, with the increase of laser power, the average layer width of the formed part increases from 7.1mm to 12.1mm, an increase of 59%. The average layer height decreased from 1.95 mm to 1.56 mm, a decrease of 20%. (2) When the laser power is 1100W, the transverse tensile strength is 896Mpa, the longitudinal tensile strength is 883Mpa, and the difference between transverse and longitudinal tensile strength is 13Mpa. The elongation after transverse fracture is 9.31%, the elongation after longitudinal fracture is 10.23%, and the difference of elongation after transverse and longitudinal fracture is 0.92%. (3) The impact energy of the additive samples at room temperature (25°C) in two directions is 48.5KJ/m2 (X direction) and 52.2KJ/m2 (Y direction), respectively, and the microhardness at the top of the wall is higher than that at the middle of the wall.
**Order of Authors:**	yongchun guo
	yongchun guo
	S Wang
**Additional Information:**	
**Question**	**Response**
**Financial Disclosure**<br><br>Enter a financial disclosure statement that describes the sources of funding for the work included in this submission. Review the submission guidelines for detailed requirements. View published research articles from *PLOS ONE* for specific examples.<br><br>This statement is required for submission and **will appear in the published article** if the submission is accepted. Please make sure it is accurate.	The author(s) received no specific funding for this work.

Powered by Editorial Manager® and ProduXion Manager® from Aries Systems Corporation

**Funded studies**<br>Enter a statement with the following details:<br>• Initials of the authors who received each award<br>• Grant numbers awarded to each author<br>• The full name of each funder<br>• URL of each funder website<br>• Did the sponsors or funders play any role in the study design, data collection and analysis, decision to publish, or preparation of the manuscript?<br><br>Did you receive funding for this work?	
**Competing Interests**<br><br>Use the instructions below to enter a competing interest statement for this submission. On behalf of all authors, disclose any competing interests that could be perceived to bias this work—acknowledging all financial support and any other relevant financial or non-financial competing interests.<br><br>This statement is required for submission and **will appear in the published article** if the submission is accepted. Please make sure it is accurate and that any funding sources listed in your Funding Information later in the submission form are also declared in your Financial Disclosure statement.<br><br>View published research articles from *PLOS ONE* for specific examples.	no

**NO authors have competing interests**<br><br>Enter: *The authors have declared that no competing interests exist.*<br><br>**Authors with competing interests**<br><br>Enter competing interest details beginning with this statement:<br><br>*I have read the journal's policy and the authors of this manuscript have the following competing interests: [insert competing interests here]*<br><br>* typeset	
**Ethics Statement**<br><br>Enter an ethics statement for this submission. This statement is required if the study involved:<br><br>• Human participants<br>• Human specimens or tissue<br>• Vertebrate animals or cephalopods<br>• Vertebrate embryos or tissues<br>• Field research<br><br>Write "N/A" if the submission does not require an ethics statement.<br><br>General guidance is provided below. Consult the submission guidelines for detailed instructions. **Make sure that all information entered here is included in the Methods section of the manuscript.**	no

**Format for specific study types**<br><br>**Human Subject Research (involving human participants and/or tissue)**<br>• Give the name of the institutional review board or ethics committee that approved the study<br>• Include the approval number and/or a statement indicating approval of this research<br>• Indicate the form of consent obtained (written/oral) or the reason that consent was not obtained (e.g. the data were analyzed anonymously)<br><br>**Animal Research (involving vertebrate animals, embryos or tissues)**<br>• Provide the name of the Institutional Animal Care and Use Committee (IACUC) or other relevant ethics board that reviewed the study protocol, and indicate whether they approved this research or granted a formal waiver of ethical approval<br>• Include an approval number if one was obtained<br>• If the study involved *non-human primates*, add *additional details* about animal welfare and steps taken to ameliorate suffering<br>• If anesthesia, euthanasia, or any kind of animal sacrifice is part of the study, include briefly which substances and/or methods were applied<br><br>**Field Research**<br><br>Include the following details if this study involves the collection of plant, animal, or other materials from a natural setting:<br>• Field permit number<br>• Name of the institution or relevant body that granted permission	
**Data Availability**<br><br>Authors are required to make all data underlying the findings described fully available, without restriction, and from the time of publication. PLOS allows rare exceptions to address legal and ethical concerns. See the PLOS Data Policy and FAQ for detailed information.	No - some restrictions will apply

Powered by Editorial Manager® and ProduXion Manager® from Aries Systems Corporation

A Data Availability Statement describing where the data can be found is required at submission. Your answers to this question constitute the Data Availability Statement and **will be published in the article**, if accepted.<br><br>**Important:** Stating 'data available on request from the author' is not sufficient. If your data are only available upon request, select 'No' for the first question and explain your exceptional situation in the text box.<br><br>Do the authors confirm that all data underlying the findings described in their manuscript are fully available without restriction?	
**Describe where the data may be found in full sentences. If you are copying our sample text, replace any instances of XXX with the appropriate details.**<br><br>• If the data are **held or will be held in a public repository**, include URLs, accession numbers or DOIs. If this information will only be available after acceptance, indicate this by ticking the box below. For example: *All XXX files are available from the XXX database (accession number(s) XXX, XXX.)*.<br>• If the data are all contained **within the manuscript and/or Supporting Information files**, enter the following: *All relevant data are within the manuscript and its Supporting Information files.*<br>• If neither of these applies but you are able to provide **details of access elsewhere**, with or without limitations, please do so. For example:<br><br>*Data cannot be shared publicly because of [XXX]. Data are available from the XXX Institutional Data Access / Ethics Committee (contact via XXX) for researchers who meet the criteria for access to confidential data.*<br><br>*The data underlying the results presented in the study are available from (include the name of the third party*	no

and contact information or URL).<br>• This text is appropriate if the data are owned by a third party and authors do not have permission to share the data.<br><br>* typeset	
Additional data availability information:	

Powered by Editorial Manager® and ProduXion Manager® from Aries Systems Corporation

Study on microstructure and properties of titanium alloy wall fabricated by laser-TIG arc

YC. Guo [1] , MY Li [1*] and S Wang [2]

[1] Xi'an Aeronautical Polytechnic Institute, Xi'an 710089, China
[2] Northeastern University, Shenyang 110819, China

Abstract – In order to study the influence of heat input on the macroscopic morphology, microstructure and mechanical properties of the wall, TC4 titanium alloy was fabricated by laser-TIG arc hybrid heat source. The results show that: (1) When the laser power is 0W, 400W, 800W and 1600W, with the increase of laser power, the average layer width of the formed part increases from 7.1mm to 12.1mm, an increase of 59%. The average layer height decreased from 1.95 mm to 1.56 mm, a decrease of 20%. (2) When the laser power is 1100W, the transverse tensile strength is 896Mpa, the longitudinal tensile strength is 883Mpa, and the difference between transverse and longitudinal tensile strength is 13Mpa. The elongation after transverse fracture is 9.31%, the elongation after longitudinal fracture is 10.23%, and the difference of elongation after transverse and longitudinal fracture is 0.92%. (3) The impact energy of the additive samples at room temperature (25°C) in two directions is 48.5KJ/m^2 (X direction) and 52.2KJ/m^2 (Y direction), respectively, and the microhardness at the top of the wall is higher than that at the middle of the wall.

Keywords: laser-arc composite additive / mechanical propertie / TC4 titanium alloy / walls

1. Introduction

Titanium alloy has a series of excellent characteristics such as high specific strength, excellent corrosion resistance, good low temperature performance, low elastic modulus and low thermal conductivity. With the rapid development of aviation and other industries, the requirements for titanium alloy performance indicators are becoming more and more stringent. In order to meet the use standards of key structural parts such as advanced aero-engines, the development of efficient preparation processes for titanium alloys has become one of the core research hotspots in the field of materials for a long time now and in the future[1-8].

Many scholars at home and abroad have conducted in-depth research on the process problems and organizational performance optimization of additive manufacturing technology. The research of Tsai[9] shows that the reciprocating scanning makes the temperature field of the component change obviously, which is beneficial to the transformation of the equiaxed crystal, and because the formed equiaxed crystal cannot be completely remelted when the next pass is deposited, the microstructure of the reciprocating scanning shows the morphology of alternating growth of columnar crystal and equiaxed crystal. The study of Akerfeldt *et al.*[10] shows that the coarse columnarβgrains grown along the additive direction are one of the main factors affecting the anisotropy of the additive components. Edwards *et al.*[11] analyzed the effect of pores on the overall mechanical properties of the samples fabricated by laser deposition. Razavi *et al.*[12] used TSLM technology to prepare Ti-6Al-4V titanium alloy, and found that the surface quality of the workpiece produced by this method is high, and the defects are all on the surface of the workpiece. Brand *et al.*[13] carried out heat treatment on the subsequent laser additive manufacturing TC4 titanium alloy samples, and found that the mechanical properties of the samples were improved and the strength was improved. Baufeld *et al.*[14] found that the upper limit of fatigue strength of titanium alloys fabricated by additive manufacturing was increased by heat treatment. On this basis, Vrancken *et al.*[15] increased the temperature of heat treatment. When the transition temperature of a certain phase is reached, the mechanical properties of titanium alloys can be significantly improved. Zhang *et al.*[16] take a special temperature of titanium alloy annealing treatment, the sample, the material properties of uniform distribution, anisotropy has been significantly improved. Up to now, many scholars at home and abroad have been unable to meet the development needs by using a single additive manufacturing technology. When the laser is used as the heat source, the quality requirements of the wire are high, resulting in relatively high manufacturing costs and low molding quality. When the arc is used as the heat source, the forming quality and mechanical properties of the parts are poor, and the surface roughness is large.

Therefore, in this paper, TC4 titanium alloy, which is widely used in aerospace engineering, is used as the main test material to study the laser-TIG arc hybrid additive manufacturing process and performance control of titanium alloy. Based on single-channel single-layer deposition, the additive manufacturing of single-channel multi-layer thin-wall is carried out. The mechanism and influence law of heat accumulation of the deposition layer are analyzed, and the regulation of process parameters on the forming morphology and deposition efficiency of thin-wall size is realized, so as to solve the problems of difficult forming of titanium alloy and difficult processing of precision structural parts.

2. Material and Methods

In the experiment, TC4 titanium alloy was manufactured by laser-TIG arc hybrid wire additive manufacturing technology, and the TC4 titanium alloy wire with a diameter of 1.2 mm was selected as the filling wire. The hot rolled TC4 titanium alloy sheet is selected as the substrate, and the specific size is 150mm×200mm×20mm. The chemical composition of the material is shown in **Table 1**. The laser-TIG arc hybrid wire additive manufacturing platform includes fiber laser, KUKA robot arm, wire feeder, TIG welding torch, etc. The robot and positioner are used as the motion system. The push-pull wire feeder sends the wire into the laser processing head. The test is carried out in the argon chamber, and the argon flow rate is 15~25 L/min to ensure that the oxygen content of the argon chamber during the additive process is less than 50 ppm, as shown in **Fig. 1**. Before the test, the titanium wire wheel was used to clean the oxide film on the surface of the substrate, and acetone was used to remove oil and dust. The best process window of laser-TIG arc hybrid wire additive manufacturing TC4 titanium alloy wall is shown in **Table 2**.

Table 1 Chemical composition of TC4 titanium alloy wire and plate(mass fraction)

materials	Al	V	H	N	O	Fe	C	Ti
filament	5.5~6.8	3.5~4.5	≤0.015	≤0.05	≤0.02	≤0.03	≤0.01	-
lumber	7.5~9.0	3.7~4.9	≤0.015	≤0.05	≤0.02	≤0.03	≤0.01	-

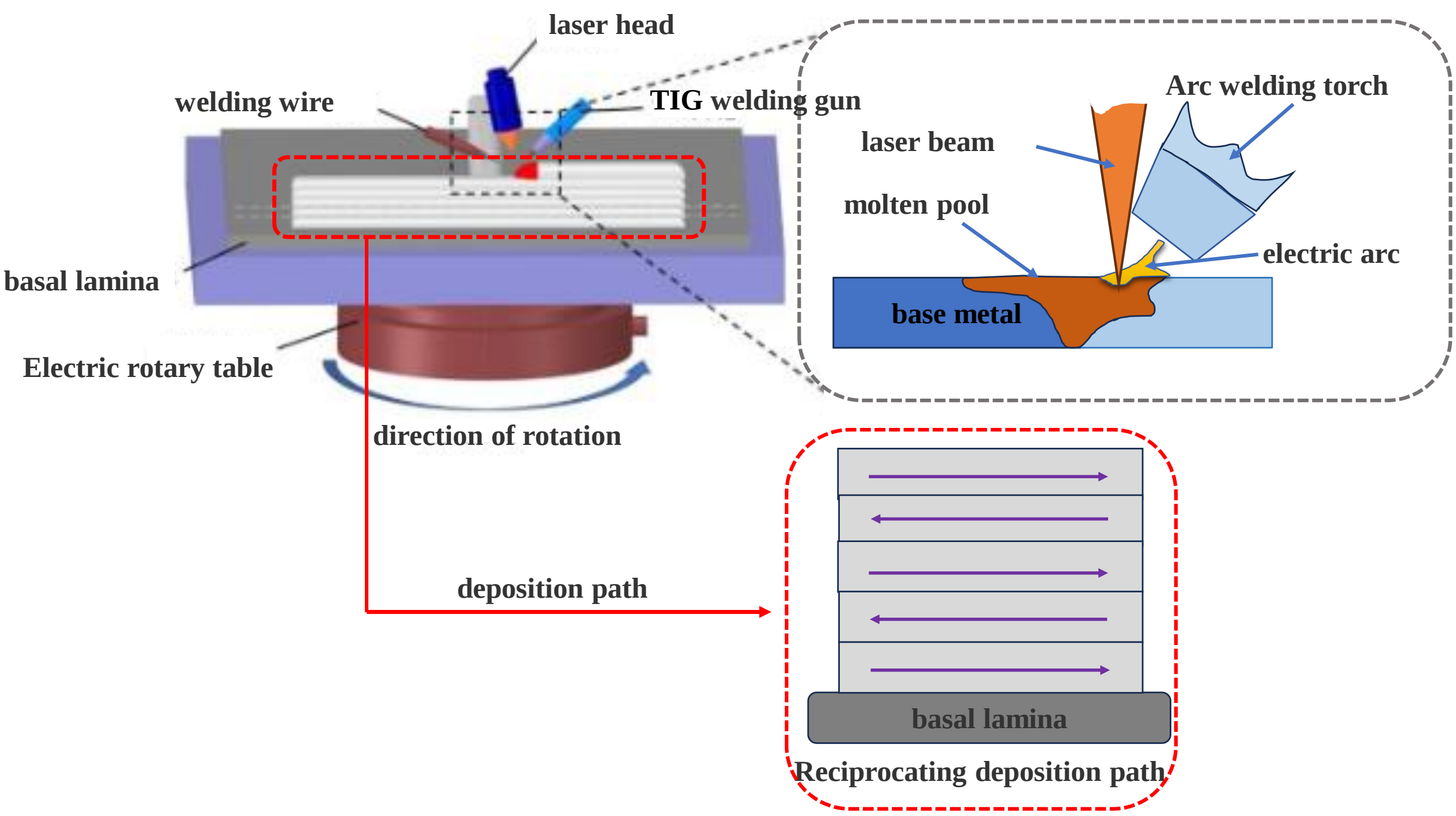

Fig. 1 Schematic diagram of laser-arc hybrid additive manufacturing platform

Table 2 Laser-arc hybrid additive manufacturing TC4 titanium alloy optimal process

parameter	laser power P/W	welding current I/A	arc voltage U/V	wire feed rate v_f/(m.min^{-1})	deposit velocity v_w/(m.min^{-1})	laser-arc distance D/mm
numerical value	1100	156	20	6	0.3	1.5

At the end of the experiment, the standard tensile specimens (parallel section 5 mm) were prepared according to GB/T 228.1-2021 in both vertical and horizontal directions, and the tensile test was carried out by INSTRON5985-250kN material testing machine. The V-shaped impact specimens (10mm×10mm×55mm) were prepared according to GB/T229-2020, and the impact test was carried out at room temperature (25°C) by ZBC2602-B pendulum impact testing machine. The mechanical properties of the additive specimens were systematically studied, as shown in **Fig. 2**.

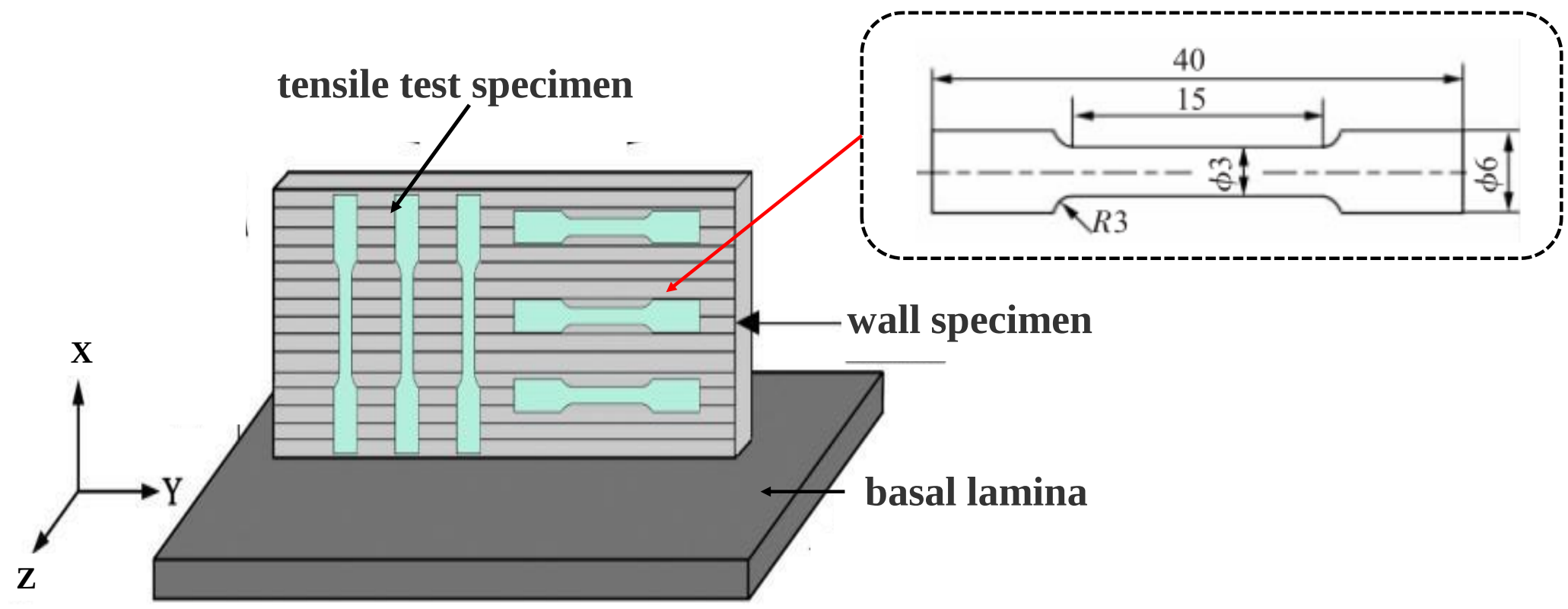

Fig. 2 Sampling diagram

3. Results and Discussion

3.1 Macroscopic morphology analysis of laser-TIG arc hybrid wire additive manufacturing wall

When the laser power is 0W, 400W, 800W and 1600W, the influence of laser power on the average layer width and average layer height of the formed parts is discussed. The results are shown in **Fig. 3** and **Fig. 4**. It can be seen from the figure that when the laser power is 0W, 400W, 800W and 1600W, as the laser power increases, the average layer width of the formed part increases from 7.1mm to 12.1mm, an increase of 59%. The average layer height decreased from 1.95 mm to 1.56 mm, a decrease of 20%. When the laser power is 0W, 400W, 800W and 1600W, as the laser power increases, the heat input gradually increases, and the heat accumulation inside the formed part becomes larger, and the laser shock pool changes the fluidity of the molten pool, resulting in the overflow of the molten pool and the collapse of the molten pool, which in turn affects the average layer width and layer height of the wall of the formed part. Therefore, when the laser power is 0W, 400W, 800W and 1600W, with the increase of laser power, the average layer width of the formed wall increases gradually, and the average layer height decreases gradually.

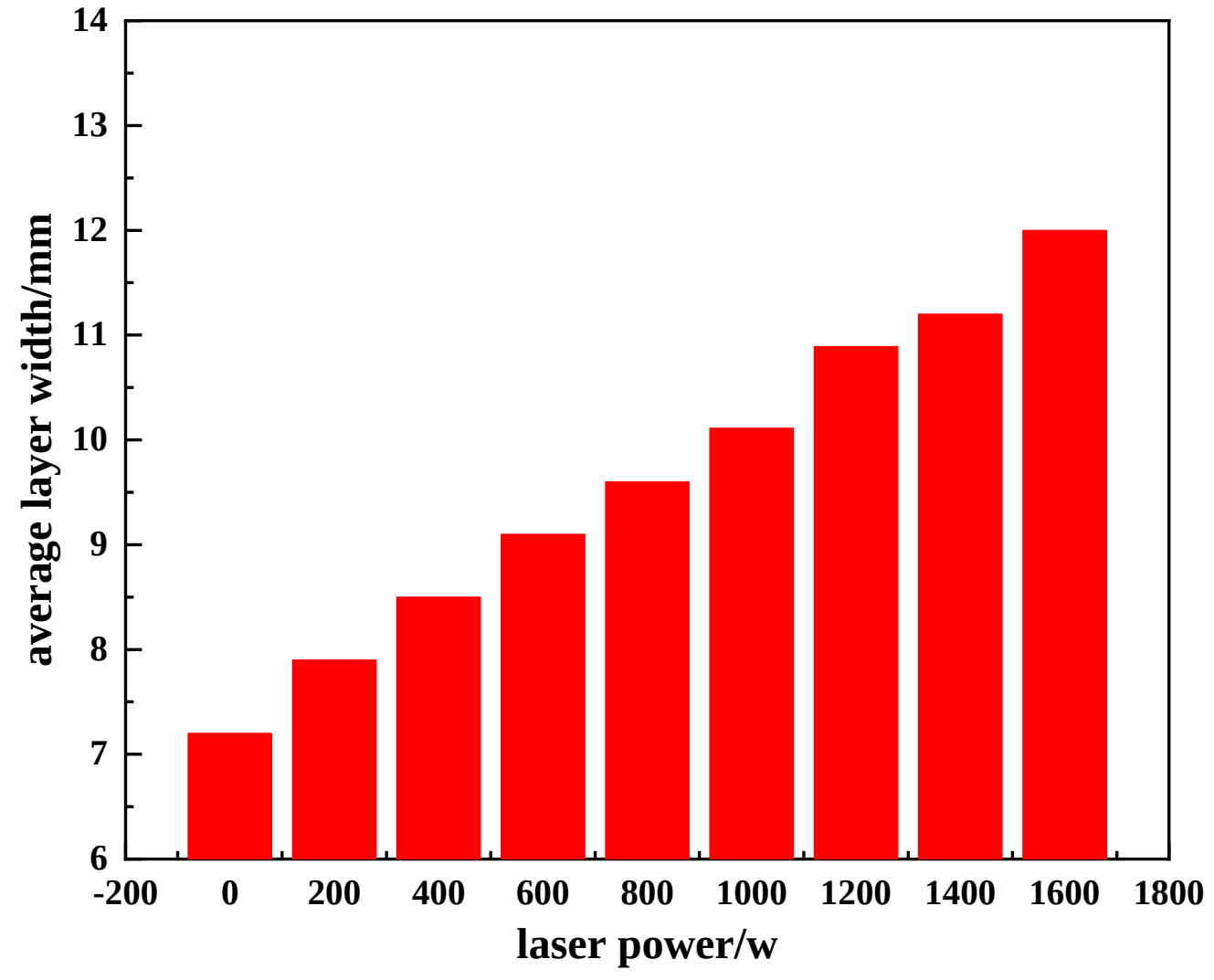

Fig. 3 The effect of laser power on the average layer width

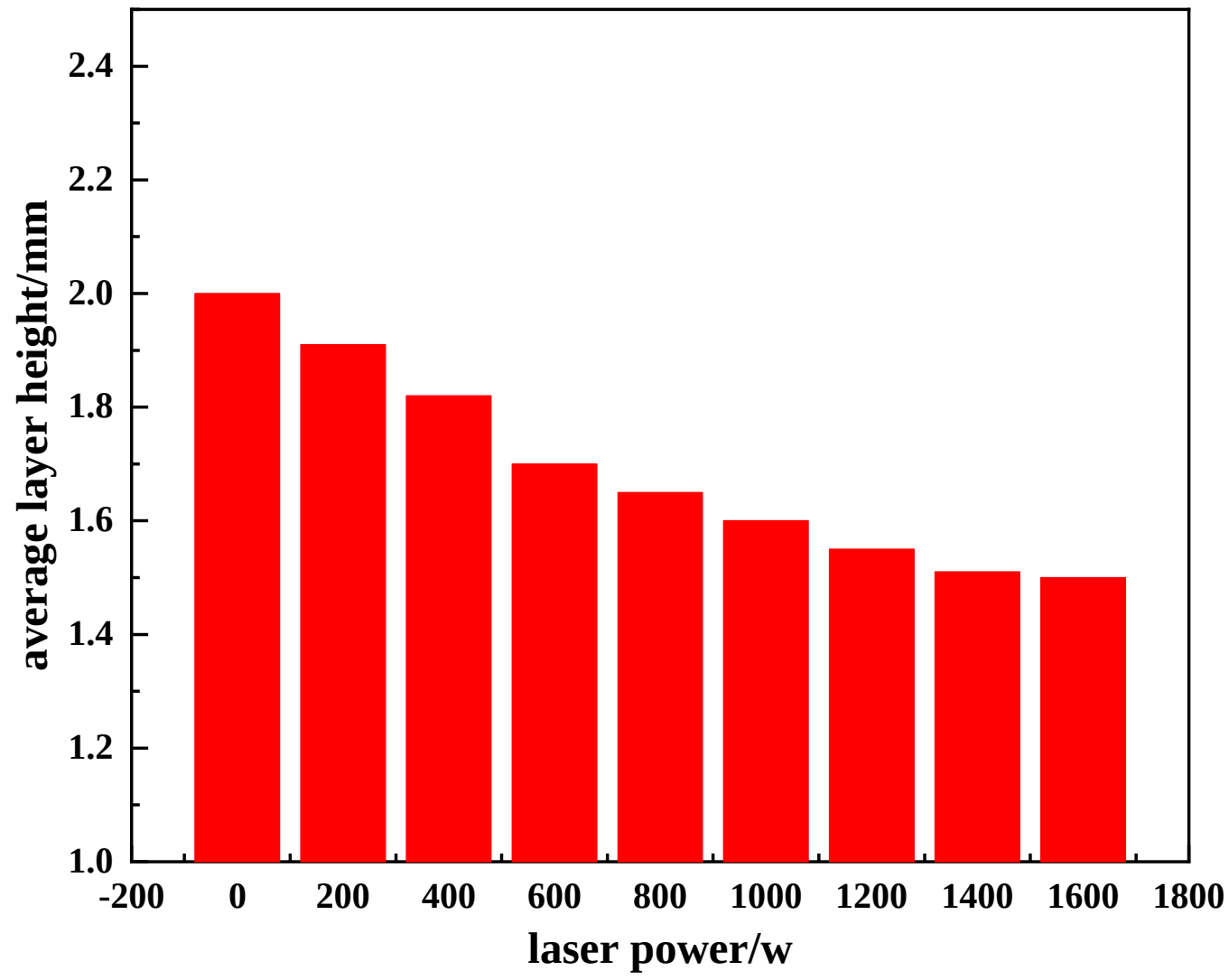

Fig. 4 The effect of laser power on the average layer height

3.2 Analysis of tensile properties of laser-TIG arc hybrid wire additive manufacturing wall

When the laser power is 1100W, the tensile properties of TC4 titanium alloy in both transverse (X) and longitudinal (Y) directions are discussed. The results are shown in **Fig.5** and **Fig.6**. From Figure 5 and Figure 6, it can be seen that when the laser power is 1100W, the transverse tensile strength is 896Mpa, the longitudinal tensile strength is 883Mpa, and the difference between the transverse and longitudinal tensile strength is 13Mpa ; the transverse elongation after fracture is 9.31 %, the longitudinal elongation after fracture is 10.23 %, and the difference between transverse and longitudinal elongation after fracture is 0.92 %, indicating that the anisotropy of tensile properties is small. This low anisotropy is mainly due to the reciprocating deposition method : the interlayer deposition directions are perpendicular to each other, so that the grain structures in different directions are similar, and the grain orientations are basically the same, thus reducing the direction dependence of mechanical properties.

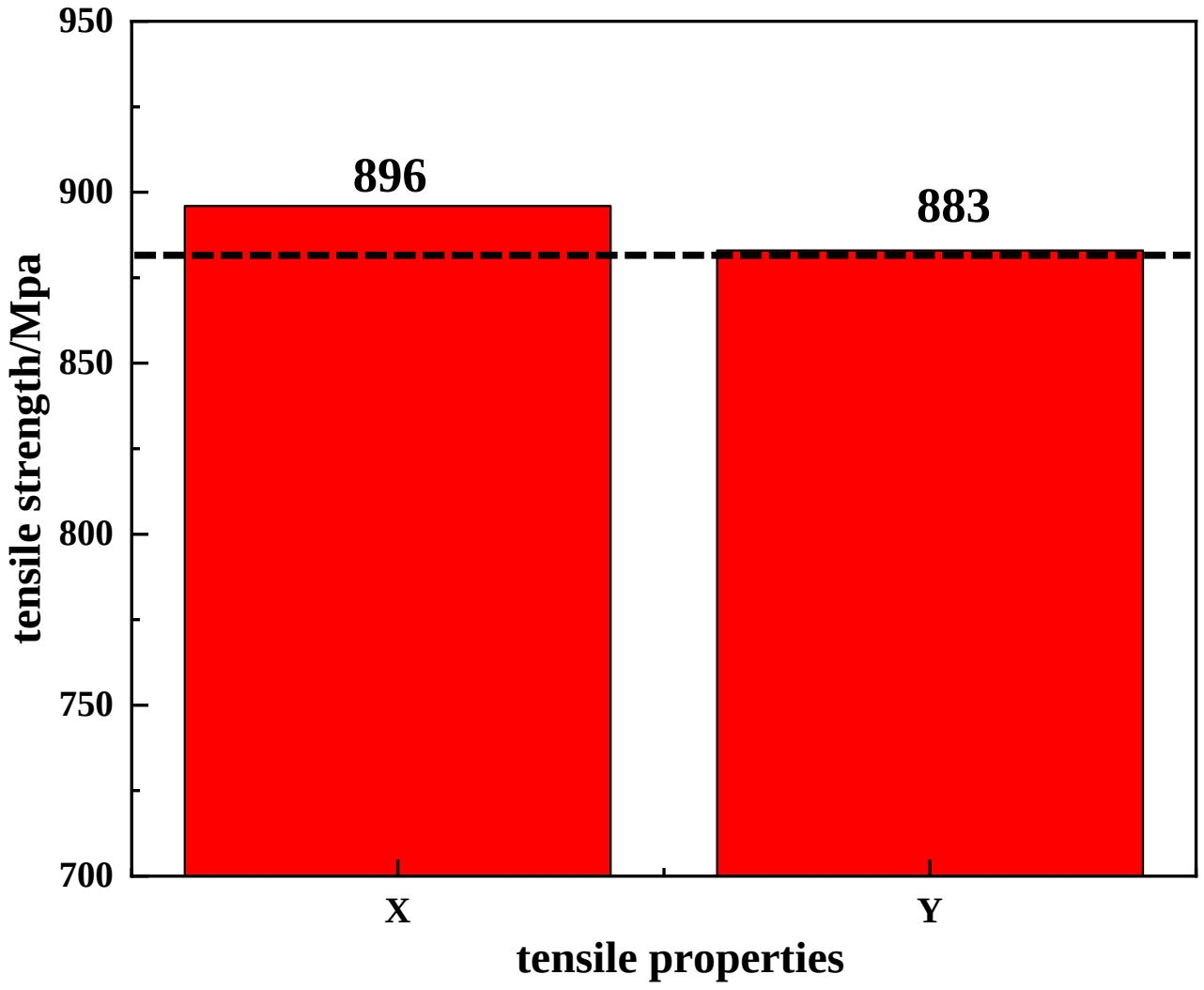

Fig.5 Tensile strength of additive samples

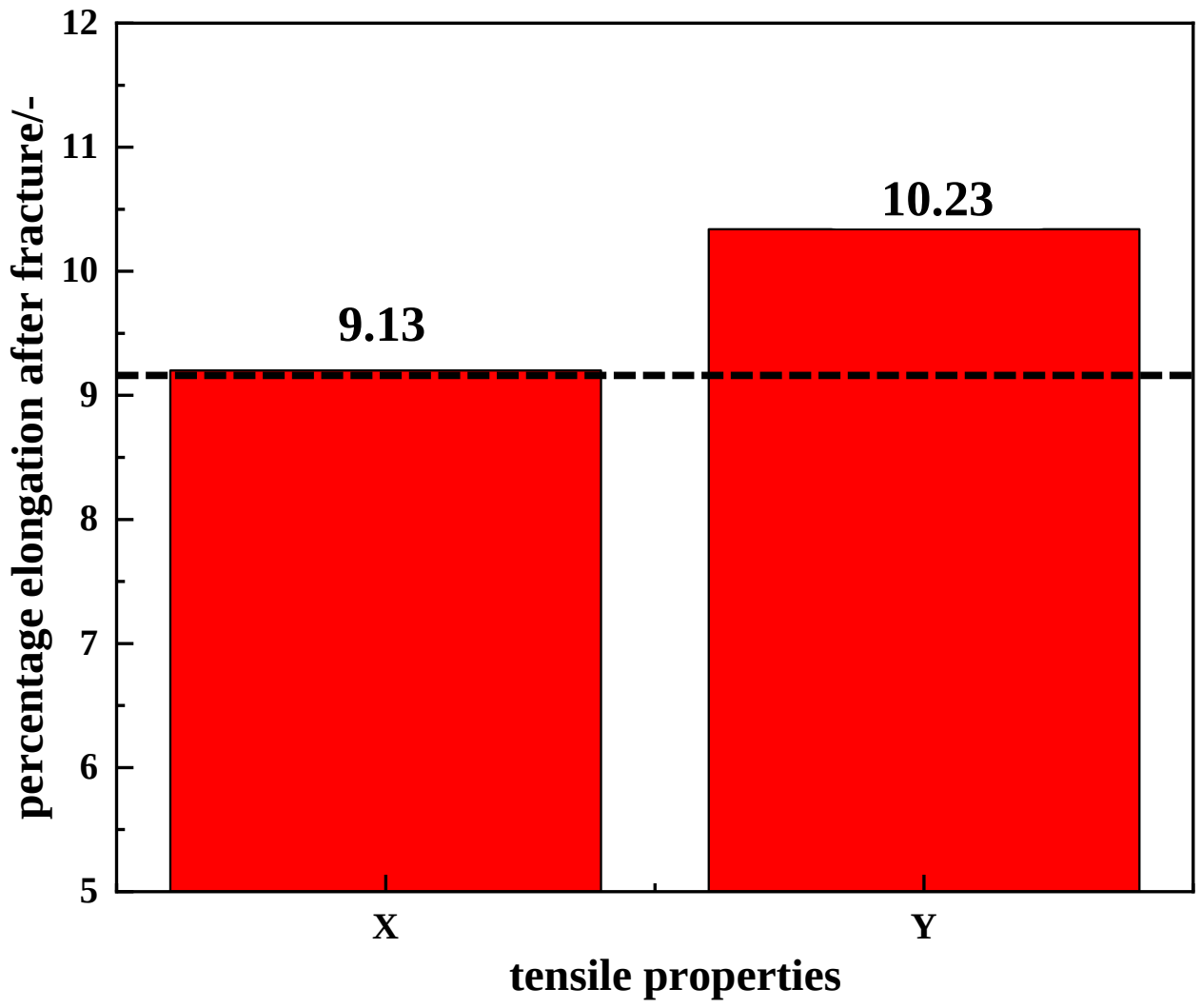

Fig.6 Post-fracture elongation of additive samples

3.3 Impact performance analysis of laser-TIG arc hybrid wire additive manufacturing wall

When the laser power is 1100W, the impact energy of the additive sample is measured at room temperature (25°C) in both X and Y directions, and the results are shown in **Fig.7**. It can be seen from **Fig.7** that the impact energy of the additive sample at room temperature (25°C) in two directions is 48.5 KJ/m^2 (X direction) and 52.2 KJ /m^2 (Y direction), respectively, which meet the requirements of CCS material welding and specification 2023. The impact toughness of the additive specimen is low. The main reason is that TC4 titanium alloy is a dual-phase titanium alloy. In the process of additive solidification, the grain boundary β phase precipitated at the boundary of the acicular α phase becomes a weak phase during the impact process, which makes the intergranular fracture occur during the impact process, and the energy needed to be absorbed during the test is small.

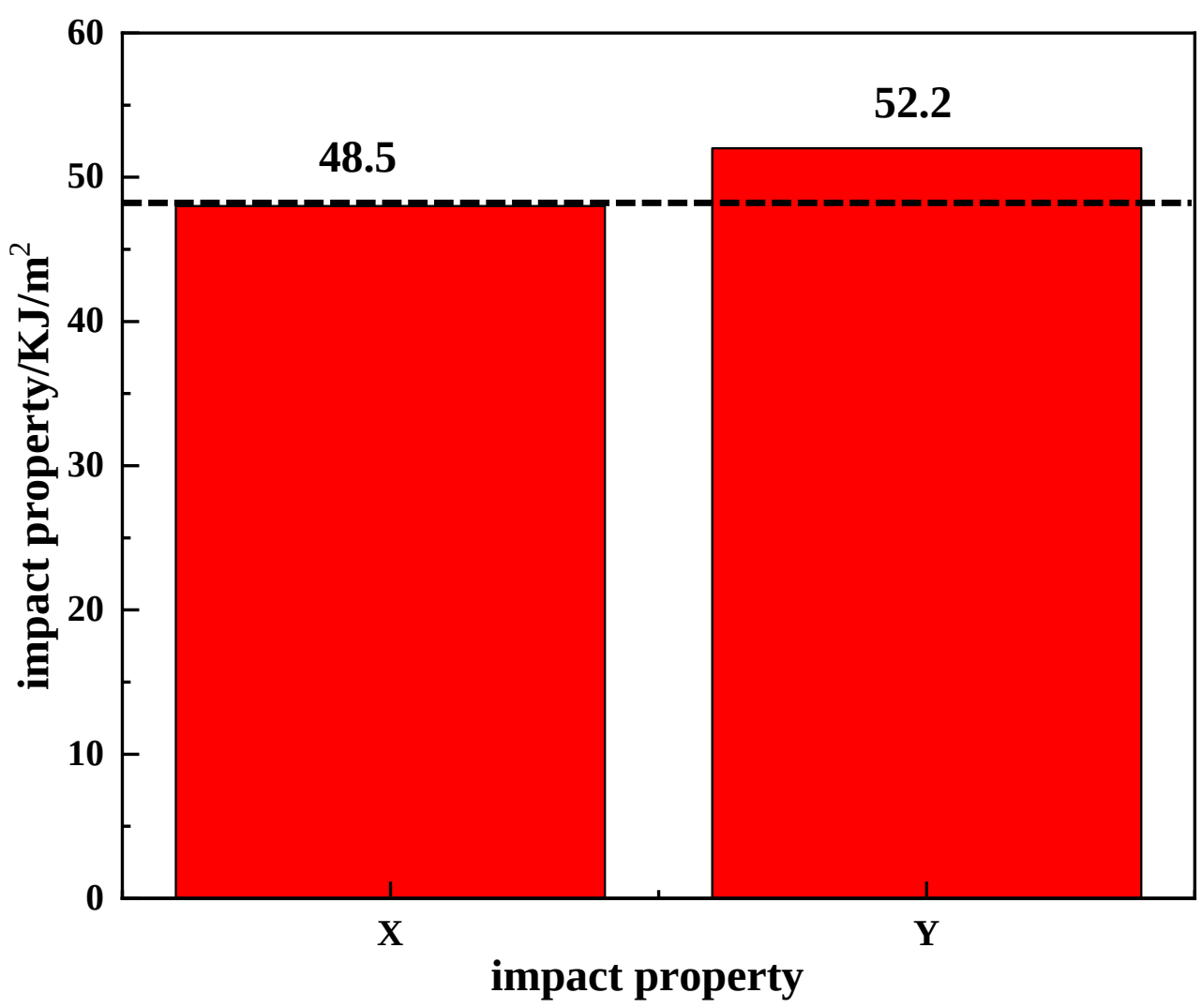

Fig.7 Impact properties of additive samples

3.4 Microhardness analysis of laser-TIG arc hybrid wire additive manufacturing wall

When the laser power is 1100W, the microhardness of the deposit layer at the top and middle of the wall is detected respectively. The hardness distribution of the deposit layer of the single-channel multi-layer forming wall is shown in **Fig.8**. From the diagram, it can be seen that the microhardness at the top of the wall is higher than that at the middle end of the wall. Due to the fast heat dissipation and cooling rate at the top of the wall, the primary *α* phase and martensite *α* phase are refined and the orientation is dispersed, and the hardness is higher than that of the middle accumulation layer. The hardness fluctuation is due to the difference in microstructure caused by the different heat input between the accumulation layer and the remelting zone. The accumulation layer is mainly composed of *α* and *α* + *β*, while the interlayer remelting zone is mainly composed of lamellar *α* phase, and the lamellar is thicker and the size is larger. Due to the fast heat dissipation and cooling rate at the top of the wall, the primary *α* phase and martensite α phase are refined and the orientation is dispersed, and the hardness is higher than that of the middle accumulation layer.

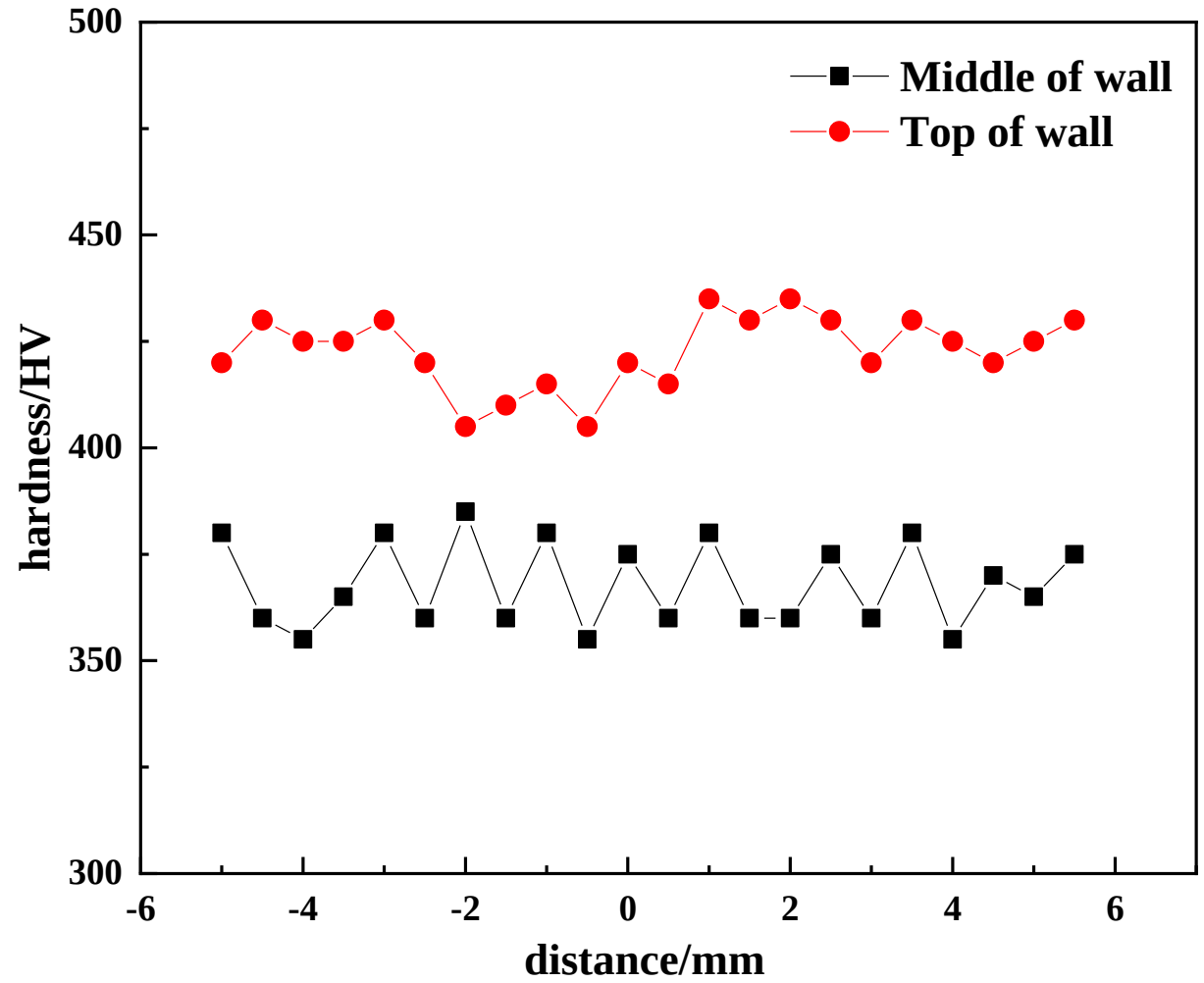

Fig.8 The hardness distribution diagram of the center distance of the wall accumulation layer

4. Conclusion

In this thesis, TC4 titanium alloy, which is widely used in aerospace engineering, is used as the main test material to study the laser-TIG arc hybrid additive manufacturing process and performance control of titanium alloy. The research results are as follows: (1) With the increase of laser power; (2) When the laser power is 1100 W, the transverse tensile strength is 896 Mpa, the longitudinal tensile strength is 883 Mpa, and the difference between transverse and longitudinal tensile strength is 13 Mpa. The elongation after transverse fracture is 9.31%, the elongation after longitudinal fracture is 10.23 %, and the difference of elongation after transverse and longitudinal fracture is 0.92 %. (3) The impact energy of the additive samples in two directions is 48.5KJ/m^2(X direction) and 52.2KJ/m^2(Y direction) respectively, and the microhardness of the top of the wall is higher than that of the middle of the wall.

Acknowledgments

The authors wish to acknowledge the contributions of associates and colleagues from the Xi'an Aeronautical Polytechnic Institute and Northeastern University of China.

Funding

Scientific Research Program Funded by Education Department of Shaanxi Provincial Government (Program No. 24JK0506) and Scientific and Technological Plan Project of Xi'an Science and Technology Bureau (Program No. 2025JC-YBQN-774).

This research was financially supported by the Xi’an Aeronautical Polytechnic Institute (22XHZK-05) and (115020121/76/2023XHQDJ02).

Conflicts of interest

The authors declare no conflict of interest.

Data availability statement

This article has no associated data generated.

Author contribution statement

Conceptualization, YC Guo and MY Li; methodology, MY Li; software, MY Li; writing—original draft preparation, MY Li; writing—review and editing, S Wang; visualization, S Wang; supervision, MY Li. All authors replied to reviewers' comments and revised the final version.

References

[1] H. J. Wang, The high temperature deformation behavior of TC11 titanium alloy and the effect of heat treatment process on the microstructure and properties of the alloy were studied, Central South University, 2010.

[2] S. L. Semiatin, An overview of the thermomechanical processing of α/β titanium alloys: current status and future research opportunities, Metall. Mater. Trans. A. **51**, 2593 (2020). https://link.springer.com/article/10.1007/s11661-020-05625-3

[3] M. Motyka, Martensite formation and decomposition during traditional and AM processing of two phase titanium alloys-an overview, Metals. **3**, 11 (2021). https://www.mdpi.com/2075-4701/11/3/481

[4] P. Yadav, K. K. Saxena, Effect of heat-treatment on microstructure and mechanical properties of Ti alloys: An overview, Materials Today: Proceedings. **26**, 2546 (2020). https://www.sciencedirect.com/science/article/abs/pii/S2214785320312967

[5] L. C. Zhang, L. Y. Chen, L. Wang, Surface modification of titanium and titanium alloys: technologies, developments, and future interests, Adv. Eng. Mater. **5**, 22 (2020). https://advanced.onlinelibrary.wiley.com/doi/10.1002/adem.202070017

[6] T. Guo, The high temperature deformation behavior of TC11 titanium alloy and the effect of heat treatment process on the microstructure and properties of the alloy were studied, China University of Mining & Technology, 2021.

[7] Y. Hao, Y. Huang, K. Zhao, Research on the microstructure and mechanical properties of doubled annealed laser melting deposition TC11 titanium alloy, Opt. Laser. Technol. **5**, 150 (2022). https://www.sciencedirect.com/science/article/abs/pii/S0030399222001402

[8] L. J. Huang, Study on equiaxed treatment and high temperature deformation behavior of TC11 titanium alloy, Harbin Institute of Technology, 2007.

[9] C. J. Tsai, Improved mechanical properties of Ti-6Al-4V alloy by electron beam welding process plus annealing treatments and its microstructural evolution, Mater. Design. **60**, 587 (2014). https://www.sciencedirect.com/science/article/abs/pii/S0261306914003057

[10] P. Akerfeldt, M. L. Antti, R. Pederson, Influence of microstructure on mechanical properties of laser metal wire-deposited Ti-6Al-4V, Mat. Sci. Eng. A-Struct. **674**, 428 (2016). https://www.sciencedirect.com/science/article/abs/pii/S0921509316307924

[11] P. Edwards, M. Ramulu, Fatigue performance evaluation of selective laser melted Ti-6Al-4V, Mat Sci Eng. **598**, 327 (2014). https://www.sciencedirect.com/science/article/abs/pii/S0921509314000720

[12] S. M. Razavi, P. Ferro, F. Berto, Fatigue strength of blunt v-notched sppeciens produced by selective laser melting of Ti-6Al-4V, Theor. Appl. Fract. Mec. **97**, 376 (2017). https://www.sciencedirect.com/science/article/abs/pii/S0167844217302884

[13] E. Brandl, F. Palm, V. Michailov, Mechanical properties of additive manufactured titanium (Ti-6Al-4V) blocks deposited by a solid-state laser and wire, Mater. Design. **32**, 10 (2011). https://www.sciencedirect.com/science/article/abs/pii/S0261306911004705

[14] B. Baufeld, E. Brandl. Wire based additive layer manufacturing: Comparison of microstructure and mechanical properties of Ti-6Al-4V components fabricated by laser-beam deposition and shaped metal deposition, J. Mater. Process. Tech. **211**, 6 (2011). https://www.sciencedirect.com/science/article/abs/pii/S0924013611000306

[15] B. Vrancken, L. Thijs, J. P. Kruth, Heat treatment of Ti6Al4V produced by Selective Laser Melting: Microstructure and mechanical properties, J. Alloy. Compd. **541**, 177 (2012). https://www.sciencedirect.com/science/article/abs/pii/S0925838812011826

[16] Q. Zhang, J. Chen, Zhao Z, Microstructure and anisotropic tensile behavior of laser additive manufactured TC21 titanium alloy, Mat. Sci. Eng. A-Struct. **673**, 204 (2016). https://www.sciencedirect.com/science/article/abs/pii/S092150931630795X

 Click here to access/download;Figure;Figure files.docx

List of Figure

Fig. 1 Schematic diagram of laser-arc hybrid additive manufacturing platform

Fig. 2 Sampling diagram

Fig. 3 The effect of laser power on the average layer width

Fig. 4 The effect of laser power on the average layer height

Fig. 5 Tensile strength of additive samples

Fig. 6 Post-fracture elongation of additive samples

Fig. 7 Impact properties of additive samples

Fig. 8 The hardness distribution diagram of the center distance of the wall accumulation layer

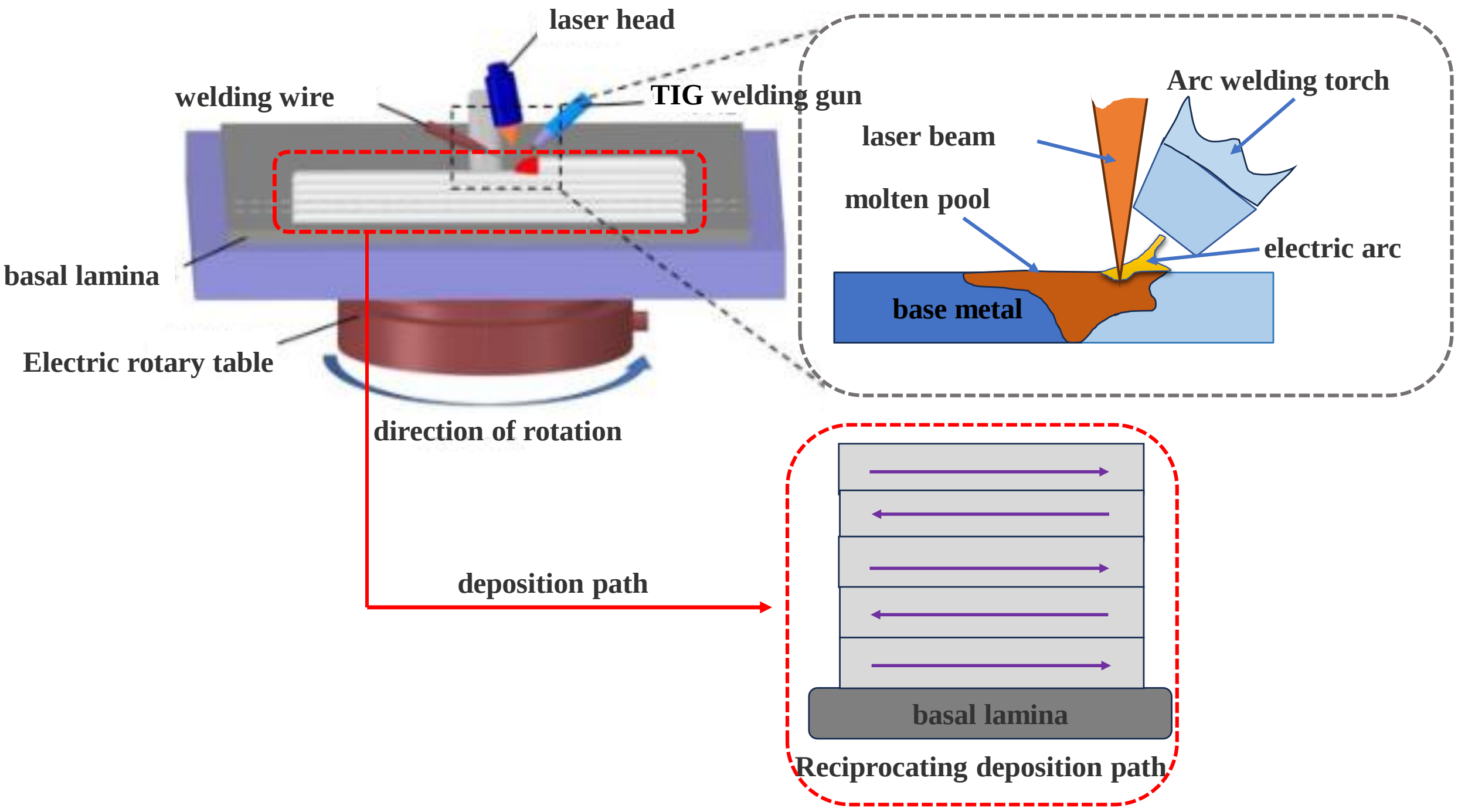

Fig. 1 Schematic diagram of laser-arc hybrid additive manufacturing platform

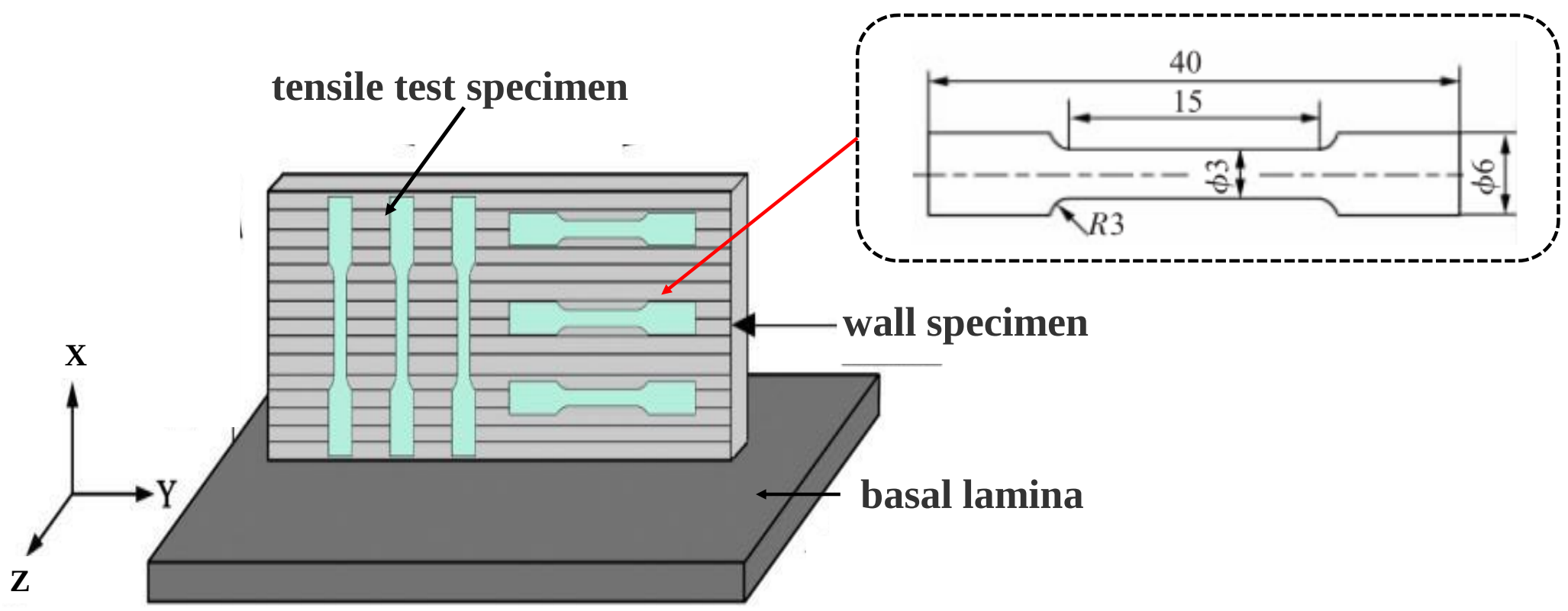

Fig. 2 Sampling diagram

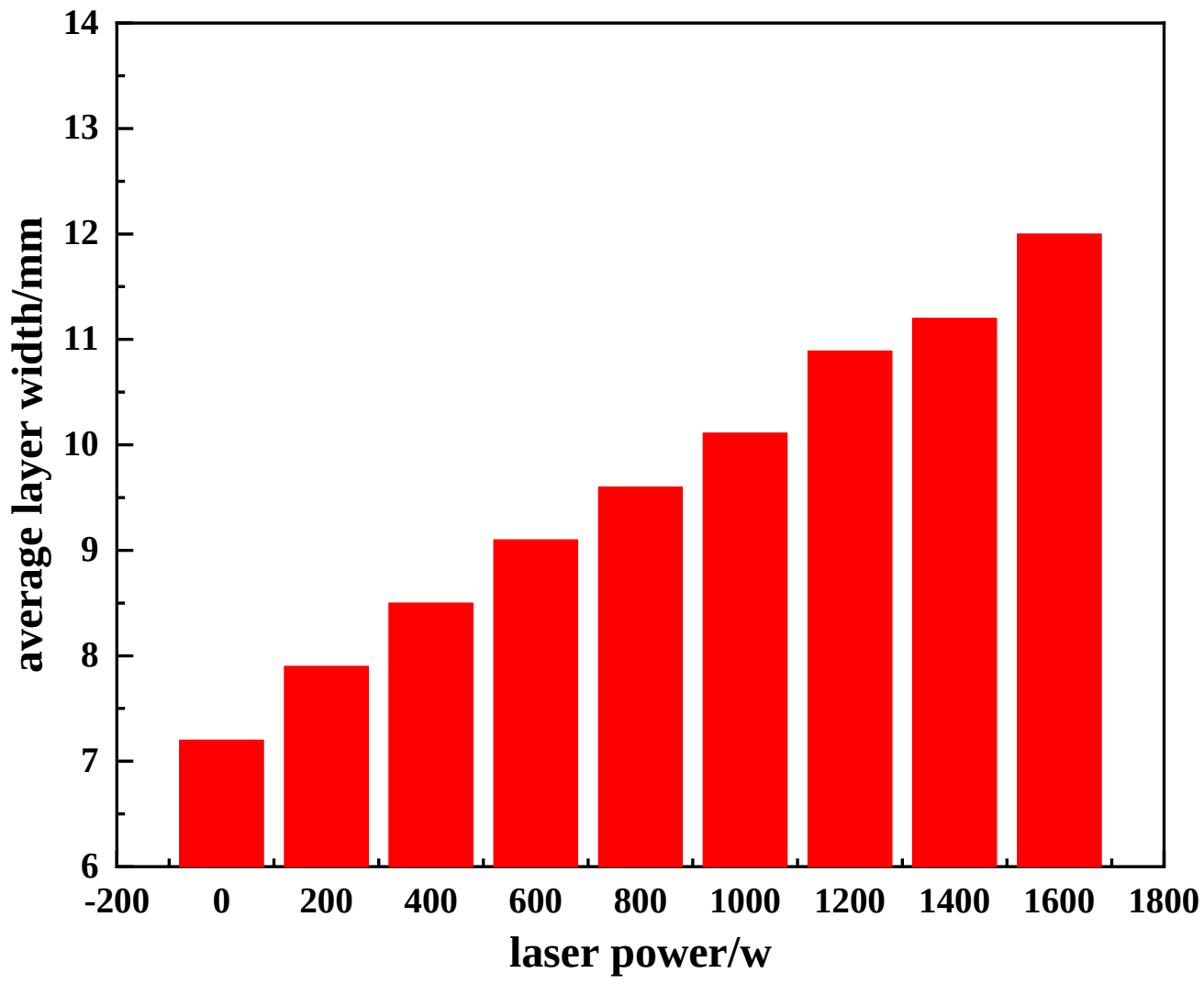

Fig. 3 The effect of laser power on the average layer width

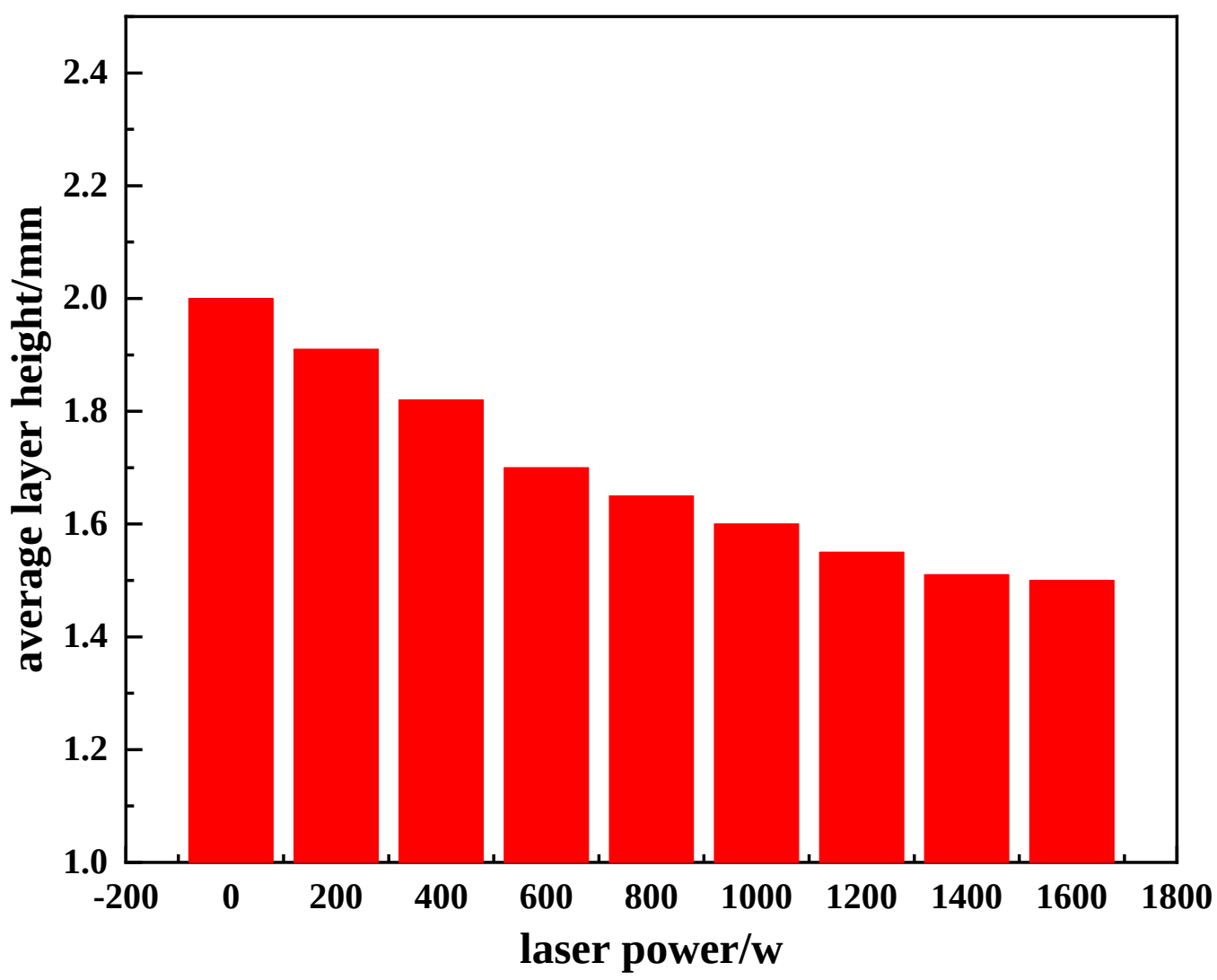

Fig. 4 The effect of laser power on the average layer height

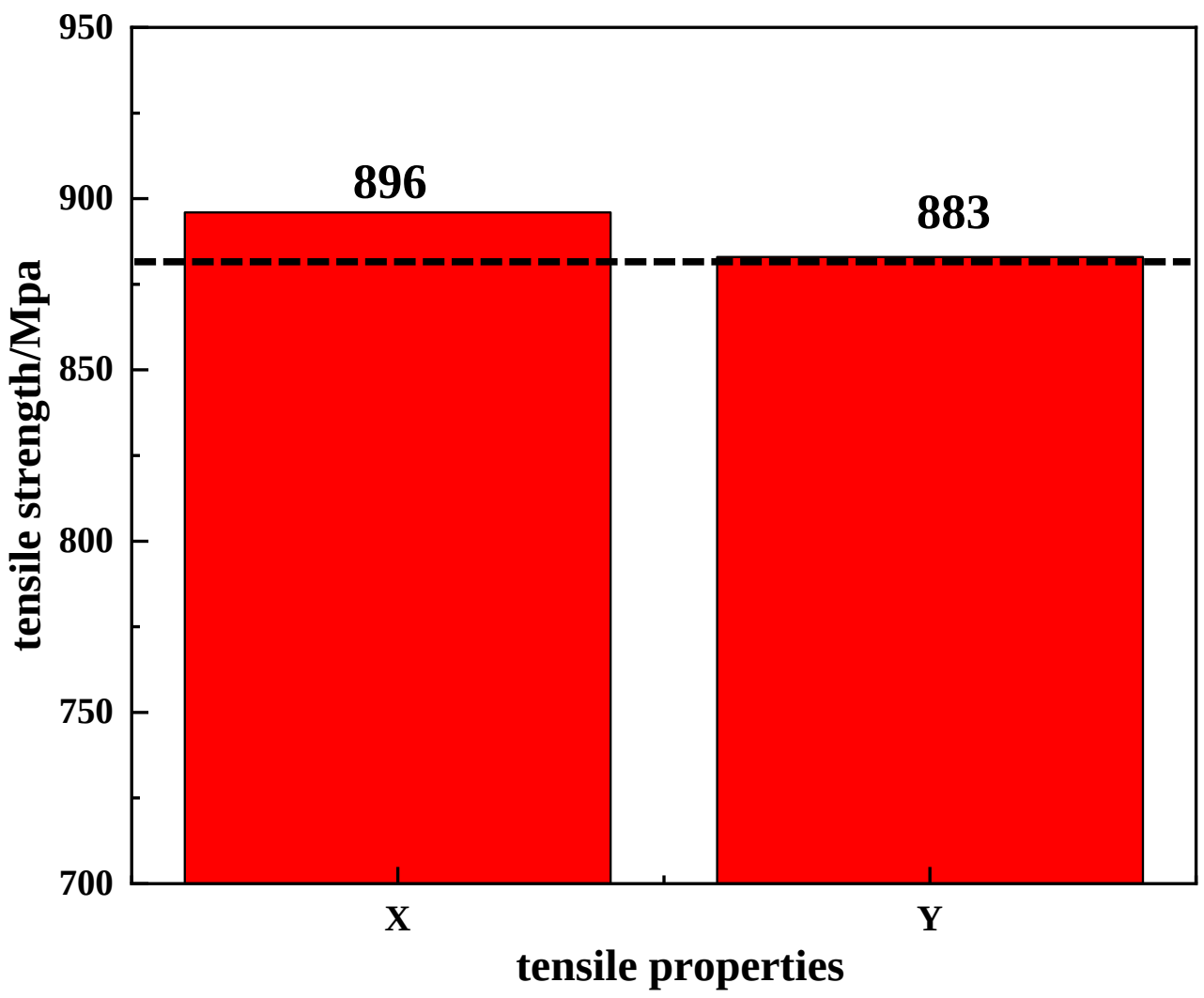

Fig. 5 Tensile strength of additive samples

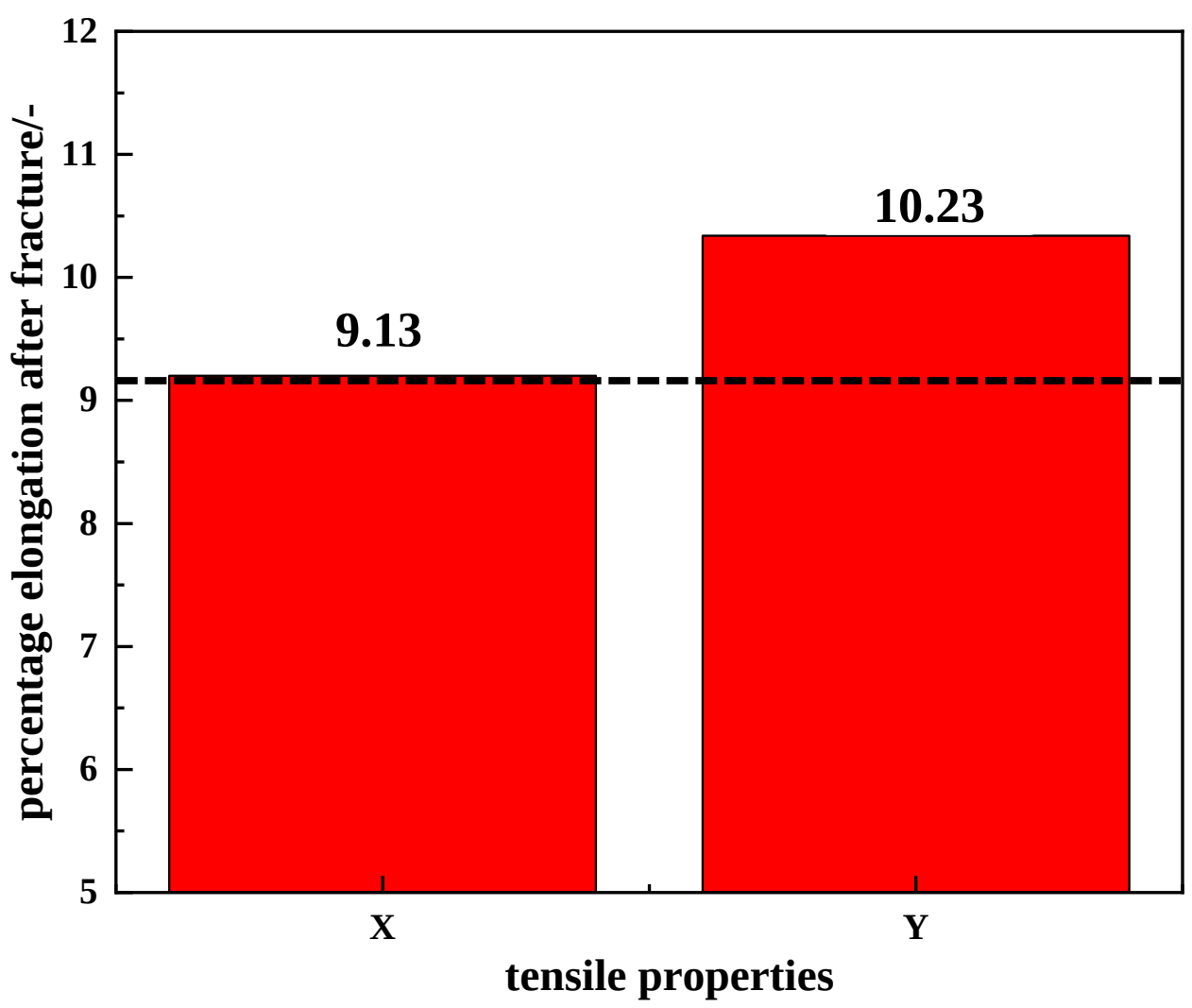

Fig. 6 Post-fracture elongation of additive samples

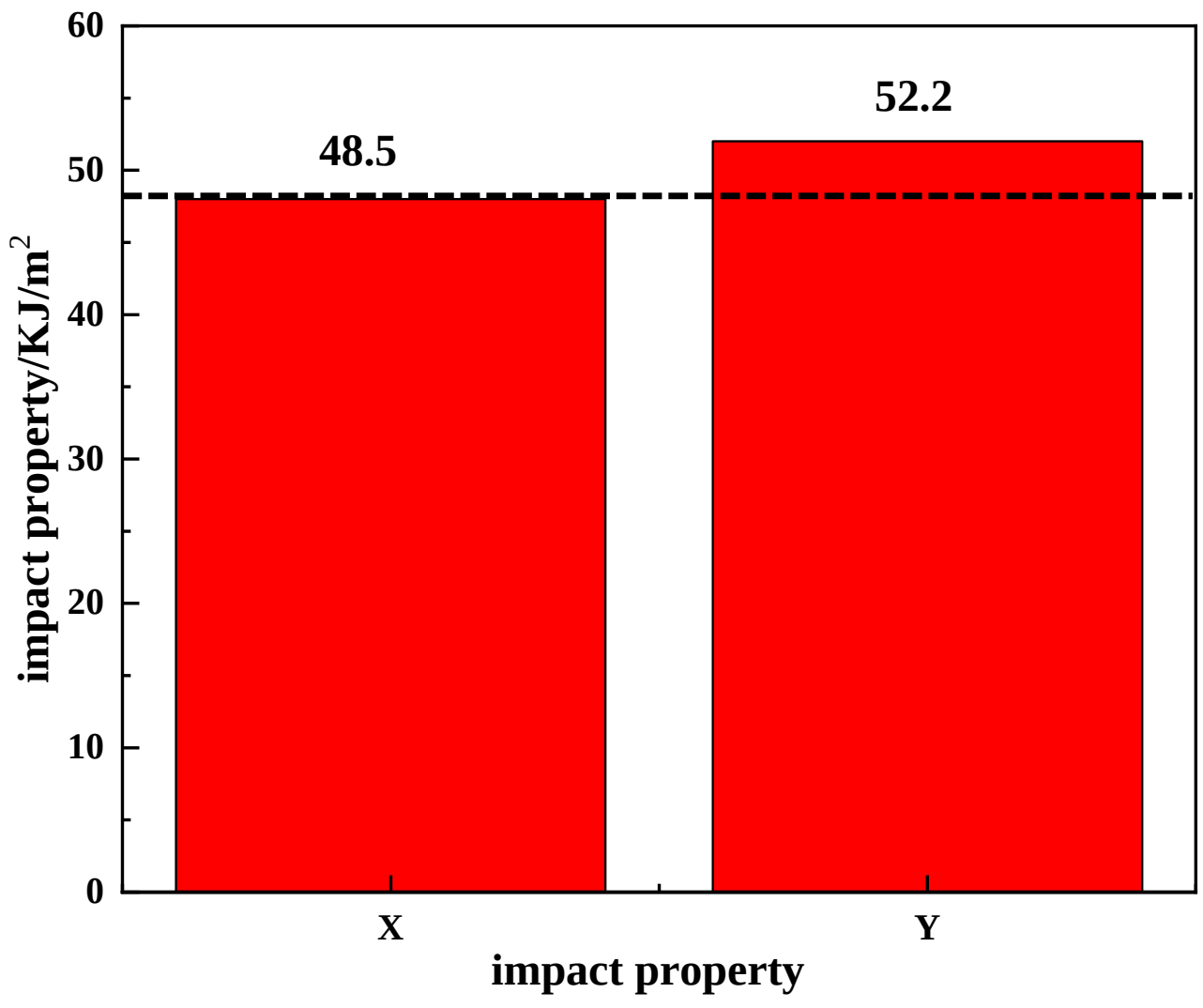

Fig. 7 Impact properties of additive samples

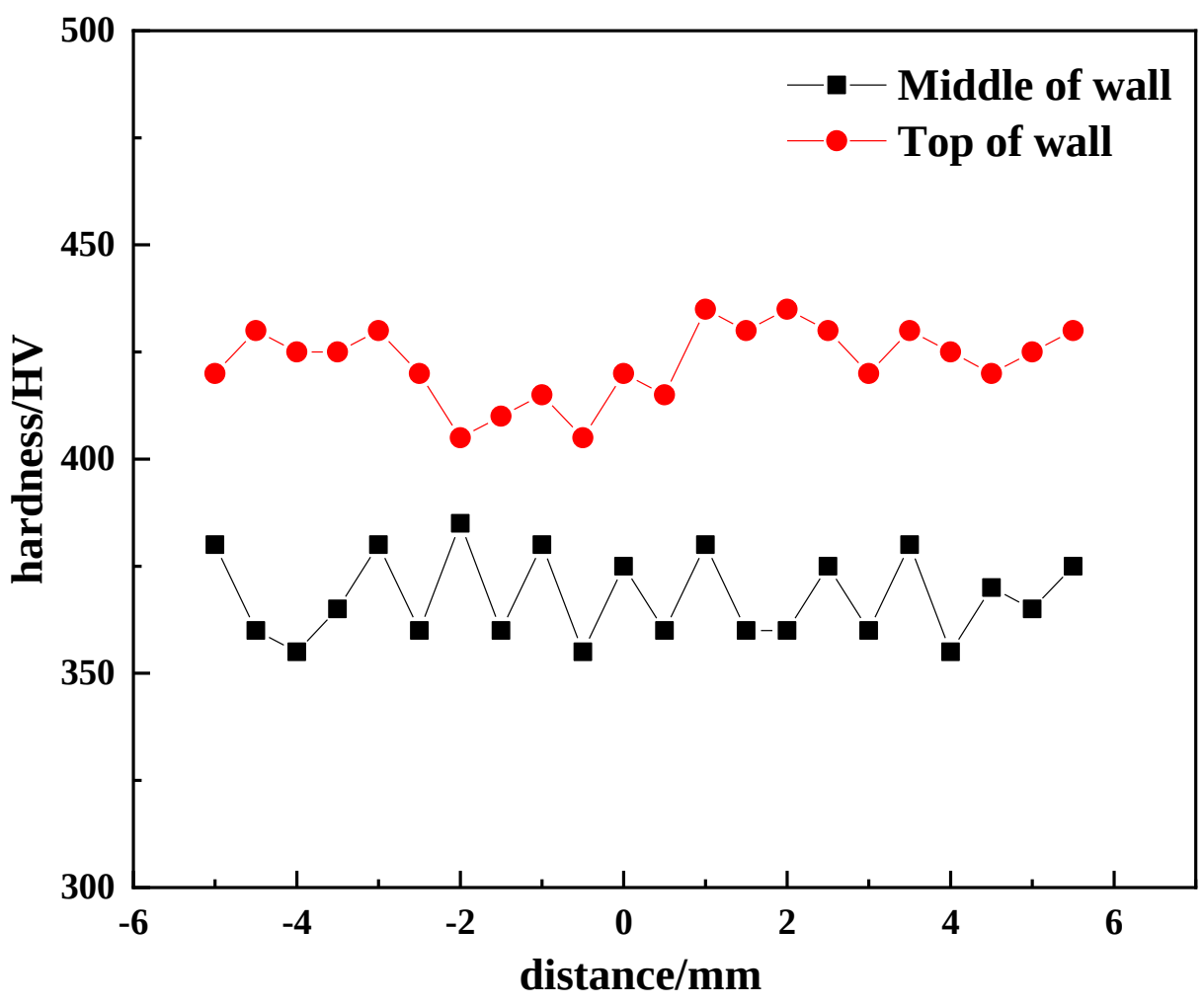

Fig. 8 The hardness distribution diagram of the center distance of the wall accumulation layer

Click here to access/download
Supporting Information
Table files.docx
