# Supplementary material for: Study on microstructure and properties of titanium alloy wall fabricated by laser-TIG arc
Source: PLoS One. 2026 Sep 15;21(9):e0355487. doi: 10.1371/journal.pone.0355487 (PMC13577510; doi:10.1371/journal.pone.0355487)
Supplement: Supplementary file 2 [file pone.0355487.s003.docx]

Dear Editor and Reviewers:

Thanks for your comments on my manuscript “Study on microstructure and properties of titanium alloy wall fabricated by laser-TIG arc” (ID: PONE-D-26-14242). Those comments are all valuable and very helpful for revising and improving our paper, as well as the important guiding significance to our research. We have studied comments carefully and have made the correction which we hope meet with approval. All of the revisions have been made in red in the revised manuscript and point-by-point responses are follows:

**Q1. Improve the abstract using the adopted methodology.**

A1: Thank you very much for your comments and your comment is very suggestive.

Using TC4 titanium alloy welding wire as raw material, through laser-TIG arc hybrid heat source wire additive manufacturing technology, combined with reciprocating scanning method for deposition, the effects of different process parameters on the tensile properties, impact properties and hardness of additive specimens were studied. The results show that: (1) With the increase of laser power, the average layer width of the formed part increases from 7.1 mm to 12.1 mm, an increase of 59 %; the average layer height decreased from 1.95 mm to 1.56 mm, a decrease of 20 %. (2) When the laser power is 1100 W, the transverse tensile strength is 896 MPa, the longitudinal tensile strength is 883 MPa, and the difference between transverse and longitudinal tensile strength is 13 MPa. The elongation after transverse fracture is 9.31 %, the elongation after longitudinal fracture is 10.23 %, and the difference of elongation after transverse and longitudinal fracture is 0.92 %. (3) The impact energy of the additive samples at room temperature (25°C) in two directions is 48.5 J·cm^-^² (X direction) and 52.2K J·cm^-^² (Y direction), respectively, and the microhardness of the top of the wall is higher than that of the middle end of the wall. This study provides an important experimental basis for the engineering application of laser-TIG arc additive manufacturing technology of TC4 titanium alloy.

**Q2. The correct form is MPa. Check the spacing between all text values and the unit of measurement.**

A2: Thank you very much for your comments and your comment is very suggestive. We have made corrections in accordance with your valuable comments, and all changes are shown in red in the revised manuscript.

**Q3. In introduction, is low thermal conductivity an excellent characteristic?**

A3: Thank you very much for your comments and your comment is very suggestive.

Titanium and its alloys have a series of excellent properties, such as low density, high specific strength and specific stiffness, corrosion resistance and fatigue resistance, high temperature performance and good welding performance, and are praised as the third metal. With the rapid development of aviation and other industries, the requirements for titanium alloy performance indicators are becoming more and more stringent. In order to meet the use standards of key structural parts such as advanced aero-engines, the development of efficient preparation processes for titanium alloys has become one of the core research hotspots in the field of materials for a long time now and in the future^[1-8]^.

**Q4. In introduction, the second paragraph is too long. It would be helpful to divide it into more paragraphs.**

A4: Thank you very much for your comments and your comment is very suggestive.

Many scholars at home and abroad have done a lot of research on additive manufacturing technology. The study of Akerfeldt *et al*.^[9]^ shows that the coarse columnarβgrains grown along the additive direction are one of the main factors affecting the anisotropy of the additive components. Edwards *et al*.^[10]^ analyzed the effect of pores on the overall mechanical properties of the samples fabricated by laser deposition. Razavi *et al*.^[11]^ used TSLM technology to prepare Ti-6Al-4V titanium alloy, and found that the surface quality of the workpiece produced by this method is high, and the defects are all on the surface of the workpiece. Brand *et al*.^[12]^ carried out heat treatment on the subsequent laser additive manufacturing TC4 titanium alloy samples, and found that the mechanical properties of the samples were improved and the strength was improved. Baufeld *et al*.^[13]^ found that the upper limit of fatigue strength of titanium alloys fabricated by additive manufacturing was increased by heat treatment. On this basis, Vrancken *et al*.^[14]^ increased the temperature of heat treatment. When the transition temperature of a certain phase is reached, the mechanical properties of titanium alloys can be significantly improved. Zhang *et al*.^[15]^ take a special temperature of titanium alloy annealing treatment, the sample, the material properties of uniform distribution, anisotropy has been significantly improved.

Pardal *et al*.^[16]^ verified that the laser can stabilize the arc in the heat conduction mode, and the effective width utilization rate of the titanium alloy component is increased by 70.8 %, which significantly improves the forming quality of the component. Rezaei A *et al*.^[17-18]^ found that the grain size of LPBF aluminum alloy in keyhole mode was smaller and the proportion of low-angle grain boundaries was higher, which promoted the PLC effect and achieved three times of plasticity improvement. Cai *et al*.^[19]^ prepared AZ31 magnesium alloy by laser-arc coaxial composite additive manufacturing technology. The average tensile strength in the horizontal and vertical directions is 224 MPa and 222 MPa, respectively, which is isotropic. Compared with the traditional single heat source additive manufacturing technology, the laser-TIG arc hybrid additive manufacturing technology can significantly improve the manufacturing efficiency, improve the microstructure morphology, refine the grains, and improve the comprehensive performance of the sample. Obviously, the laser-arc hybrid process has a significant positive impact on the forming quality, microstructure and properties of titanium alloy, stainless steel, aluminum alloy and other materials. However, there are few reports on the research of laser-arc hybrid additive manufacturing of titanium alloy.

**Q5. The introduction failed to better explain the objective of the work.**

A5: Thank you very much for your comments and your comment is very suggestive.

Titanium and its alloys have a series of excellent properties, such as low density, high specific strength and specific stiffness, corrosion resistance and fatigue resistance, high temperature performance and good welding performance, and are praised as the third metal. With the rapid development of aviation and other industries, the requirements for titanium alloy performance indicators are becoming more and more stringent. In order to meet the use standards of key structural parts such as advanced aero-engines, the development of efficient preparation processes for titanium alloys has become one of the core research hotspots in the field of materials for a long time now and in the future^[1-8]^.

Many scholars at home and abroad have done a lot of research on additive manufacturing technology. The study of Akerfeldt *et al*.^[9]^ shows that the coarse columnarβgrains grown along the additive direction are one of the main factors affecting the anisotropy of the additive components. Edwards *et al*.^[10]^ analyzed the effect of pores on the overall mechanical properties of the samples fabricated by laser deposition. Razavi *et al*.^[11]^ used TSLM technology to prepare Ti-6Al-4V titanium alloy, and found that the surface quality of the workpiece produced by this method is high, and the defects are all on the surface of the workpiece. Brand *et al*.^[12]^ carried out heat treatment on the subsequent laser additive manufacturing TC4 titanium alloy samples, and found that the mechanical properties of the samples were improved and the strength was improved. Baufeld *et al*.^[13]^ found that the upper limit of fatigue strength of titanium alloys fabricated by additive manufacturing was increased by heat treatment. On this basis, Vrancken *et al*.^[14]^ increased the temperature of heat treatment. When the transition temperature of a certain phase is reached, the mechanical properties of titanium alloys can be significantly improved. Zhang *et al*.^[15]^ take a special temperature of titanium alloy annealing treatment, the sample, the material properties of uniform distribution, anisotropy has been significantly improved.

Pardal *et al*.^[16]^ verified that the laser can stabilize the arc in the heat conduction mode, and the effective width utilization rate of the titanium alloy component is increased by 70.8 %, which significantly improves the forming quality of the component. Rezaei A *et al*.^[17-18]^ found that the grain size of LPBF aluminum alloy in keyhole mode was smaller and the proportion of low-angle grain boundaries was higher, which promoted the PLC effect and achieved three times of plasticity improvement. Cai *et al*.^[19]^ prepared AZ31 magnesium alloy by laser-arc coaxial composite additive manufacturing technology. The average tensile strength in the horizontal and vertical directions is 224 MPa and 222 MPa, respectively, which is isotropic. Compared with the traditional single heat source additive manufacturing technology, the laser-TIG arc hybrid additive manufacturing technology can significantly improve the manufacturing efficiency, improve the microstructure morphology, refine the grains, and improve the comprehensive performance of the sample. Obviously, the laser-arc hybrid process has a significant positive impact on the forming quality, microstructure and properties of titanium alloy, stainless steel, aluminum alloy and other materials. However, there are few reports on the research of laser-arc hybrid additive manufacturing of titanium alloy.

In this thesis, TC4 titanium alloy, which is widely used in the field of aerospace engineering, is used as the main test material. Through the laser-TIG arc hybrid heat source wire additive manufacturing technology, combined with the reciprocating scanning method for single-layer deposition, the mechanism and influence of different process parameters on the tensile properties, impact properties and hardness of the additive test piece are studied. In the cause of solve the problems of difficult forming of titanium alloy and difficult processing of precision structural parts, the regulation of process parameters on the forming morphology and deposition efficiency of thin-walled dimensions is realized.

**Q6. In Material and Methods, the figures and tables cited in the paragraph lack detail.**

A6: Thank you very much for your comments and your comment is very suggestive.

The TC4 wire with a diameter of 1.2 mm was used as the raw material, and the TC4 titanium alloy substrate was used as the substrate. The specific size was 150 mm×200 mm×20 mm. The chemical composition of the material is shown in **Table 1**. In this experiment, a laser-TIG arc hybrid additive manufacturing platform was built, and the schematic diagram is shown in **Figure 1**. The additive manufacturing platform mainly includes fiber laser, KUKA robot arm, wire feeder, TIG welding gun and vacuum protection box. The fiber laser uses a kilowatt YLS-10000-SS4 fiber laser produced by IPG company in the United States, with a focal length of 470 mm and a wavelength of 1070 ± 5 nm. The KUKA six-axis linkage robot arm is used to control the additive manufacturing process, which can accurately realize the planning of complex process paths. In the additive test, the deposition was carried out by reciprocating scanning, and 1 ~ 8 layers of walls were manufactured respectively. After the deposition of each layer, the next layer was deposited by air cooling for 40 s.

Before the test, the titanium wire wheel was used to clean the oxide film on the surface of the substrate, and acetone was used to remove oil and dust. The optimum laser power of laser-TIG arc hybrid wire additive manufacturing TC4 titanium alloy wall is 1100P/W, and other process window parameters are shown in **Table 2**. The core of substrate preheating in laser-arc additive manufacturing of titanium alloy is to reduce temperature gradient, suppress cracks, reduce residual stress, and improve microstructure and properties, which is the key process to ensure the forming quality. Therefore, the substrate is preheated at 150 °C for 8 min.

The hardness test uses the German KB automatic Vickers hardness tester to test the microhardness of the section of the sample after grinding and polishing. According to GB/T4340.1-2009, the loading force used in the test is 200 g. The standard tensile specimens (parallel section 5 mm) were prepared according to GB/T 228.1-2021 in both vertical and horizontal directions, and the tensile test was carried out by INSTRON5985-250 kN material testing machine. V-shaped impact specimens (10 mm×10 mm×55 mm) were prepared according to GB/T 229-2020, and the impact test was carried out at room temperature (25 °C) by ZBC2602-B pendulum impact tester. For the microhardness test, three test points were taken at the same height of the sample to measure the hardness, and the average of the three test points was finally taken as the final test result. For tensile test and impact test, three samples are also tested respectively, and the average value of the three samples is the final test result.

**Table 1** Chemical composition of TC4 titanium alloy wire and substrate(mass fraction)

| **Ingredient** | **Al** | **V** | **N** | **O** | **Fe** | **C** | **Ti** |
| --- | --- | --- | --- | --- | --- | --- | --- |
| **Wire** | 6.02 | 3.91 | 0.01 | 0.09 | 0.17 | 0.08 | Bal. |
| **Substrate** | 6.03 | 4.02 | 0.02 | 0.12 | 0.15 | 0.02 | Bal. |

**Fig. 1** Schematic diagram of laser⁃arc hybrid additive manufacturing platform

**Table 2** Laser⁃arc hybrid additive manufacturing TC4 titanium alloy optimal process

| parameter | laser power  *P/*W | welding current  *I/*A | arc voltage  *U/V* | wire feed rate  *v_f_/*(m.min^-1^) | deposit velocity  *v_w_/*(m.min^-1^) | laser-arc distance  *D/*mm |
| --- | --- | --- | --- | --- | --- | --- |
| numerical value | 1100 | 156 | 20 | 6 | 0.3 | 1.5 |

**Q7. In Results and Discussions, the mechanism by which the additive manufacturing process influences the microstructure and mechanical properties of the alloy studied was not explained in detail. The literature should be used to clarify the results.**

A7: Thank you very much for your comments and your comment is very suggestive.

**3.3 Impact performance analysis of laser-TIG arc hybrid wire additive manufacturing wall**

When the laser power is 1100W, the impact energy of the additive sample is measured at room temperature (25^o^C) in both X and Y directions, and the results are shown in **Fig.6**. It can be seen from **Fig.6** that the impact energy of the additive sample at room temperature (25^o^C) in two directions is 48.5 J·cm^-^² (X direction) and 52.2 J·cm^-^² (Y direction), respectively, which meet the requirements of CCS material welding and specification 2023. The impact toughness of the additive specimen is low. The main reason is that TC4 titanium alloy is a dual-phase titanium alloy. In the process of additive solidification, the grain boundary *β* phase precipitated at the boundary of the acicular *α* phase becomes a weak phase during the impact process, which makes the intergranular fracture occur during the impact process, and the energy needed to be absorbed during the test is small. The results are consistent with those of C. Fu ^[20]^.

**Fig.6** Impact properties of additive samples

**3.4 Microhardness analysis of laser-TIG arc hybrid wire additive manufacturing wall**

When the laser power is 1100W, the microhardness of the deposit layer at the top and middle of the wall is detected respectively. The hardness distribution of the deposit layer of the single-channel multi-layer forming wall is shown in **Fig.7**. From the diagram, it can be seen that the microhardness at the top of the wall is higher than that at the middle end of the wall. Due to the fast heat dissipation and cooling rate at the top of the wall, the primary *α* phase and martensite *α* phase are refined and the orientation is dispersed, and the hardness is higher than that of the middle accumulation layer. The hardness fluctuation is due to the difference in microstructure caused by the different heat input between the accumulation layer and the remelting zone. The accumulation layer is mainly composed of *α* and *α* + *β*, while the interlayer remelting zone is mainly composed of lamellar *α* phase, and the lamellar is thicker and the size is larger. Due to the fast heat dissipation and cooling rate at the top of the wall, the primary *α* phase and martensite α phase are refined and the orientation is dispersed, and the hardness is higher than that of the middle accumulation layer. The results are consistent with those of C. Wang ^[21]^.

**Fig.7** The hardness distribution diagram of the center distance of the wall accumulation layer

**Q8. Where are the references from the last five years? It needs updating.**

A8: Thank you very much for your comments and your comment is very suggestive.

**References**

[1] S.Vijayakumar, Optimization of friction stir welding parameters for dissimilar aluminium alloys using RSM-GRA and RSM-TOPSIS: Towards sustainable manufacturing in industry 4.0, Rineng, **000**, 27(2025). https://doi.org/10.1016/j.rineng.2025.107054
[2] D. Bandhu, Multiobjective optimization of RMD welding parameters for ASTM A387 steel using Taguchi method and artificial neural networks, Meas, **120114**, 262(2026). <https://doi.org/10.1016/j.measurement.2025.120114>
[3] D. Bandhu, F. Djavanroodi, G. Shaikshavali, Effect of metal-cored filler wire on surface morphology and micro-hardness of regulated metal deposition welded ASTM A387-Gr.11-Cl.2 steel plates, Materials. **19**, 15(2022). https://doi.org/10.3390/ma15196661
[4] A.Yadav, M. Srivastava, P. Jain. Investigation of bead morphology and mechanical behaviour for metal inert gas welding-based WAAM in pulsed mode metal transfer on 316LSi stainless steel, J. Adhes. Sci. Technol. **5**, 38(2024). https://doi.org/10.1080/01694243.2023.2241642
[5] D. Bandhu, K. Abhishek, Assessment of weld bead geometry in modified shortcircuiting gas metal arc welding process for low alloy steel. Mater. Manuf. Processes. **12**, 36(2021). https://doi.org/10.1080/10426914.2021.1906897
[6] S. Semiatin, An overview of the thermomechanical processing of α/β titanium alloys: current status and future research opportunities, Metall. Mater. Trans. A. **51**, 2593 (2020). https://link.springer.com/article/10.1007/s11661-020-05625-3
[7] M. Motyka, Martensite formation and decomposition during traditional and AM processing of two phase titanium alloys-an overview, Metals. **3**, 11 (2021). https://www.mdpi.com/2075-4701/11/3/481
[8] Y. Hao, Y. Huang, K. Zhao, Research on the microstructure and mechanical properties of doubled annealed laser melting deposition TC11 titanium alloy, Opt. Laser. Technol. **5**, 150 (2022). <https://www.sciencedirect.com/science/article/abs/pii/S0030399222001402>
[9] P. Akerfeldt, M. L. Antti, R. Pederson, Influence of microstructure on mechanical properties of laser metal wire-deposited Ti-6Al-4V, Mat. Sci. Eng. A-Struct. **674**, 428 (2016). https://www.sciencedirect.com/science/article/abs/pii/S0921509316307924
[10] P. Edwards, M. Ramulu, Fatigue performance evaluation of selective laser melted Ti-6Al-4V, Mat Sci Eng. **598**, 327 (2014). https://www.sciencedirect.com/science/article/abs/pii/S0921509314000720
[11] S. M. Razavi, P. Ferro, F. Berto, Fatigue strength of blunt v-notched sppeciens produced by selective laser melting of Ti-6Al-4V, Theor. Appl. Fract. Mec. **97**, 376 (2017). https://www.sciencedirect.com/science/article/abs/pii/S0167844217302884
[12] E. Brandl, F. Palm, V. Michailov, Mechanical properties of additive manufactured titanium (Ti-6Al-4V) blocks deposited by a solid-state laser and wire, Mater. Design. **32**, 10 (2011). https://www.sciencedirect.com/science/article/abs/pii/S0261306911004705
[13] B. Baufeld, E. Brandl. Wire based additive layer manufacturing: Comparison of microstructure and mechanical properties of Ti-6Al-4V components fabricated by laser-beam deposition and shaped metal deposition, J. Mater. Process. Tech. **211**, 6 (2011). https://www.sciencedirect.com/science/article/abs/pii/S0924013611000306
[14] B. Vrancken, L. Thijs, J. P. Kruth, Heat treatment of Ti6Al4V produced by Selective Laser Melting: Microstructure and mechanical properties, J. Alloy. Compd. **541**, 177 (2012). https://www.sciencedirect.com/science/article/abs/pii/S0925838812011826
[15] Q. Zhang, J. Chen, Zhao Z, Microstructure and anisotropic tensile behavior of laser additive manufactured TC21 titanium alloy, Mat. Sci. Eng. A-Struct. **673**, 204 (2016). <https://www.sciencedirect.com/science/article/abs/pii/S092150931630795X>
[16] G. PARDAL, F. MARTINA, S. WILLIAMS, Laser stabilization of GMAW additive manufacturing of Ti-6Al4V components, J. Mater. Process. Technol., **8**, 272 (2019). https://www.[10.1016/j.jmatprotec.2019.04.036](https://doi.org/10.1016/j.jmatprotec.2019.04.036)
[17] A. REZAEI, K. M. Keshavarz, S. Patel, Microstructural tailoring to promote Portevin-Le Chatelier effect in laser powder bed fusion fabricated AlMgScZr alloy, Mat Sci Eng A-Struct, **3**, 95 (2026). https://www.10.1016/j.msea.2025.149708
[18] A. REZAEI, K. M. Keshavarz, Melting mode-driven processing diagram for nanoparticle enhanced high-strength aluminum alloy processed by laser powder bed fusion, Mater. Des., **2**, 25 (2025). https://www. DOI:[10.1016/j.matdes.2025.113794](https://doi.org/10.1016/j.matdes.2025.113794)
[19] X. Cai, Y. XU, X.FENG, Laser-arc coaxial hybrid additive manufacturing of fine-grained homogeneous AZ31 magnesium alloy, J. Manuf. Process., **157**, 2 (2026). https://www. DOI:[10.1016/j.jmapro.2025.12.013](https://doi.org/10.1016/j.jmapro.2025.12.013)
[20] C. Fu, Y. Yin, Study on microstructure and properties of TC4 titanium alloy by laser coaxial wire feeding additive manufacturing, Electric welding machine, **55**, 2 (2025). https://www. 10.7512/j.issn.1001-2303.2025.02.08
[21] C. Wang, Finite element simulation and experimental analysis of metal TC4 additive manufacturing defects，xi'an university of technology, 2024.

With best regards.
