# Supplementary material for: Study on microstructure and properties of titanium alloy wall fabricated by laser-TIG arc
Source: PLoS One. 2026 Sep 15;21(9):e0355487. doi: 10.1371/journal.pone.0355487 (PMC13577510; doi:10.1371/journal.pone.0355487)
Supplement: Supplementary file 3 [file pone.0355487.s004.docx]

Dear Editor and Reviewers:

Thanks for your comments on my manuscript “Study on microstructure and properties of titanium alloy wall fabricated by laser-TIG arc” (ID: PONE-D-26-14242). Those comments are all valuable and very helpful for revising and improving our paper, as well as the important guiding significance to our research. We have studied comments carefully and have made the correction which we hope meet with approval. All of the revisions have been made in red in the revised manuscript and point-by-point responses are follows:

**1. All text in red highlighted should be revised in order to solve Grammar and Spelling errors;**

A1: Thank you very much for your comments and your comment is very suggestive. All revisions are done in red in the revised manuscript.

**2. Into the section 2 and into Results and Discussion, all values and dimensions should obligatory be accompanied with their corresponding error ranges.**

A2: Thank you very much for your comments and your comment is very suggestive.

The hardness test uses the German KB automatic Vickers hardness tester to test the microhardness of the section of the sample after grinding and polishing. According to GB/T4340.1-2009, the loading force used in the test is 200 g. The standard tensile specimens (parallel section 5 mm) were prepared according to GB/T 228.1-2021 in both vertical and horizontal directions, and the tensile test was carried out by INSTRON5985-250 kN material testing machine. V-shaped impact specimens (10 mm×10 mm×55 mm) were prepared according to GB/T 229-2020, and the impact test was carried out at room temperature (25 °C) by ZBC2602-B pendulum impact tester. For the microhardness test, three test points were taken at the same height of the sample to measure the hardness, and the average of the three test points was finally taken as the final test result. For tensile test and impact test, three samples are also tested respectively, and the average value of the three samples is the final test result.

**3.2 Analysis of tensile properties of laser-TIG arc hybrid wire additive manufacturing wall**

When the laser power is 1100W, the tensile properties of TC4 titanium alloy in both transverse (X) and longitudinal (Y) directions are discussed. The results are shown in **Fig.3**. From Figure 3, it can be seen that when the laser power is 1100W, the transverse tensile strength is 896 MPa, the longitudinal tensile strength is 883 MPa, and the difference between the transverse and longitudinal tensile strength is 13 MPa; the transverse elongation after fracture is 9.31 %, the longitudinal elongation after fracture is 10.23 %, and the difference between transverse and longitudinal elongation after fracture is 0.92 %, indicating that the anisotropy of tensile properties is small. This low anisotropy is mainly due to the reciprocating deposition method: the interlayer deposition directions are perpendicular to each other, so that the grain structures in different directions are similar, and the grain orientations are basically the same, thus reducing the direction dependence of mechanical properties. It can be seen from Fig.3 that the error range of the three parallel experiments is small, the experimental accuracy is high, and the experimental accuracy is high.

**Fig.3** **Analysis of tensile propertie:** (a) tensile strength; (b) post-fracture elongation

**3.3 Impact performance analysis of laser-TIG arc hybrid wire additive manufacturing wall**

When the laser power is 1100W, the impact energy of the additive sample is measured at room temperature (25^o^C) in both X and Y directions, and the results are shown in **Fig.5**. It can be seen from Fig.5 that the impact energy of the additive sample at room temperature (25^o^C) in two directions is 48.5 J·cm^-^² (X direction) and 52.2 J·cm^-^² (Y direction), respectively, which meet the requirements of CCS material welding and specification 2023. According to Fig.5, the relative error of the three parallel experiments is small, and the experimental results have high experimental accuracy.The impact toughness of the additive specimen is low. The main reason is that TC4 titanium alloy is a dual-phase titanium alloy. In the process of additive solidification, the grain boundary *β* phase precipitated at the boundary of the acicular *α* phase becomes a weak phase during the impact process, which makes the intergranular fracture occur during the impact process, and the energy needed to be absorbed during the test is small. The results are consistent with those of C. Fu ^[20]^.

**Fig.5** Impact properties of additive samples

**3.4 Microhardness analysis of laser-TIG arc hybrid wire additive manufacturing wall**

When the laser power is 1100W, the microhardness of the deposit layer at the top and middle of the wall is detected respectively. The hardness distribution of the deposit layer of the single-channel multi-layer forming wall is shown in **Fig.6**. From the diagram, it can be seen that the microhardness at the top of the wall is higher than that at the middle end of the wall. Due to the fast heat dissipation and cooling rate at the top of the wall, the primary *α* phase and martensite *α* phase are refined and the orientation is dispersed, and the hardness is higher than that of the middle accumulation layer. The hardness fluctuation is due to the difference in microstructure caused by the different heat input between the accumulation layer and the remelting zone. The accumulation layer is mainly composed of *α* and *α*+*β*, while the interlayer remelting zone is mainly composed of lamellar *α* phase, and the lamellar is thicker and the size is larger. Due to the fast heat dissipation and cooling rate at the top of the wall, the primary *α* phase and martensite α phase are refined and the orientation is dispersed, and the hardness is higher than that of the middle accumulation layer. The results are consistent with those of C. Wang ^[21]^. According to Fig.6, the relative error of the three parallel experimental tests is small, and the experimental results have a small error with the average value, so the experimental results have high experimental accuracy.

**Fig.6** The hardness distribution diagram of the center distance of the wall accumulation layer

**3. New sentences into section 2 and, in same cases into Results and discussion, should be included in order to elucidate the repeatibility and reproducibility of the proposed investigation.**

A3: Thank you very much for your comments and your comment is very suggestive.

The hardness test uses the German KB automatic Vickers hardness tester to test the microhardness of the section of the sample after grinding and polishing. According to GB/T4340.1-2009, the loading force used in the test is 200 g. The standard tensile specimens (parallel section 5 mm) were prepared according to GB/T 228.1-2021 in both vertical and horizontal directions, and the tensile test was carried out by INSTRON5985-250 kN material testing machine. V-shaped impact specimens (10 mm×10 mm×55 mm) were prepared according to GB/T 229-2020, and the impact test was carried out at room temperature (25 °C) by ZBC2602-B pendulum impact tester. For the microhardness test, three test points were taken at the same height of the sample to measure the hardness, and the average of the three test points was finally taken as the final test result. For tensile test and impact test, three samples are also tested respectively, and the average value of the three samples is the final test result.

**3.2 Analysis of tensile properties of laser-TIG arc hybrid wire additive manufacturing wall**

When the laser power is 1100W, the tensile properties of TC4 titanium alloy in both transverse (X) and longitudinal (Y) directions are discussed. The results are shown in **Fig.3**. From Figure 3, it can be seen that when the laser power is 1100W, the transverse tensile strength is 896 MPa, the longitudinal tensile strength is 883 MPa, and the difference between the transverse and longitudinal tensile strength is 13 MPa; the transverse elongation after fracture is 9.31 %, the longitudinal elongation after fracture is 10.23 %, and the difference between transverse and longitudinal elongation after fracture is 0.92 %, indicating that the anisotropy of tensile properties is small. This low anisotropy is mainly due to the reciprocating deposition method: the interlayer deposition directions are perpendicular to each other, so that the grain structures in different directions are similar, and the grain orientations are basically the same, thus reducing the direction dependence of mechanical properties. It can be seen from Fig.3 that the error range of the three parallel experiments is small, the experimental accuracy is high, and the experimental accuracy is high.

**Fig.3** **Analysis of tensile propertie:** (a) tensile strength; (b) post-fracture elongation

**3.3 Impact performance analysis of laser-TIG arc hybrid wire additive manufacturing wall**

When the laser power is 1100W, the impact energy of the additive sample is measured at room temperature (25^o^C) in both X and Y directions, and the results are shown in **Fig.5**. It can be seen from Fig.5 that the impact energy of the additive sample at room temperature (25^o^C) in two directions is 48.5 J·cm^-^² (X direction) and 52.2 J·cm^-^² (Y direction), respectively, which meet the requirements of CCS material welding and specification 2023. According to Fig.5, the relative error of the three parallel experiments is small, and the experimental results have high experimental accuracy.The impact toughness of the additive specimen is low. The main reason is that TC4 titanium alloy is a dual-phase titanium alloy. In the process of additive solidification, the grain boundary *β* phase precipitated at the boundary of the acicular *α* phase becomes a weak phase during the impact process, which makes the intergranular fracture occur during the impact process, and the energy needed to be absorbed during the test is small. The results are consistent with those of C. Fu ^[20]^.

**Fig.5** Impact properties of additive samples

**3.4 Microhardness analysis of laser-TIG arc hybrid wire additive manufacturing wall**

When the laser power is 1100W, the microhardness of the deposit layer at the top and middle of the wall is detected respectively. The hardness distribution of the deposit layer of the single-channel multi-layer forming wall is shown in **Fig.6**. From the diagram, it can be seen that the microhardness at the top of the wall is higher than that at the middle end of the wall. Due to the fast heat dissipation and cooling rate at the top of the wall, the primary *α* phase and martensite *α* phase are refined and the orientation is dispersed, and the hardness is higher than that of the middle accumulation layer. The hardness fluctuation is due to the difference in microstructure caused by the different heat input between the accumulation layer and the remelting zone. The accumulation layer is mainly composed of *α* and *α*+*β*, while the interlayer remelting zone is mainly composed of lamellar *α* phase, and the lamellar is thicker and the size is larger. Due to the fast heat dissipation and cooling rate at the top of the wall, the primary *α* phase and martensite α phase are refined and the orientation is dispersed, and the hardness is higher than that of the middle accumulation layer. The results are consistent with those of C. Wang ^[21]^. According to Fig.6, the relative error of the three parallel experimental tests is small, and the experimental results have a small error with the average value, so the experimental results have high experimental accuracy.

**Fig.6** The hardness distribution diagram of the center distance of the wall accumulation layer

**4. Table 5 should de reworked and the corresponding error ranges adopted.**

A4: Thank you very much for your comments and your comment is very suggestive. All revisions are done in red in the revised manuscript.

**Table 5** The hardness distribution value of the deposited layer

| Distance/mm | Middle/HV | | | Top/HV | | |
| --- | --- | --- | --- | --- | --- | --- |
|  | the first sample | the second sample | the third sample | the first sample | the second sample | the third sample |
| -5.0 | 380 | 383 | 384 | 420 | 422 | 423 |
| -4.5 | 360 | 365 | 363 | 430 | 435 | 433 |
| -4.0 | 355 | 359 | 360 | 425 | 430 | 426 |
| -3.5 | 365 | 367 | 367 | 425 | 426 | 428 |
| -3.0 | 380 | 382 | 383 | 430 | 432 | 434 |
| -2.5 | 360 | 363 | 365 | 420 | 424 | 424 |
| -2.0 | 385 | 389 | 388 | 405 | 410 | 409 |
| -1.5 | 360 | 365 | 361 | 410 | 414 | 415 |
| -1.0 | 380 | 382 | 384 | 415 | 420 | 418 |
| -0.5 | 365 | 369 | 370 | 405 | 408 | 410 |
| 0.0 | 385 | 389 | 390 | 420 | 422 | 423 |
| 0.5 | 370 | 373 | 374 | 415 | 419 | 417 |
| 1.0 | 385 | 386 | 389 | 435 | 439 | 439 |
| 1.5 | 360 | 365 | 363 | 430 | 435 | 435 |
| 2.0 | 360 | 362 | 365 | 435 | 438 | 439 |
| 2.5 | 385 | 390 | 387 | 430 | 435 | 433 |
| 3.0 | 360 | 364 | 365 | 420 | 423 | 423 |
| 3.5 | 380 | 383 | 384 | 430 | 434 | 434 |
| 4.0 | 355 | 360 | 358 | 425 | 428 | 429 |
| 4.5 | 370 | 373 | 372 | 420 | 424 | 423 |
| 5.0 | 365 | 367 | 371 | 425 | 428 | 427 |
| 5.5 | 375 | 379 | 377 | 430 | 432 | 433 |

**5. Figs. 2 and 3 and Figs. 4 and 5 should be reworked into a unique panel, respectively. Besides, its caption should be revised and indications “a” and “b” should be depicted. These figures can be disposed side by side.**

A5: Thank you very much for your comments and your comment is very suggestive. All revisions are done in red in the revised manuscript.

**Fig. 2** The effect of laser power : (a) the average layer width ; (b) the average layer height

**Fig.3** **Analysis of tensile propertie:** (a) tensile strength; (b) post-fracture elongation

**6. Figs. 5, 6 and 7 should obligatory be revised and reworked. Error ranges should be adopted. When these error ranges are included, a new discussion seems to be resulted. Based on this, it is hardly suggested that the discussion be meticulously revised, as a function of the inclusion of the error ranges. It seems that similar behavior is occurred. Please, revised it.**

A6: Thank you very much for your comments and your comment is very suggestive. All revisions are done in red in the revised manuscript.

**3.2 Analysis of tensile properties of laser-TIG arc hybrid wire additive manufacturing wall**

When the laser power is 1100W, the tensile properties of TC4 titanium alloy in both transverse (X) and longitudinal (Y) directions are discussed. The results are shown in **Fig.3**. From Figure 3, it can be seen that when the laser power is 1100W, the transverse tensile strength is 896 MPa, the longitudinal tensile strength is 883 MPa, and the difference between the transverse and longitudinal tensile strength is 13 MPa; the transverse elongation after fracture is 9.31 %, the longitudinal elongation after fracture is 10.23 %, and the difference between transverse and longitudinal elongation after fracture is 0.92 %, indicating that the anisotropy of tensile properties is small. This low anisotropy is mainly due to the reciprocating deposition method: the interlayer deposition directions are perpendicular to each other, so that the grain structures in different directions are similar, and the grain orientations are basically the same, thus reducing the direction dependence of mechanical properties. It can be seen from Fig.3 that the error range of the three parallel experiments is small, the experimental accuracy is high, and the experimental accuracy is high.

**Fig.3** **Analysis of tensile propertie:** (a) tensile strength; (b) post-fracture elongation

**3.3 Impact performance analysis of laser-TIG arc hybrid wire additive manufacturing wall**

When the laser power is 1100W, the impact energy of the additive sample is measured at room temperature (25^o^C) in both X and Y directions, and the results are shown in **Fig.5**. It can be seen from Fig.5 that the impact energy of the additive sample at room temperature (25^o^C) in two directions is 48.5 J·cm^-^² (X direction) and 52.2 J·cm^-^² (Y direction), respectively, which meet the requirements of CCS material welding and specification 2023. According to Fig.5, the relative error of the three parallel experiments is small, and the experimental results have high experimental accuracy.The impact toughness of the additive specimen is low. The main reason is that TC4 titanium alloy is a dual-phase titanium alloy. In the process of additive solidification, the grain boundary *β* phase precipitated at the boundary of the acicular *α* phase becomes a weak phase during the impact process, which makes the intergranular fracture occur during the impact process, and the energy needed to be absorbed during the test is small. The results are consistent with those of C. Fu ^[20]^.

**Fig.5** Impact properties of additive samples

**3.4 Microhardness analysis of laser-TIG arc hybrid wire additive manufacturing wall**

When the laser power is 1100W, the microhardness of the deposit layer at the top and middle of the wall is detected respectively. The hardness distribution of the deposit layer of the single-channel multi-layer forming wall is shown in **Fig.6**. From the diagram, it can be seen that the microhardness at the top of the wall is higher than that at the middle end of the wall. Due to the fast heat dissipation and cooling rate at the top of the wall, the primary *α* phase and martensite *α* phase are refined and the orientation is dispersed, and the hardness is higher than that of the middle accumulation layer. The hardness fluctuation is due to the difference in microstructure caused by the different heat input between the accumulation layer and the remelting zone. The accumulation layer is mainly composed of *α* and *α*+*β*, while the interlayer remelting zone is mainly composed of lamellar *α* phase, and the lamellar is thicker and the size is larger. Due to the fast heat dissipation and cooling rate at the top of the wall, the primary *α* phase and martensite α phase are refined and the orientation is dispersed, and the hardness is higher than that of the middle accumulation layer. The results are consistent with those of C. Wang ^[21]^. According to Fig.6, the relative error of the three parallel experimental tests is small, and the experimental results have a small error with the average value, so the experimental results have high experimental accuracy.

**Fig.6** The hardness distribution diagram of the center distance of the wall accumulation layer

**7.In Fig. 7 error ranges and average line should be depicted in figure. Additionally, it is hardly suggested that at least one representative or typical image after indentation inside figure be also depicted.**

A7: Thank you very much for your comments and your comment is very suggestive. All revisions are done in red in the revised manuscript.

**Fig.6** The hardness distribution diagram of the center distance of the wall accumulation layer

**8. Fig. 5 has remained error in scale “Mpa”.**

A8: Thank you very much for your comments and your comment is very suggestive. All revisions are done in red in the revised manuscript.

**Fig.3** **Analysis of tensile propertie:** (a) tensile strength; (b) post-fracture elongation

**9. Since Fig. 4 shows the tensile behavior of the examined samples, it is mandatory that its resulting stress vs. strain curve de depicted. This implies that a new figure be adopted. For this purpose, stress vs strain curves with duplicate should be depicted. Complementary, the proposed bar graph type can be depicted inside figure.**

A9: Thank you very much for your comments and your comment is very suggestive.

**3.2 Analysis of tensile properties of laser-TIG arc hybrid wire additive manufacturing wall**

When the laser power is 1100W, the tensile properties of TC4 titanium alloy in both transverse (X) and longitudinal (Y) directions are discussed. The results are shown in **Fig.3**. From Figure 3, it can be seen that when the laser power is 1100W, the transverse tensile strength is 896 MPa, the longitudinal tensile strength is 883 MPa, and the difference between the transverse and longitudinal tensile strength is 13 MPa; the transverse elongation after fracture is 9.31 %, the longitudinal elongation after fracture is 10.23 %, and the difference between transverse and longitudinal elongation after fracture is 0.92 %, indicating that the anisotropy of tensile properties is small. This low anisotropy is mainly due to the reciprocating deposition method: the interlayer deposition directions are perpendicular to each other, so that the grain structures in different directions are similar, and the grain orientations are basically the same, thus reducing the direction dependence of mechanical properties. It can be seen from Fig.3 that the error range of the three parallel experiments is small, the experimental accuracy is high, and the experimental accuracy is high.**Figure 4** is the stress-strain curve of the sample in the X direction and the Y direction. It can be seen from the diagram that the numerical error of the yield strength measured by the three parallel tests is small, showing significant strain hardening characteristics.

**Fig.3** Analysis of tensile propertie: (a) tensile strength; (b) post-fracture elongation

**Fig.4** Stress-strain curves: (a) X-direction; (b) Y-direction

**10. When a stress vs. strain curve is depicted with its reproducibility (duplicate or triplicate), another important parameter is revealed, i.e. the yield strength. This was neglected in discussion. Please, revise this point and include it.**

A10: Thank you very much for your comments and your comment is very suggestive.

**3.2 Analysis of tensile properties of laser-TIG arc hybrid wire additive manufacturing wall**

When the laser power is 1100W, the tensile properties of TC4 titanium alloy in both transverse (X) and longitudinal (Y) directions are discussed. The results are shown in **Fig.3**. From Figure 3, it can be seen that when the laser power is 1100W, the transverse tensile strength is 896 MPa, the longitudinal tensile strength is 883 MPa, and the difference between the transverse and longitudinal tensile strength is 13 MPa; the transverse elongation after fracture is 9.31 %, the longitudinal elongation after fracture is 10.23 %, and the difference between transverse and longitudinal elongation after fracture is 0.92 %, indicating that the anisotropy of tensile properties is small. This low anisotropy is mainly due to the reciprocating deposition method: the interlayer deposition directions are perpendicular to each other, so that the grain structures in different directions are similar, and the grain orientations are basically the same, thus reducing the direction dependence of mechanical properties. It can be seen from Fig.3 that the error range of the three parallel experiments is small, the experimental accuracy is high, and the experimental accuracy is high.**Figure 4** is the stress-strain curve of the sample in the X direction and the Y direction. It can be seen from the diagram that the numerical error of the yield strength measured by the three parallel tests is small, showing significant strain hardening characteristics.

**Fig.3** Analysis of tensile propertie: (a) tensile strength; (b) post-fracture elongation

**Fig.4** Stress-strain curves: (a) X-direction; (b) Y-direction

**11. Also, into the list of references, it is clearly verified that no guidelines was adopted. Please, revise it. For instance , upper letter is mixed with lower letter.**

A11: Thank you very much for your comments and your comment is very suggestive. All revisions are done in red in the revised manuscript.

With best regards.
