# Supplementary material for: Study on microstructure and properties of titanium alloy wall fabricated by laser-TIG arc
Source: PLoS One. 2026 Sep 15;21(9):e0355487. doi: 10.1371/journal.pone.0355487 (PMC13577510; doi:10.1371/journal.pone.0355487)
Supplement: Peer review history

# Peer review: Decision Letter 0

**DOI:** 10.1371/journal.pone.0355487.r001

**Authors:** Wislei Riuper Osório

21 May 2026

Dear Dr. guo,

Thank you for submitting your manuscript to PLOS ONE. After careful consideration, we feel that it has merit but does not fully meet PLOS ONE’s publication criteria as it currently stands. Therefore, we invite you to submit a revised version of the manuscript that addresses the points raised during the review process.

==============================

**ACADEMIC EDITOR:**

==============================

Please submit your revised manuscript by Jul 05 2026 11:59PM. If you will need more time than this to complete your revisions, please reply to this message or contact the journal office at plosone@plos.org. When you're ready to submit your revision, log on to https://www.editorialmanager.com/pone/ and select the 'Submissions Needing Revision' folder to locate your manuscript file.

Please include the following items when submitting your revised manuscript:

- A letter that responds to each point raised by the academic editor and reviewer(s). You should upload this letter as a separate file labeled 'Response to Reviewers'.
- A marked-up copy of your manuscript that highlights changes made to the original version. You should upload this as a separate file labeled 'Revised Manuscript with Track Changes'.
- An unmarked version of your revised paper without tracked changes. You should upload this as a separate file labeled 'Manuscript'.

If you would like to make changes to your financial disclosure, please include your updated statement in your cover letter. Guidelines for resubmitting your figure files are available below the reviewer comments at the end of this letter.

If applicable, we recommend that you deposit your laboratory protocols in protocols.io to enhance the reproducibility of your results. Protocols.io assigns your protocol its own identifier (DOI) so that it can be cited independently in the future. For instructions see: https://journals.plos.org/plosone/s/submission-guidelines#loc-laboratory-protocols. Additionally, PLOS ONE offers an option for publishing peer-reviewed Lab Protocol articles, which describe protocols hosted on protocols.io. Read more information on sharing protocols at https://plos.org/protocols?utm_medium=editorial-email&utm_source=authorletters&utm_campaign=protocols.

As the corresponding author, your ORCID iD is verified in the submission system and will appear in the published article. PLOS supports the use of ORCID, and we encourage all coauthors to register for an ORCID iD and use it as well. Please encourage your coauthors to verify their ORCID iD within the submission system before final acceptance, as unverified ORCID iDs will not appear in the published article. *Only*  the individual author can complete the verification step; PLOS staff *cannot*  verify ORCID iDs on behalf of authors.

We look forward to receiving your revised manuscript.

Kind regards,

Wislei Riuper Osório

Academic Editor

PLOS One

Journal Requirements:

When submitting your revision, we need you to address these additional requirements.

1. Please ensure that your manuscript meets PLOS ONE's style requirements, including those for file naming. The PLOS ONE style templates can be found at

https://journals.plos.org/plosone/s/file?id=wjVg/PLOSOne_formatting_sample_main_body.pdf and https://journals.plos.org/plosone/s/file?id=ba62/PLOSOne_formatting_sample_title_authors_affiliations.pdf

2. PLOS requires an ORCID iD for the corresponding author in Editorial Manager on papers submitted after December 6th, 2016. Please ensure that you have an ORCID iD and that it is validated in Editorial Manager. To do this, go to ‘Update my Information’ (in the upper left-hand corner of the main menu), and click on the Fetch/Validate link next to the ORCID field. This will take you to the ORCID site and allow you to create a new iD or authenticate a pre-existing iD in Editorial Manager.

3. We note that you have indicated that there are restrictions to data sharing for this study. PLOS only allows data to be available upon request if there are legal or ethical restrictions on sharing data publicly. For more information on unacceptable data access restrictions, please see http://journals.plos.org/plosone/s/data-availability#loc-unacceptable-data-access-restrictions.

Before we proceed with your manuscript, please address the following prompts:

a) If there are ethical or legal restrictions on sharing a de-identified data set, please explain them in detail (e.g., data contain potentially identifying or sensitive patient information, data are owned by a third-party organization, etc.) and who has imposed them (e.g., a Research Ethics Committee or Institutional Review Board, etc.). Please also provide contact information for a data access committee, ethics committee, or other institutional body to which data requests may be sent.

b) If there are no restrictions, please upload the minimal anonymized data set necessary to replicate your study findings to a stable, public repository and provide us with the relevant URLs, DOIs, or accession numbers. For a list of recommended repositories, please see

https://journals.plos.org/plosone/s/recommended-repositories. You also have the option of uploading the data as Supporting Information files, but we would recommend depositing data directly to a data repository if possible.

We will update your Data Availability statement on your behalf to reflect the information you provide.

4. Please provide a complete Data Availability Statement in the submission form, ensuring you include all necessary access information or a reason for why you are unable to make your data freely accessible. If your research concerns only data provided within your submission, please write "All data are in the manuscript and/or supporting information files" as your Data Availability Statement.

5. Thank you for stating the following in your Competing Interests section:

no

Please complete your Competing Interests on the online submission form to state any Competing Interests. If you have no competing interests, please state "The authors have declared that no competing interests exist.", as detailed online in our guide for authors at http://journals.plos.org/plosone/s/submit-now

This information should be included in your cover letter; we will change the online submission form on your behalf.

6. Please update your submission to use the PLOS LaTeX template. The template and more information on our requirements for LaTeX submissions can be found at http://journals.plos.org/plosone/s/latex.

7. Please amend your authorship list in your manuscript file to include author yongchun guo.

8. Please amend the manuscript submission data (via Edit Submission) to include author YC. Guo, MY Li.

9. Your abstract cannot contain citations. Please only include citations in the body text of the manuscript, and ensure that they remain in ascending numerical order on first mention.

10. Please remove your figures from within your manuscript file, leaving only the individual TIFF/EPS image files, uploaded separately. These will be automatically included in the reviewers’ PDF**.**

11. We note that Figure(s) 1, 2 in your submission contain copyrighted images. All PLOS content is published under the Creative Commons Attribution License (CC BY 4.0), which means that the manuscript, images, and Supporting Information files will be freely available online, and any third party is permitted to access, download, copy, distribute, and use these materials in any way, even commercially, with proper attribution. For more information, see our copyright guidelines: http://journals.plos.org/plosone/s/licenses-and-copyright.

We require you to either (1) present written permission from the copyright holder to publish these figures specifically under the CC BY 4.0 license, or (2) remove the figures from your submission:

a. You may seek permission from the original copyright holder of Figure(s) 1, 2 to publish the content specifically under the CC BY 4.0 license.

We recommend that you contact the original copyright holder with the Content Permission Form (http://journals.plos.org/plosone/s/file?id=7c09/content-permission-form.pdf) and the following text:

“I request permission for the open-access journal PLOS ONE to publish XXX under the Creative Commons Attribution License (CCAL) CC BY 4.0 (http://creativecommons.org/licenses/by/4.0/). Please be aware that this license allows unrestricted use and distribution, even commercially, by third parties. Please reply and provide explicit written permission to publish XXX under a CC BY license and complete the attached form.”

Please upload the completed Content Permission Form or other proof of granted permissions as an "Other" file with your submission.

In the figure caption of the copyrighted figure, please include the following text: “Reprinted from [ref] under a CC BY license, with permission from [name of publisher], original copyright [original copyright year].”

b. If you are unable to obtain permission from the original copyright holder to publish these figures under the CC BY 4.0 license or if the copyright holder’s requirements are incompatible with the CC BY 4.0 license, please either i) remove the figure or ii) supply a replacement figure that complies with the CC BY 4.0 license. Please check copyright information on all replacement figures and update the figure caption with source information. If applicable, please specify in the figure caption text when a figure is similar but not identical to the original image and is therefore for illustrative purposes only.

12. Please include your tables as part of your main manuscript and remove the individual files. Please note that supplementary tables (should remain/ be uploaded) as separate "supporting information" files.

13. If the reviewer comments include a recommendation to cite specific previously published works, please review and evaluate these publications to determine whether they are relevant and should be cited. There is no requirement to cite these works unless the editor has indicated otherwise.

Additional Editor Comments:

Dear Authors;

Based on the Reviewers’ comments, a MAJOR revision is suggested. In this sense, it is hardly suggested that all comments and suggestions be solved and improved. Also, this Editor also suggest to a meticulous revision concerning to repeatability and reproducibility be provided. Please, revise all dimensions and physical units, and adopt the error ranges in all values and dimensions written in the proposed manuscript. Besides, a meticulous revision in Grammar and Spelling should also be adopted. After all comments and suggestions solved, please provide it final version to be resent to Reviewers. This, it is highly suggested that all “rebuttals” be meticulously provided, solved and discussed.

[Note: HTML markup is below. Please do not edit.]

Reviewers' comments:

Reviewer's Responses to Questions

**Comments to the Author**

1. Is the manuscript technically sound, and do the data support the conclusions?

Reviewer #1: Yes

Reviewer #2: Partly

**********

2. Has the statistical analysis been performed appropriately and rigorously?

Reviewer #1: Yes

Reviewer #2: No

**********

3. Have the authors made all data underlying the findings in their manuscript fully available?

The PLOS Data policy

Reviewer #1: Yes

Reviewer #2: Yes

**********

4. Is the manuscript presented in an intelligible fashion and written in standard English?

Reviewer #1: Yes

Reviewer #2: Yes

**********

Reviewer #1: After careful reading of the article, I strongly recommend it for major revision due to the following concerns:

1. Abstract is not well drafted. Write a structured abstract. The abstract must end with a conclusion and a recommendation from your work.

2. The introduction heavily cites papers from 2010 to 2022 but lacks references from 2024–2026, which weakens the study's motivation and currency.

3. The statement that “many scholars at home and abroad have been unable to meet the development needs” is overly vague and needs specific, quantifiable gaps to justify the hybrid approach.

4. The introduction fails to clearly define what unique advantage laser‑TIG hybrid offers over standalone laser or arc additive manufacturing for TC4 walls.

5. Actually, the introduction is too short. It does not highlight the findings from previous studies. It is suggested to incorporate more studies from the literature and to add a summary table for them.

6. Cite more contemporary studies to strengthen the research gap. In this regard, the authors are encouraged to cite the following recent, highly relevant articles to strengthen the literature background. Incorporating the following works will improve the article’s readability:

• https://doi.org/10.1016/j.rineng.2025.107054

• https://doi.org/10.1016/j.measurement.2025.120114

• https://doi.org/10.3390/ma15196661

• https://doi.org/10.1080/01694243.2023.2223367

• https://doi.org/10.1080/10426914.2021.1906897

7. Actually, the introduction is too short. It does not highlight the findings from previous studies. Also, it would be great if the authors added a literature review after completing the prior state of the art.

8. Table 1 lists “lumber” – presumably a typo for “plate”; please correct and ensure chemical composition ranges are accurately reported.

9. The method section does not specify whether the substrate was preheated or if interpass temperature was controlled, which critically affects heat accumulation and microstructure.

10. No information is provided on how many walls were fabricated, how many tensile/impact specimens per condition, or the statistical basis for the reported values.

11. Figures 3 and 4 show morphological data at 0, 400, 800, and 1600 W, but tensile and impact properties are only reported at 1100 W. The link between these laser powers is not discussed.

12. The explanation that reciprocating deposition reduces anisotropy is plausible, but no microstructural evidence (e.g., EBSD grain orientation maps) is provided to support the claim.

13. The impact energy values (48.5 and 52.2 kJ/m²) are an order of magnitude higher than typical Charpy V‑notch values for TC4. Please verify the test method and units.

14. First of all, it must be “Conclusions”. In its current form, the presented conclusions section doesn’t provide any concrete outcomes for readers. It is recommended to include the limitations and future work in the article.

Overall, the article is not so good and lacks clarity in its presentation. However, it can be improvised if worked upon. Therefore, I recommend MAJOR REVISION.

Reviewer #2: The following are comments about the text.

1) Improve the abstract using the adopted methodology.

2) The correct form is MPa. Check the spacing between all text values and the unit of measurement.

3) In introduction, is low thermal conductivity an excellent characteristic?

4) In introduction, the second paragraph is too long. It would be helpful to divide it into more paragraphs.

5) The introduction failed to better explain the objective of the work.

6) In Material and Methods, the figures and tables cited in the paragraph lack detail.

7) In Results and Discussions, the mechanism by which the additive manufacturing process influences the microstructure and mechanical properties of the alloy studied was not explained in detail. The literature should be used to clarify the results.

8) Where are the references from the last five years? It needs updating.

**********

what does this mean?). If published, this will include your full peer review and any attached files.

If you choose “no”, your identity will remain anonymous but your review may still be made public.

**Do you want your identity to be public for this peer review?**  For information about this choice, including consent withdrawal, please see our Privacy Policy

Reviewer #1: No

Reviewer #2: **Yes:** Givanildo Alves dos Santos

**********

[NOTE: If reviewer comments were submitted as an attachment file, they will be attached to this email and accessible via the submission site. Please log into your account, locate the manuscript record, and check for the action link "View Attachments". If this link does not appear, there are no attachment files.]

To ensure your figures meet our technical requirements, please review our figure guidelines: https://journals.plos.org/plosone/s/figures

You may also use PLOS’s free figure tool, NAAS, to help you prepare publication quality figures: https://journals.plos.org/plosone/s/figures#loc-tools-for-figure-preparation.

NAAS will assess whether your figures meet our technical requirements by comparing each figure against our figure specifications.

Attachment Submitted filename: PONE-D-26-14242.pdf

# Author comment: Author response to Decision Letter 1

**DOI:** 10.1371/journal.pone.0355487.r002

14 Jul 2026

Dear Editor and Reviewers:

Thanks for your comments on my manuscript “Study on microstructure and properties of titanium alloy wall fabricated by laser-TIG arc” (ID: PONE-D-26-14242). Those comments are all valuable and very helpful for revising and improving our paper, as well as the important guiding significance to our research. We have studied comments carefully and have made the correction which we hope meet with approval. All of the revisions have been made in red in the revised manuscript and point-by-point responses are follows:

Q1. Abstract is not well drafted. Write a structured abstract. The abstract must end with a conclusion and a recommendation from your work.

A1: Thank you very much for your comments and your comment is very suggestive.

Using TC4 titanium alloy welding wire as raw material, through laser-TIG arc hybrid heat source wire additive manufacturing technology, combined with reciprocating scanning method for deposition, the effects of different process parameters on the tensile properties, impact properties and hardness of additive specimens were studied. The results show that: (1) With the increase of laser power, the average layer width of the formed part increases from 7.1 mm to 12.1 mm, an increase of 59 %; the average layer height decreased from 1.95 mm to 1.56 mm, a decrease of 20 %. (2) When the laser power is 1100 W, the transverse tensile strength is 896 MPa, the longitudinal tensile strength is 883 MPa, and the difference between transverse and longitudinal tensile strength is 13 MPa. The elongation after transverse fracture is 9.31 %, the elongation after longitudinal fracture is 10.23 %, and the difference of elongation after transverse and longitudinal fracture is 0.92 %. (3) The impact energy of the additive samples at room temperature (25°C) in two directions is 48.5 J·cm-² (X direction) and 52.2K J·cm-² (Y direction), respectively, and the microhardness of the top of the wall is higher than that of the middle end of the wall. This study provides an important experimental basis for the engineering application of laser-TIG arc additive manufacturing technology of TC4 titanium alloy.

Q2. The introduction heavily cites papers from 2010 to 2022 but lacks references from 2024–2026, which weakens the study's motivation and currency.

A2: Thank you very much for your comments and your comment is very suggestive.

Introduction

Titanium and its alloys have a series of excellent properties, such as low density, high specific strength and specific stiffness, corrosion resistance and fatigue resistance, high temperature performance and good welding performance, and are praised as the third metal. With the rapid development of aviation and other industries, the requirements for titanium alloy performance indicators are becoming more and more stringent. In order to meet the use standards of key structural parts such as advanced aero-engines, the development of efficient preparation processes for titanium alloys has become one of the core research hotspots in the field of materials for a long time now and in the future[1-8].

Many scholars at home and abroad have done a lot of research on additive manufacturing technology. The study of Akerfeldt et al.[9] shows that the coarse columnarβgrains grown along the additive direction are one of the main factors affecting the anisotropy of the additive components. Edwards et al.[10] analyzed the effect of pores on the overall mechanical properties of the samples fabricated by laser deposition. Razavi et al.[11] used TSLM technology to prepare Ti-6Al-4V titanium alloy, and found that the surface quality of the workpiece produced by this method is high, and the defects are all on the surface of the workpiece. Brand et al.[12] carried out heat treatment on the subsequent laser additive manufacturing TC4 titanium alloy samples, and found that the mechanical properties of the samples were improved and the strength was improved. Baufeld et al.[13] found that the upper limit of fatigue strength of titanium alloys fabricated by additive manufacturing was increased by heat treatment. On this basis, Vrancken et al.[14] increased the temperature of heat treatment. When the transition temperature of a certain phase is reached, the mechanical properties of titanium alloys can be significantly improved. Zhang et al.[15] take a special temperature of titanium alloy annealing treatment, the sample, the material properties of uniform distribution, anisotropy has been significantly improved.

Pardal et al.[16] verified that the laser can stabilize the arc in the heat conduction mode, and the effective width utilization rate of the titanium alloy component is increased by 70.8 %, which significantly improves the forming quality of the component. Rezaei A et al.[17-18] found that the grain size of LPBF aluminum alloy in keyhole mode was smaller and the proportion of low-angle grain boundaries was higher, which promoted the PLC effect and achieved three times of plasticity improvement. Cai et al.[19] prepared AZ31 magnesium alloy by laser-arc coaxial composite additive manufacturing technology. The average tensile strength in the horizontal and vertical directions is 224 MPa and 222 MPa, respectively, which is isotropic. Compared with the traditional single heat source additive manufacturing technology, the laser-TIG arc hybrid additive manufacturing technology can significantly improve the manufacturing efficiency, improve the microstructure morphology, refine the grains, and improve the comprehensive performance of the sample. Obviously, the laser-arc hybrid process has a significant positive impact on the forming quality, microstructure and properties of titanium alloy, stainless steel, aluminum alloy and other materials. However, there are few reports on the research of laser-arc hybrid additive manufacturing of titanium alloy.

In this thesis, TC4 titanium alloy, which is widely used in the field of aerospace engineering, is used as the main test material. Through the laser-TIG arc hybrid heat source wire additive manufacturing technology, combined with the reciprocating scanning method for single-layer deposition, the mechanism and influence of different process parameters on the tensile properties, impact properties and hardness of the additive test piece are studied. In the cause of solve the problems of difficult forming of titanium alloy and difficult processing of precision structural parts, the regulation of process parameters on the forming morphology and deposition efficiency of thin-walled dimensions is realized.

References

[1] S.Vijayakumar, Optimization of friction stir welding parameters for dissimilar aluminium alloys using RSM-GRA and RSM-TOPSIS: Towards sustainable manufacturing in industry 4.0, Rineng, 000, 27(2025). https://doi.org/10.1016/j.rineng.2025.107054

[2] D. Bandhu, Multiobjective optimization of RMD welding parameters for ASTM A387 steel using Taguchi method and artificial neural networks, Meas, 120114, 262(2026). https://doi.org/10.1016/j.measurement.2025.120114

[3] D. Bandhu, F. Djavanroodi, G. Shaikshavali, Effect of metal-cored filler wire on surface morphology and micro-hardness of regulated metal deposition welded ASTM A387-Gr.11-Cl.2 steel plates, Materials. 19, 15(2022). https://doi.org/10.3390/ma15196661

[4] A.Yadav, M. Srivastava, P. Jain. Investigation of bead morphology and mechanical behaviour for metal inert gas welding-based WAAM in pulsed mode metal transfer on 316LSi stainless steel, J. Adhes. Sci. Technol. 5, 38(2024). https://doi.org/10.1080/01694243.2023.2241642

[5] D. Bandhu, K. Abhishek, Assessment of weld bead geometry in modified shortcircuiting gas metal arc welding process for low alloy steel. Mater. Manuf. Processes. 12, 36(2021). https://doi.org/10.1080/10426914.2021.1906897

[6] S. Semiatin, An overview of the thermomechanical processing of α/β titanium alloys: current status and future research opportunities, Metall. Mater. Trans. A. 51, 2593 (2020). https://link.springer.com/article/10.1007/s11661-020-05625-3

[7] M. Motyka, Martensite formation and decomposition during traditional and AM processing of two phase titanium alloys-an overview, Metals. 3, 11 (2021). https://www.mdpi.com/2075-4701/11/3/481

[8] Y. Hao, Y. Huang, K. Zhao, Research on the microstructure and mechanical properties of doubled annealed laser melting deposition TC11 titanium alloy, Opt. Laser. Technol. 5, 150 (2022). https://www.sciencedirect.com/science/article/abs/pii/S0030399222001402

[9] P. Akerfeldt, M. L. Antti, R. Pederson, Influence of microstructure on mechanical properties of laser metal wire-deposited Ti-6Al-4V, Mat. Sci. Eng. A-Struct. 674, 428 (2016). https://www.sciencedirect.com/science/article/abs/pii/S0921509316307924

[10] P. Edwards, M. Ramulu, Fatigue performance evaluation of selective laser melted Ti-6Al-4V, Mat Sci Eng. 598, 327 (2014). https://www.sciencedirect.com/science/article/abs/pii/S0921509314000720

[11] S. M. Razavi, P. Ferro, F. Berto, Fatigue strength of blunt v-notched sppeciens produced by selective laser melting of Ti-6Al-4V, Theor. Appl. Fract. Mec. 97, 376 (2017). https://www.sciencedirect.com/science/article/abs/pii/S0167844217302884

[12] E. Brandl, F. Palm, V. Michailov, Mechanical properties of additive manufactured titanium (Ti-6Al-4V) blocks deposited by a solid-state laser and wire, Mater. Design. 32, 10 (2011). https://www.sciencedirect.com/science/article/abs/pii/S0261306911004705

[13] B. Baufeld, E. Brandl. Wire based additive layer manufacturing: Comparison of microstructure and mechanical properties of Ti-6Al-4V components fabricated by laser-beam deposition and shaped metal deposition, J. Mater. Process. Tech. 211, 6 (2011). https://www.sciencedirect.com/science/article/abs/pii/S0924013611000306

[14] B. Vrancken, L. Thijs, J. P. Kruth, Heat treatment of Ti6Al4V produced by Selective Laser Melting: Microstructure and mechanical properties, J. Alloy. Compd. 541, 177 (2012). https://www.sciencedirect.com/science/article/abs/pii/S0925838812011826

[15] Q. Zhang, J. Chen, Zhao Z, Microstructure and anisotropic tensile behavior of laser additive manufactured TC21 titanium alloy, Mat. Sci. Eng. A-Struct. 673, 204 (2016). https://www.sciencedirect.com/science/article/abs/pii/S092150931630795X

[16] G. PARDAL, F. MARTINA, S. WILLIAMS, Laser stabilization of GMAW additive manufacturing of Ti-6Al4V components, J. Mater. Process. Technol., 8, 272 (2019). https://www.10.1016/j.jmatprotec.2019.04.036

[17] A. REZAEI, K. M. Keshavarz, S. Patel, Microstructural tailoring to promote Portevin-Le Chatelier effect in laser powder bed fusion fabricated AlMgScZr alloy, Mat Sci Eng A-Struct, 3, 95 (2026). https://www.10.1016/j.msea.2025.149708

[18] A. REZAEI, K. M. Keshavarz, Melting mode-driven processing diagram for nanoparticle enhanced high-strength aluminum alloy processed by laser powder bed fusion, Mater. Des., 2, 25 (2025). https://www. DOI:10.1016/j.matdes.2025.113794

[19] X. Cai, Y. XU, X.FENG, Laser-arc coaxial hybrid additive manufacturing of fine-grained homogeneous AZ31 magnesium alloy, J. Manuf. Process., 157, 2 (2026). https://www. DOI:10.1016/j.jmapro.2025.12.013

[20] C. Fu, Y. Yin, Study on microstructure and properties of TC4 titanium alloy by laser coaxial wire feeding additive manufacturing, Electric welding machine, 55, 2 (2025). https://www. 10.7512/j.issn.1001-2303.2025.02.08

[21] C. Wang, Finite element simulation and experimental analysis of metal TC4 additive manufacturing defects，xi'an university of technology, 2024.

Q3. The statement that “many scholars at home and abroad have been unable to meet the development needs” is overly vague and needs specific, quantifiable gaps to justify the hybrid approach.

A3: Thank you very much for your comments and your comment is very suggestive.

Many scholars at home and abroad have done a lot of research on additive manufacturing technology. The study of Akerfeldt et al.[9] shows that the coarse columnarβgrains grown along the additive direction are one of the main factors affecting the anisotropy of the additive components. Edwards et al.[10] analyzed the effect of pores on the overall mechanical properties of the samples fabricated by laser deposition. Razavi et al.[11] used TSLM technology to prepare Ti-6Al-4V titanium alloy, and found that the surface quality of the workpiece produced by this method is high, and the defects are all on the surface of the workpiece. Brand et al.[12] carried out heat treatment on the subsequent laser additive manufacturing TC4 titanium alloy samples, and found that the mechanical properties of the samples were improved and the strength was improved. Baufeld et al.[13] found that the upper limit of fatigue strength of titanium alloys fabricated by additive manufacturing was increased by heat treatment. On this basis, Vrancken et al.[14] increased the temperature of heat treatment. When the transition temperature of a certain phase is reached, the mechanical properties of titanium alloys can be significantly improved. Zhang et al.[15] take a special temperature of titanium alloy annealing treatment, the sample, the material properties of uniform distribution, anisotropy has been significantly improved.

Pardal et al.[16] verified that the laser can stabilize the arc in the heat conduction mode, and the effective width utilization rate of the titanium alloy component is increased by 70.8 %, which significantly improves the forming quality of the component. Rezaei A et al.[17-18] found that the grain size of LPBF aluminum alloy in keyhole mode was smaller and the proportion of low-angle grain boundaries was higher, which promoted the PLC effect and achieved three times of plasticity improvement. Cai et al.[19] prepared AZ31 magnesium alloy by laser-arc coaxial composite additive manufacturing technology. The average tensile strength in the horizontal and vertical directions is 224 MPa and 222 MPa, respectively, which is isotropic. Compared with the traditional single heat source additive manufacturing technology, the laser-TIG arc hybrid additive manufacturing technology can significantly improve the manufacturing efficiency, improve the microstructure morphology, refine the grains, and improve the comprehensive performance of the sample. Obviously, the laser-arc hybrid process has a significant positive impact on the forming quality, microstructure and properties of titanium alloy, stainless steel, aluminum alloy and other materials. However, there are few reports on the research of laser-arc hybrid additive manufacturing of titanium alloy.

In this thesis, TC4 titanium alloy, which is widely used in the field of aerospace engineering, is used as the main test material. Through the laser-TIG arc hybrid heat source wire additive manufacturing technology, combined with the reciprocating scanning method for single-layer deposition, the mechanism and influence of different process parameters on the tensile properties, impact properties and hardness of the additive test piece are studied. In the cause of solve the problems of difficult forming of titanium alloy and difficult processing of precision structural parts, the regulation of process parameters on the forming morphology and deposition efficiency of thin-walled dimensions is realized.

Q4. The introduction fails to clearly define what unique advantage laser TIG hybrid offers over standalone laser or arc additive manufacturing for TC4 walls.

A4: Thank you very much for your comments and your comment is very suggestive.

Pardal et al.[16] verified that the laser can stabilize the arc in the heat conduction mode, and the effective width utilization rate of the titanium alloy component is increased by 70.8 %, which significantly improves the forming quality of the component. Rezaei A et al.[17-18] found that the grain size of LPBF aluminum alloy in keyhole mode was smaller and the proportion of low-angle grain boundaries was higher, which promoted the PLC effect and achieved three times of plasticity improvement. Cai et al.[19] prepared AZ31 magnesium alloy by laser-arc coaxial composite additive manufacturing technology. The average tensile strength in the horizontal and vertical directions is 224 MPa and 222 MPa, respectively, which is isotropic. Compared with the traditional single

Attachment Submitted filename: PONE-D-26-14242-Response--2.docx

# Editor report: Decision Letter 1

**DOI:** 10.1371/journal.pone.0355487.r003

**Authors:** Wislei Riuper Osório

15 Jul 2026

Dear Dr. guo,

Thank you for submitting your manuscript to PLOS ONE. After careful consideration, we feel that it has merit but does not fully meet PLOS ONE’s publication criteria as it currently stands. Therefore, we invite you to submit a revised version of the manuscript that addresses the points raised during the review process.

Although a great number of improvements are clearly observed, there are other modification to be adopted/improved before its final publication. Although, the manuscript deserves its publication, there modifications are obligatory.

1. All text in red highlighted should be revised in order to solve Grammar and Spelling errors;
2. Into the section 2 and into Results and Discussion, all values and dimensions should obligatory be accompanied with their corresponding error ranges.
3. New sentences into section 2 and, in same cases into Results and discussion, should be included in order to elucidate the repeatibility and reproducibility of the proposed investigation.
4. Table 5 should de reworked and the corresponding error ranges adopted.
5. Figs. 2 and 3 and Figs. 4 and 5 should be reworked into a unique panel, respectively. Besides, its caption should be revised and indications “a” and “b” should be depicted. These figures can be disposed side by side.
7. Figs. 5, 6 and 7 should obligatory be revised and reworked. Error ranges should be adopted. When these error ranges are included, a new discussion seems to be resulted. Based on this, it is hardly suggested that the discussion be meticulously revised, as a function of the inclusion of the error ranges. It seems that similar behavior is occurred. Please, revised it.
7.In Fig. 7 error ranges and average line should be depicted in figure. Additionally, it is hardly suggested that at least one representative or typical image after indentation inside figure be also depicted.
8. Fig. 5 has remained error in scale “Mpa”.
Since Fig. 4 shows the tensile behavior of the examined samples, it is mandatory that its resulting stress vs. strain curve de depicted. This implies that a new figure be adopted. For this purpose, stress vs strain curves with duplicate should be depicted. Complementary, the proposed bar graph type can be depicted inside figure.
10. When a stress vs. strain curve is depicted with its reproducibility (duplicate or triplicate), another important parameter is revealed, i.e. the yield strength. This was neglected in discussion. Please, revise this point and include it.
11. Also, into the list of references, it is clearly verified that no guidelines was adopted. Please, revise it. For instance , upper letter is mixed with lower letter.

Please submit your revised manuscript by Aug 29 2026 11:59PM. If you will need more time than this to complete your revisions, please reply to this message or contact the journal office at plosone@plos.org. When you're ready to submit your revision, log on to https://www.editorialmanager.com/pone/ and select the 'Submissions Needing Revision' folder to locate your manuscript file.

- A letter that responds to each point raised by the academic editor and reviewer(s). You should upload this letter as a separate file labeled 'Response to Reviewers'.
- A marked-up copy of your manuscript that highlights changes made to the original version. You should upload this as a separate file labeled 'Revised Manuscript with Track Changes'.
- An unmarked version of your revised paper without tracked changes. You should upload this as a separate file labeled 'Manuscript'.

If you would like to make changes to your financial disclosure, please include your updated statement in your cover letter. Guidelines for resubmitting your figure files are available below the reviewer comments at the end of this letter.

If applicable, we recommend that you deposit your laboratory protocols in protocols.io to enhance the reproducibility of your results. Protocols.io assigns your protocol its own identifier (DOI) so that it can be cited independently in the future. For instructions see: https://journals.plos.org/plosone/s/submission-guidelines#loc-laboratory-protocols. Additionally, PLOS ONE offers an option for publishing peer-reviewed Lab Protocol articles, which describe protocols hosted on protocols.io. Read more information on sharing protocols at https://plos.org/protocols?utm_medium=editorial-email&utm_source=authorletters&utm_campaign=protocols.

As the corresponding author, your ORCID iD is verified in the submission system and will appear in the published article. PLOS supports the use of ORCID, and we encourage all coauthors to register for an ORCID iD and use it as well. Please encourage your coauthors to verify their ORCID iD within the submission system before final acceptance, as unverified ORCID iDs will not appear in the published article. *Only*  the individual author can complete the verification step; PLOS staff *cannot*  verify ORCID iDs on behalf of authors.

We look forward to receiving your revised manuscript.

Kind regards,

Wislei Riuper Osório

Academic Editor

PLOS One

Journal Requirements:

If the reviewer comments include a recommendation to cite specific previously published works, please review and evaluate these publications to determine whether they are relevant and should be cited. There is no requirement to cite these works unless the editor has indicated otherwise.

Additional Editor Comments:

Although a great number of improvements are clearly observed, there are other modification to be adopted/improved before its final publication. Although, the manuscript deserves its publication, there modifications are obligatory.

1. All text in red highlighted should be revised in order to solve Grammar and Spelling errors;

2. Into the section 2 and into Results and Discussion, all values and dimensions should obligatory be accompanied with their corresponding error ranges.

3. New sentences into section 2 and, in same cases into Results and discussion, should be included in order to elucidate the repeatibility and reproducibility of the proposed investigation.

4. Table 5 should de reworked and the corresponding error ranges adopted.

5. Figs. 2 and 3 and Figs. 4 and 5 should be reworked into a unique panel, respectively. Besides, its caption should be revised and indications “a” and “b” should be depicted. These figures can be disposed side by side.

7. Figs. 5, 6 and 7 should obligatory be revised and reworked. Error ranges should be adopted. When these error ranges are included, a new discussion seems to be resulted. Based on this, it is hardly suggested that the discussion be meticulously revised, as a function of the inclusion of the error ranges. It seems that similar behavior is occurred. Please, revised it.

7.In Fig. 7 error ranges and average line should be depicted in figure. Additionally, it is hardly suggested that at least one representative or typical image after indentation inside figure be also depicted.

8. Fig. 5 has remained error in scale “Mpa”.

Since Fig. 4 shows the tensile behavior of the examined samples, it is mandatory that its resulting stress vs. strain curve de depicted. This implies that a new figure be adopted. For this purpose, stress vs strain curves with duplicate should be depicted. Complementary, the proposed bar graph type can be depicted inside figure.

10. When a stress vs. strain curve is depicted with its reproducibility (duplicate or triplicate), another important parameter is revealed, i.e. the yield strength. This was neglected in discussion. Please, revise this point and include it.

11. Also, into the list of references, it is clearly verified that no guidelines was adopted. Please, revise it. For instance , upper letter is mixed with lower letter.

[Note: HTML markup is below. Please do not edit.]

[NOTE: If reviewer comments were submitted as an attachment file, they will be attached to this email and accessible via the submission site. Please log into your account, locate the manuscript record, and check for the action link "View Attachments". If this link does not appear, there are no attachment files.]

To ensure your figures meet our technical requirements, please review our figure guidelines: https://journals.plos.org/plosone/s/figures

You may also use PLOS’s free figure tool, NAAS, to help you prepare publication quality figures: https://journals.plos.org/plosone/s/figures#loc-tools-for-figure-preparation.

NAAS will assess whether your figures meet our technical requirements by comparing each figure against our figure specifications.

# Author comment: Author response to Decision Letter 2

**DOI:** 10.1371/journal.pone.0355487.r004

21 Jul 2026

Dear Editor and Reviewers:

Thanks for your comments on my manuscript “Study on microstructure and properties of titanium alloy wall fabricated by laser-TIG arc” (ID: PONE-D-26-14242). Those comments are all valuable and very helpful for revising and improving our paper, as well as the important guiding significance to our research. We have studied comments carefully and have made the correction which we hope meet with approval. All of the revisions have been made in red in the revised manuscript and point-by-point responses are follows:

1. All text in red highlighted should be revised in order to solve Grammar and Spelling errors;

A1: Thank you very much for your comments and your comment is very suggestive. All revisions are done in red in the revised manuscript.

2. Into the section 2 and into Results and Discussion, all values and dimensions should obligatory be accompanied with their corresponding error ranges.

A2: Thank you very much for your comments and your comment is very suggestive.

The hardness test uses the German KB automatic Vickers hardness tester to test the microhardness of the section of the sample after grinding and polishing. According to GB/T4340.1-2009, the loading force used in the test is 200 g. The standard tensile specimens (parallel section 5 mm) were prepared according to GB/T 228.1-2021 in both vertical and horizontal directions, and the tensile test was carried out by INSTRON5985-250 kN material testing machine. V-shaped impact specimens (10 mm×10 mm×55 mm) were prepared according to GB/T 229-2020, and the impact test was carried out at room temperature (25 °C) by ZBC2602-B pendulum impact tester. For the microhardness test, three test points were taken at the same height of the sample to measure the hardness, and the average of the three test points was finally taken as the final test result. For tensile test and impact test, three samples are also tested respectively, and the average value of the three samples is the final test result.

3.2 Analysis of tensile properties of laser-TIG arc hybrid wire additive manufacturing wall

When the laser power is 1100W, the tensile properties of TC4 titanium alloy in both transverse (X) and longitudinal (Y) directions are discussed. The results are shown in Fig.3. From Figure 3, it can be seen that when the laser power is 1100W, the transverse tensile strength is 896 MPa, the longitudinal tensile strength is 883 MPa, and the difference between the transverse and longitudinal tensile strength is 13 MPa; the transverse elongation after fracture is 9.31 %, the longitudinal elongation after fracture is 10.23 %, and the difference between transverse and longitudinal elongation after fracture is 0.92 %, indicating that the anisotropy of tensile properties is small. This low anisotropy is mainly due to the reciprocating deposition method: the interlayer deposition directions are perpendicular to each other, so that the grain structures in different directions are similar, and the grain orientations are basically the same, thus reducing the direction dependence of mechanical properties. It can be seen from Fig.3 that the error range of the three parallel experiments is small, the experimental accuracy is high, and the experimental accuracy is high.

Fig.3 Analysis of tensile propertie: (a) tensile strength; (b) post-fracture elongation

3.3 Impact performance analysis of laser-TIG arc hybrid wire additive manufacturing wall

When the laser power is 1100W, the impact energy of the additive sample is measured at room temperature (25oC) in both X and Y directions, and the results are shown in Fig.5. It can be seen from Fig.5 that the impact energy of the additive sample at room temperature (25oC) in two directions is 48.5 J·cm-² (X direction) and 52.2 J·cm-² (Y direction), respectively, which meet the requirements of CCS material welding and specification 2023. According to Fig.5, the relative error of the three parallel experiments is small, and the experimental results have high experimental accuracy.The impact toughness of the additive specimen is low. The main reason is that TC4 titanium alloy is a dual-phase titanium alloy. In the process of additive solidification, the grain boundary β phase precipitated at the boundary of the acicular α phase becomes a weak phase during the impact process, which makes the intergranular fracture occur during the impact process, and the energy needed to be absorbed during the test is small. The results are consistent with those of C. Fu [20].

Fig.5 Impact properties of additive samples

3.4 Microhardness analysis of laser-TIG arc hybrid wire additive manufacturing wall

When the laser power is 1100W, the microhardness of the deposit layer at the top and middle of the wall is detected respectively. The hardness distribution of the deposit layer of the single-channel multi-layer forming wall is shown in Fig.6. From the diagram, it can be seen that the microhardness at the top of the wall is higher than that at the middle end of the wall. Due to the fast heat dissipation and cooling rate at the top of the wall, the primary α phase and martensite α phase are refined and the orientation is dispersed, and the hardness is higher than that of the middle accumulation layer. The hardness fluctuation is due to the difference in microstructure caused by the different heat input between the accumulation layer and the remelting zone. The accumulation layer is mainly composed of α and α+β, while the interlayer remelting zone is mainly composed of lamellar α phase, and the lamellar is thicker and the size is larger. Due to the fast heat dissipation and cooling rate at the top of the wall, the primary α phase and martensite α phase are refined and the orientation is dispersed, and the hardness is higher than that of the middle accumulation layer. The results are consistent with those of C. Wang [21]. According to Fig.6, the relative error of the three parallel experimental tests is small, and the experimental results have a small error with the average value, so the experimental results have high experimental accuracy.

Fig.6 The hardness distribution diagram of the center distance of the wall accumulation layer

3. New sentences into section 2 and, in same cases into Results and discussion, should be included in order to elucidate the repeatibility and reproducibility of the proposed investigation.

A3: Thank you very much for your comments and your comment is very suggestive.

The hardness test uses the German KB automatic Vickers hardness tester to test the microhardness of the section of the sample after grinding and polishing. According to GB/T4340.1-2009, the loading force used in the test is 200 g. The standard tensile specimens (parallel section 5 mm) were prepared according to GB/T 228.1-2021 in both vertical and horizontal directions, and the tensile test was carried out by INSTRON5985-250 kN material testing machine. V-shaped impact specimens (10 mm×10 mm×55 mm) were prepared according to GB/T 229-2020, and the impact test was carried out at room temperature (25 °C) by ZBC2602-B pendulum impact tester. For the microhardness test, three test points were taken at the same height of the sample to measure the hardness, and the average of the three test points was finally taken as the final test result. For tensile test and impact test, three samples are also tested respectively, and the average value of the three samples is the final test result.

3.2 Analysis of tensile properties of laser-TIG arc hybrid wire additive manufacturing wall

When the laser power is 1100W, the tensile properties of TC4 titanium alloy in both transverse (X) and longitudinal (Y) directions are discussed. The results are shown in Fig.3. From Figure 3, it can be seen that when the laser power is 1100W, the transverse tensile strength is 896 MPa, the longitudinal tensile strength is 883 MPa, and the difference between the transverse and longitudinal tensile strength is 13 MPa; the transverse elongation after fracture is 9.31 %, the longitudinal elongation after fracture is 10.23 %, and the difference between transverse and longitudinal elongation after fracture is 0.92 %, indicating that the anisotropy of tensile properties is small. This low anisotropy is mainly due to the reciprocating deposition method: the interlayer deposition directions are perpendicular to each other, so that the grain structures in different directions are similar, and the grain orientations are basically the same, thus reducing the direction dependence of mechanical properties. It can be seen from Fig.3 that the error range of the three parallel experiments is small, the experimental accuracy is high, and the experimental accuracy is high.

Fig.3 Analysis of tensile propertie: (a) tensile strength; (b) post-fracture elongation

3.3 Impact performance analysis of laser-TIG arc hybrid wire additive manufacturing wall

When the laser power is 1100W, the impact energy of the additive sample is measured at room temperature (25oC) in both X and Y directions, and the results are shown in Fig.5. It can be seen from Fig.5 that the impact energy of the additive sample at room temperature (25oC) in two directions is 48.5 J·cm-² (X direction) and 52.2 J·cm-² (Y direction), respectively, which meet the requirements of CCS material welding and specification 2023. According to Fig.5, the relative error of the three parallel experiments is small, and the experimental results have high experimental accuracy.The impact toughness of the additive specimen is low. The main reason is that TC4 titanium alloy is a dual-phase titanium alloy. In the process of additive solidification, the grain boundary β phase precipitated at the boundary of the acicular α phase becomes a weak phase during the impact process, which makes the intergranular fracture occur during the impact process, and the energy needed to be absorbed during the test is small. The results are consistent with those of C. Fu [20].

Fig.5 Impact properties of additive samples

3.4 Microhardness analysis of laser-TIG arc hybrid wire additive manufacturing wall

When the laser power is 1100W, the microhardness of the deposit layer at the top and middle of the wall is detected respectively. The hardness distribution of the deposit layer of the single-channel multi-layer forming wall is shown in Fig.6. From the diagram, it can be seen that the microhardness at the top of the wall is higher than that at the middle end of the wall. Due to the fast heat dissipation and cooling rate at the top of the wall, the primary α phase and martensite α phase are refined and the orientation is dispersed, and the hardness is higher than that of the middle accumulation layer. The hardness fluctuation is due to the difference in microstructure caused by the different heat input between the accumulation layer and the remelting zone. The accumulation layer is mainly composed of α and α+β, while the interlayer remelting zone is mainly composed of lamellar α phase, and the lamellar is thicker and the size is larger. Due to the fast heat dissipation and cooling rate at the top of the wall, the primary α phase and martensite α phase are refined and the orientation is dispersed, and the hardness is higher than that of the middle accumulation layer. The results are consistent with those of C. Wang [21]. According to Fig.6, the relative error of the three parallel experimental tests is small, and the experimental results have a small error with the average value, so the experimental results have high experimental accuracy.

Fig.6 The hardness distribution diagram of the center distance of the wall accumulation layer

4. Table 5 should de reworked and the corresponding error ranges adopted.

A4: Thank you very much for your comments and your comment is very suggestive. All revisions are done in red in the revised manuscript.

Table 5 The hardness distribution value of the deposited layer

Distance/mm Middle/HV Top/HV

the first sample the second sample the third sample the first sample the second sample the third sample

-5.0 380 383 384 420 422 423

-4.5 360 365 363 430 435 433

-4.0 355 359 360 425 430 426

-3.5 365 367 367 425 426 428

-3.0 380 382 383 430 432 434

-2.5 360 363 365 420 424 424

-2.0 385 389 388 405 410 409

-1.5 360 365 361 410 414 415

-1.0 380 382 384 415 420 418

-0.5 365 369 370 405 408 410

0.0 385 389 390 420 422 423

0.5 370 373 374 415 419 417

1.0 385 386 389 435 439 439

1.5 360 365 363 430 435 435

2.0 360 362 365 435 438 439

2.5 385 390 387 430 435 433

3.0 360 364 365 420 423 423

3.5 380 383 384 430 434 434

4.0 355 360 358 425 428 429

4.5 370 373 372 420 424 423

5.0 365 367 371 425 428 427

5.5 375 379 377 430 432 433

5. Figs. 2 and 3 and Figs. 4 and 5 should be reworked into a unique panel, respectively. Besides, its caption should be revised and indications “a” and “b” should be depicted. These figures can be disposed side by side.

A5: Thank you very much for your comments and your comment is very suggestive. All revisions are done in red in the revised manuscript.

Fig. 2 The effect of laser power : (a) the average layer width ; (b) the average layer height

Fig.3 Analysis of tensile propertie: (a) tensile strength; (b) post-fracture elongation

6. Figs. 5, 6 and 7 should obligatory be revised and reworked. Error ranges should be adopted. When these error ranges are included, a new discussion seems to be resulted. Based on this, it is hardly suggested that the discussion be meticulously revised, as a function of the inclusion of the error ranges. It seems that similar behavior is occurred. Please, revised it.

A6: Thank you very much for your comments and your comment is very suggestive. All revisions are done in red in the revised manuscript.

3.2 Analysis of tensile properties of laser-TIG arc hybrid wire additive manufacturing wall

When the laser power is 1100W, the tensile properties of TC4 titanium alloy in both transverse (X) and longitudinal (Y) directions are discussed. The results are shown in Fig.3. From Figure 3, it can be seen that when the laser power is 1100W, the transverse tensile strength is 896 MPa, the longitudinal tensile strength is 883 MPa, and the difference between the transverse and longitudinal tensile strength is 13 MPa; the transverse elongation after fracture is 9.31 %, the longitudinal elongation after fracture is 10.23 %, and the difference between transverse and longitudinal elongation after fracture is 0.92 %, indicating that the anisotropy of tensile properties is small. This low anisotropy is mainly due to the reciprocating deposition method: the interlayer deposition directions are perpendicular to each other, so that the grain structures in different directions are similar, and the grain orientations are basically the same, thus reducing the direction dependence of mechanical properties. It can be seen from Fig.3 that the error range of the three parallel experiments is small, the experimental accuracy is high, and the experimental accuracy is high.

Fig.3 Analysis of tensile propertie: (a) tensile strength; (b) post-fracture elongation

3.3 Impact performance analysis of laser-TIG arc hybrid wire additive manufacturing wall

When the laser power is 1100W, the impact energy of the additive sample is measured at room temperature (25oC) in both X and Y directions, and the results are shown in Fig.5. It can be seen from Fig.5 that the impact energy of the additive sample at room temperature (25oC) in two directions is 48.5 J·cm-² (X direction) and 52.2 J·cm-² (Y direction), respectively, which meet the requirements of CCS material welding and specification 2023. According to Fig.5, the relative error of the three parallel experiments is small, and the experimental results have high experimental accuracy.The impact toughness of the additive specimen is low. The main reason is that TC4 titanium alloy is a dual-phase titanium alloy. In the process of additive solidification, the grain boundary β phase precipitated at the boundary of the acicular α phase becomes a weak phase during the impact process, which makes the intergranular fracture occur during the impact process, and the energy needed to be absorbed during the test is small. The results are consistent with those of C. Fu [20].

Fig.5 Impact properties

Attachment Submitted filename: PONE-D-26-14242-Response--3.docx

# Editor report: Decision Letter 2

**DOI:** 10.1371/journal.pone.0355487.r005

**Authors:** Wislei Riuper Osório

22 Jul 2026

Study on microstructure and properties of titanium alloy wall fabricated by laser-TIG arc

PONE-D-26-14242R2

Dear Dr. guo,

We’re pleased to inform you that your manuscript has been judged scientifically suitable for publication and will be formally accepted for publication once it meets all outstanding technical requirements.

Within one week, you’ll receive an e-mail detailing the required amendments. When these have been addressed, you’ll receive a formal acceptance letter and your manuscript will be scheduled for publication.

An invoice will be generated when your article is formally accepted. Please note, if your institution has a publishing partnership with PLOS and your article meets the relevant criteria, all or part of your publication costs will be covered. Please make sure your user information is up-to-date by logging into Editorial Manager at Editorial Manager® and clicking the ‘Update My Information' link at the top of the page. For questions related to billing, please contact billing support.

If your institution or institutions have a press office, please notify them about your upcoming paper to help maximize its impact. If they’ll be preparing press materials, please inform our press team as soon as possible -- no later than 48 hours after receiving the formal acceptance. Your manuscript will remain under strict press embargo until 2 pm Eastern Time on the date of publication. For more information, please contact onepress@plos.org.

Kind regards,

Wislei Riuper Osório

Academic Editor

PLOS One

Additional Editor Comments (optional):

Based on this second round of revision, the manuscript was meticulusly revised and improved. With this, it deserves its final publication. However, it hardly suggested that a revision considering Grammar and Speeling be provided. Also, the unit "Mpa"should be replaced with "MPa". This should be made in all proposed manuscript.

Reviewers' comments: No added on this stage.

# Editor report: Acceptance letter

**DOI:** 10.1371/journal.pone.0355487.r006

**Authors:** Wislei Riuper Osório

PONE-D-26-14242R2

PLOS One

Dear Dr. guo,

I'm pleased to inform you that your manuscript has been deemed suitable for publication in PLOS One. Congratulations! Your manuscript is now being handed over to our production team.

At this stage, our production department will prepare your paper for publication. This includes ensuring the following:

* All references, tables, and figures are properly cited

* All relevant supporting information is included in the manuscript submission,

* There are no issues that prevent the paper from being properly typeset

You will receive further instructions from the production team, including instructions on how to review your proof when it is ready. Please keep in mind that we are working through a large volume of accepted articles, so please give us a few days to review your paper and let you know the next and final steps.

Lastly, if your institution or institutions have a press office, please let them know about your upcoming paper now to help maximize its impact. If they'll be preparing press materials, please inform our press team within the next 48 hours. Your manuscript will remain under strict press embargo until 2 pm Eastern Time on the date of publication. For more information, please contact onepress@plos.org.

You will receive an invoice from PLOS for your publication fee after your manuscript has reached the completed accept phase. If you receive an email requesting payment before acceptance or for any other service, this may be a phishing scheme. Learn how to identify phishing emails and protect your accounts at https://explore.plos.org/phishing.

If we can help with anything else, please email us at customercare@plos.org.

Thank you for submitting your work to PLOS One and supporting open access.

Kind regards,

PLOS One Editorial Office Staff

on behalf of

Dr. Wislei Riuper Osório

Academic Editor

PLOS One
